# Synthesis of Substituted 1,4-Benzodiazepines by Palladium-Catalyzed Cyclization of *N*-Tosyl-Disubstituted 2-Aminobenzylamines with Propargylic Carbonates

**DOI:** 10.3390/molecules30143004

**Published:** 2025-07-17

**Authors:** Masahiro Yoshida, Saya Okubo, Akira Kurosaka, Shunya Mori, Touya Kariya, Kenji Matsumoto

**Affiliations:** 1 Faculty of Pharmaceutical Sciences, Tokushima Bunri University, 180 Nishihamabouji, Yamashiro-cho, Tokushima 770-8514, Japan; 2 Department of Engineering, Graduate School of Science and Engineering, Kagoshima University, 1-21-40 Korimoto, Kagoshima 890-0065, Japan; kmatsumoto@cb.kagoshima-u.ac.jp

**Keywords:** 1,4-benzodiazepine, cascade reaction, cyclization, palladium catalyst, propargylic carbonate

## Abstract

A synthesis of substituted 1,4-benzodiazepines has been developed via palladium-catalyzed cyclization of *N*-tosyl-disubstituted 2-aminobenzylamines with propargylic carbonates. The reaction proceeds through the formation of π-allylpalladium intermediates, which undergo intramolecular nucleophilic attack by the amide nitrogen to afford seven-membered benzodiazepine cores. In reactions involving unsymmetrical diaryl-substituted carbonates, regioselectivity was observed to favor nucleophilic attack at the alkyne terminus substituted with the more electron-rich aryl group, suggesting that electronic effects play a key role in determining product distribution. The versatility of this reaction was further demonstrated by constructing a benzodiazepine framework found in bioactive molecules, indicating its potential utility in medicinal chemistry. Mechanistic insights supported by stereochemical outcomes and X-ray crystallographic analysis of key intermediates reinforce the proposed reaction pathway. This palladium-catalyzed protocol thus offers an efficient and practical approach to access structurally diverse benzodiazepine derivatives.

## 1. Introduction

**Figure 1:**
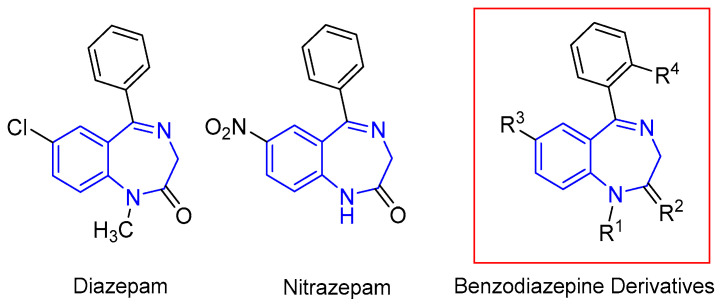
Structure of diazepam, nitrazepam, and benzodiazepine derivatives.

1,4-Benzodiazepines are privileged scaffolds in medicinal chemistry due to their broad spectrum of pharmacological activities. These compounds exhibit diverse effects on the central nervous system, including anxiolytic, anticonvulsant, antiepileptic, muscle relaxant, antidepressant, sedative, and hypnotic actions [1,2,3]. For instance, diazepam is commonly prescribed for anxiety and epilepsy, while nitrazepam is used to treat insomnia. Because of their therapeutic importance, a variety of synthetic methodologies have been developed to construct the 1,4-benzodiazepine framework (Figure 1) [4,5].

**Scheme 1:**
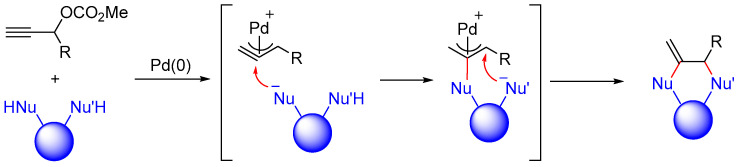
Palladium-catalyzed cyclization of propargylic carbonate with bis-nucleophile.

Palladium-catalyzed reactions involving propargylic carbonates and bis-nucleophiles—molecules bearing two nucleophilic sites within the same framework—have emerged as powerful strategies for the synthesis of cyclic compounds [6,7,8,9,10,11]. In these transformations, the reaction proceeds via the formation of a π-propargylpalladium intermediate, which undergoes nucleophilic attack to afford a π-allylpalladium complex. This intermediate subsequently undergoes an intramolecular reaction with the second nucleophilic site to furnish the cyclized product (Scheme 1) [12,13,14,15,16,17,18,19,20,21,22,23,24,25,26,27,28,29,30,31,32,33,34,35,36,37,38,39,40].

**Scheme 2:**
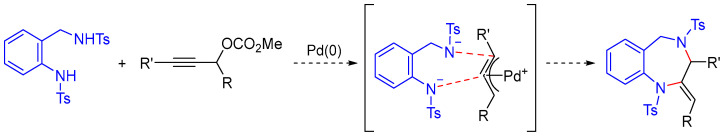
Strategy for the construction of 1,4-benzodiazepines.

During our investigation into palladium-catalyzed transformations of bis-nucleophiles with propargylic compounds [34,35,36,37,38,39,40], we became particularly interested in 2-aminobenzylamines substituted with electron-withdrawing groups on the nitrogen atom. We hypothesized that such molecules, possessing two nitrogen nucleophiles, could serve as ideal substrates for the intramolecular cyclization leading to 1,4-benzodiazepines (Scheme 2). Herein, we describe a palladium-catalyzed synthesis of substituted 1,4-benzodiazepines from *N*-tosyl-disubstituted 2-aminobenzylamines and propargylic carbonates. Notably, the reaction exhibits distinct regioselectivity depending on the substitution pattern of the carbonate.

## 2. Results and Discussion

We began our investigation by examining the reaction between *N*-tosyl-disubstituted 2-aminobenzylamine **1a** and phenyl-substituted propargylic carbonate **2a** under palladium catalysis. When the substrates were treated with 5 mol% Pd_2_(dba)_3_·CHCl_3_ and 20 mol% bis(diphenylphosphino)methane (DPPM) in dioxane at 50 °C for 2 h, the desired 1,4-benzodiazepine **(*Z*)-3a** and its geometric isomer **(*E*)-3a** were obtained in 21% combined yield with a *Z*:*E* ratio of 3:1 (Table 1, entry 1). Based on the examination with various bidentate ligands (entries 2–5), the use of 1,5-bis(diphenylphosphino)pentane (DPPPent) afforded the cyclized products in a combined yield of 51%. Furthermore, in the presence of the monodentate palladium complex Pd(PPh_3_)_4_, the yield was significantly improved to 98% (entry 6). Careful investigation of the reaction temperature (entries 7–9) revealed that at 25 °C, the cyclized products **(*Z*)-3a** and **(*E*)-3a** were obtained in a Z/E ratio of 3:1 and in 99% combined yield (entry 9). The structures of **(*Z*)-3a** and **(*E*)-3a** were determined by single-crystal X-ray diffraction analysis (CCDC 2454097 for **(*Z*)-3a**, CCDC 2455059 for **(*E*)-3a**).

**Figure 2:**
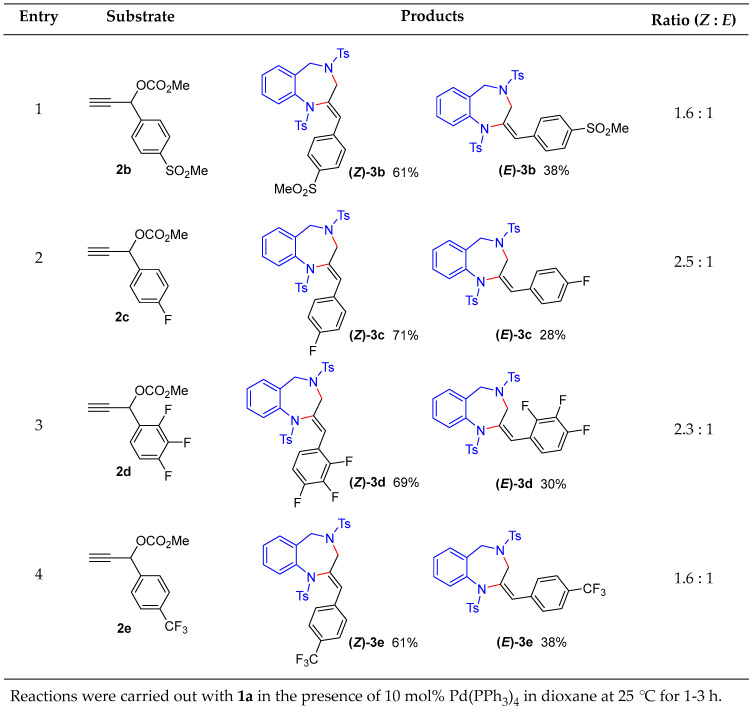
Attempts using various aryl-substituted propargylic carbonates **2b–e**.

We next examined a series of aryl-substituted propargylic carbonates **2b–2e** under the optimized conditions (Figure 2). Propargylic carbonate **2b**, bearing a 4-methanesulfonyl group as an electron-withdrawing substituent, afforded the corresponding benzodiazepine isomers **(*Z*)-3b** and **(*E*)-3b** in 61% and 38% yields, respectively, with a *Z*:*E* ratio of 1.6:1 (entry 1). Substrate **2c**, possessing a 4-fluoro group, gave **(*Z*)-3c** in 71% yield and **(*E*)-3c** in 28% yield, with a *Z*:*E* ratio of 2.5:1 (entry 2). Substrate **2d**, having a 2,3,4-trifluoro group, afforded **(*Z*)-3d** and **(*E*)-3d** in 69% and 30% yields, respectively, with a *Z*:*E* ratio of 2.3:1 (entry 3). Substrate **2e**, which features a 4-trifluoromethyl group as an electron-withdrawing substituent, provided **(*Z*)-3e** and **(*E*)-3e** in 61% and 38% yields, respectively, with a *Z*:*E* ratio of 1.6:1 (entry 4). In all cases, the (*Z*)-isomer was preferentially formed. The desired benzodiazepine products were obtained in nearly quantitative yields across all entries, underscoring the high efficiency of this palladium-catalyzed cyclization.

**Scheme 3:**
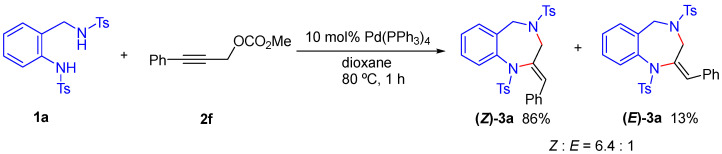
Reaction of **1a** with diphenyl-substituted propargylic carbonate **2f**.

As shown in Scheme 3, the reaction of substrate **2f**, bearing a phenyl group at the terminal alkyne position, was investigated. Interestingly, the use of this substrate led to the formation of the same cyclized products **(*Z*)-3a** and **(*E*)-3a** as those obtained from substrate **2a**, in which the substitution occurs at the propargylic position of the carbonate.

**Scheme 4:**
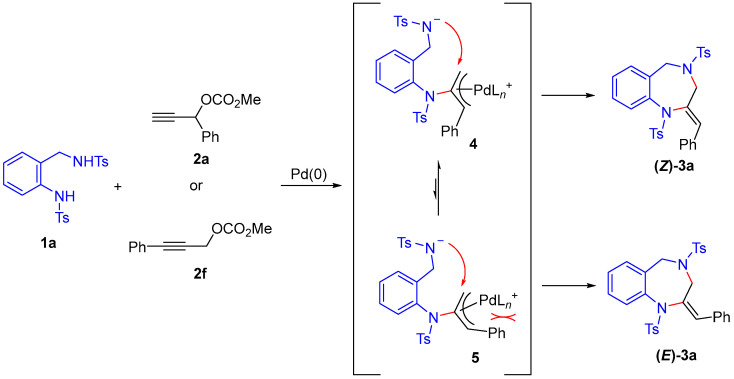
Proposed mechanism for the production of 1,4-benzodiazepines **3**.

A plausible mechanism for the formation of 1,4-benzodiazepines is depicted in Scheme 4. Upon coordination with palladium, propargylic carbonate **2a** undergoes decarboxylation to form the corresponding π-propargylpalladium complex. At this stage, the tosyl anilide unit, being a relatively soft nucleophile, selectively attacks the central carbon of the π-propargylpalladium species, leading to the formation of π-allylpalladium intermediates **4** and **5**. These two species are considered to be in a π–σ–π equilibrium, and the intramolecular nucleophilic attack by the benzyl amide anion is proposed to proceed predominantly from intermediate **4**, likely due to reduced steric hindrance compared to intermediate **5**, resulting in the formation of the cyclized product **(*Z*)-3a**. The observation that the reaction of propargylic carbonate **2f** also affords the same products, **(*Z*)-3a** and **(*E*)-3a**, supports the notion that both reactions proceed via the same π-allylpalladium intermediates **4** and **5**.

The results of reactions using diphenyl-substituted propargylic carbonate **2g** are summarized in Table 2. When **1a** was initially reacted with **2g** in the presence of Pd(PPh_3_)_4_, the product **6**, in which the tosyl anilide moiety had substituted the propargylic position, was obtained in 97% yield (entry 1). Subsequent examination of other catalytic systems revealed that the use of Pd_2_(dba)_3_·CHCl_3_ along with DPPM as the ligand led to the formation of **(*Z*)-7g**, a 1,4-benzodiazepine derivative, in 45% yield (entry 2). Interestingly, the product **(*Z*)-7g** is a positional isomer of the previously obtained cyclized products **3**, suggesting that it was formed via a distinct reaction pathway. Further examination of different bidentate phosphine ligands showed improved yields. When 1,2-bis(diphenylphosphino)ethane (DPPE) or 1,3-bis(diphenylphosphino)propane (DPPP) was employed, the yields of **(*Z*)-7g** increased significantly (entries 3 and 4), with the reaction in the presence of DPPP affording the product in an excellent 99% yield (entry 4). The structures of products **6** and **(*Z*)-7g** were unambiguously confirmed by single-crystal X-ray diffraction analysis (CCDC 2454077 for **6**, CCDC 2454078 for **(*Z*)-7g**).

**Scheme 5:**
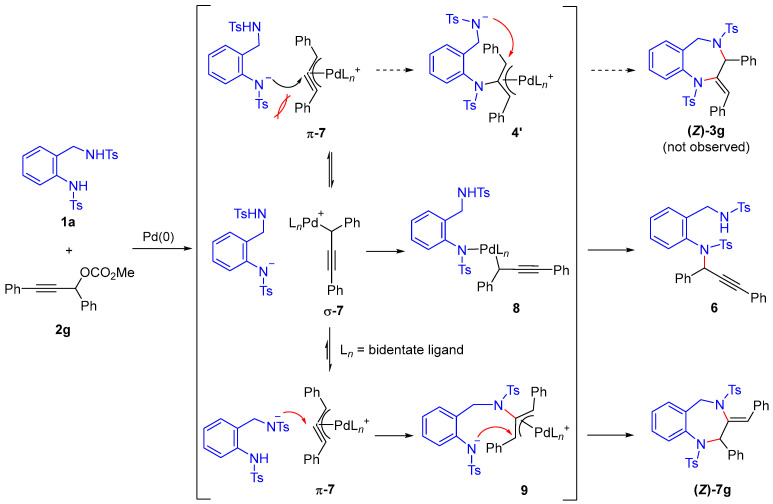
Proposed mechanism for the production of **6** and **(*Z*)-7g**.

A proposed mechanism for the formation of compounds **6** and **(*Z*)-7g** is shown in Scheme 5. Upon reaction with a palladium catalyst, propargylic carbonate **2** is transformed into the corresponding π-propargylpalladium complex **π-7**. In the case of diphenyl-substituted **π-7**, nucleophilic attack by the tosyl anilide nitrogen atom of **1a** is sterically hindered, and thus the expected cyclized product **(*Z*)-3g** was not formed. Instead, **π-7** is presumed to be in equilibrium with its σ-propargylpalladium isomer **σ-7**, which allows coordination of the tosyl anilide nitrogen to the palladium center to form intermediate **8**. Subsequent reductive elimination from this intermediate leads to the formation of the propargylic substitution product **6**. Under conditions employing bidentate phosphine ligands, this equilibrium is likely shifted toward the π-complex **π-7** [41,42], facilitating nucleophilic attack by the *N*-benzyl sulfonamide nitrogen—which experiences less steric hindrance—leading to the formation of the π-allylpalladium intermediate **9** [43]. This intermediate then undergoes intramolecular cyclization, resulting in the selective formation of 1,4-benzodiazepine derivative **(*Z*)-7g**.

**Scheme 6:**
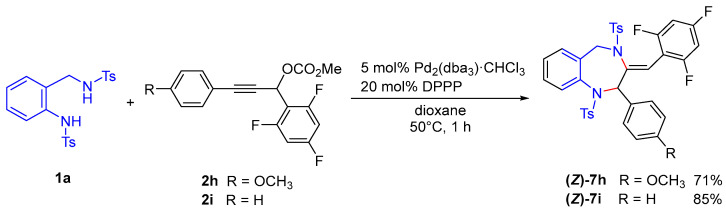
Reactions of **1a** with unsymmetrical diaryl-substituted carbonates **2h** and **2i**.

**Figure 3:**
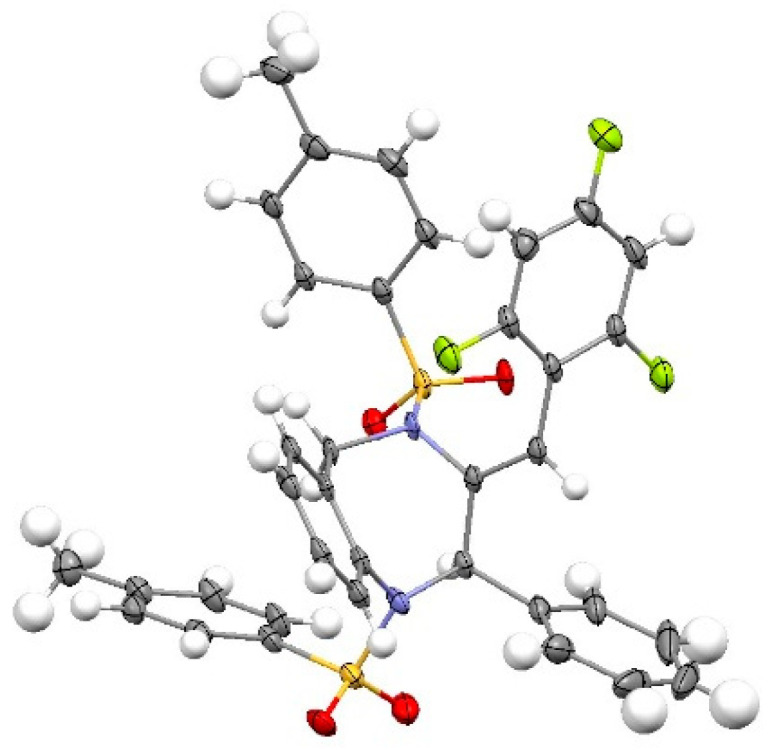
ORTEP drawing of **(*Z*)-7i**.

We next examined the reactions using propargylic carbonates **2h** and **2i**, each bearing two different aryl groups (Scheme 6). Treatment of **2h**, which has a 2,4,6-trifluorophenyl group at the propargylic position and a 4-methoxyphenyl group at the alkyne terminus, with **1a** proceeded smoothly to afford the 1,4-benzodiazepine product **(*Z*)-7h** with high regioselectivity in 71% yield. Similarly, when **2i**, bearing a phenyl group at the alkyne terminus, was employed, the corresponding cyclized product **(*Z*)-7i** was selectively obtained in 85% yield. The structure of **(*Z*)-7i** was confirmed by X-ray crystallographic analysis (CCDC 2454080) (Figure 3). The observed regioselectivity in these reactions is likely due to nucleophilic attack occurring preferentially at the more cationic carbon of the π-allylpalladium intermediate, which is substituted with the more electron-rich aryl group, as compared to the aryl group bearing an electron-withdrawing fluoro substituent. This electronic effect is believed to determine the selective formation of **(*Z*)-7h** and **(*Z*)-7i**.

**Scheme 7:**
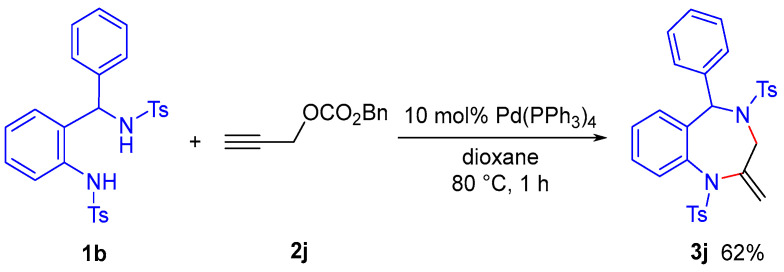
Construction of the core skeleton of benzodiazepine-based drugs.

Finally, we attempted the construction of the core skeleton of benzodiazepine-based drugs (Scheme 7). When propargylic carbonate **2j** was treated with substrate **1b**, which bears a phenyl group at the benzyl position, in the presence of a palladium catalyst, the expected cyclization proceeded smoothly to afford the cyclized product **3j** in 62% yield. The product **3j** contains the fundamental structure of benzodiazepine-class drugs, suggesting that this transformation could serve as an approach for synthesizing derivatives of such compounds.

## 3. Materials and Methods

All commercially available reagents were used without further purification. All reactions were performed in glassware equipped with a septum under positive argon pressure. The reaction mixture was magnetically stirred. Concentration was performed under reduced pressure. The heating experiments were conducted using an oil bath as a heat source. The reactions were monitored by TLC. TLC was performed on pre-coated plates (0.25 mm, silica gel 60F_245_, Merck & Co., Inc., Kenilworth, NJ, USA). Spots were visualized by exposure to UV light or by immersion in a solution of 10% phosphomolybdic acid in ethanol, followed by heating at ca. 200 °C. Column chromatography was performed on silica gel (40–50 μm, Kanto Chemical Co., Ltd., Nihonbashi, Tokyo, Japan). NMR spectra were recorded on a Bruker AVANCED III HD-500 (^1^H: 500 MHz, ^13^C: 125 MHz) spectrometer (Bruker Corporation, Billerica, USA) using tetramethylsilane (^1^H NMR at 0.00 ppm), CDCl_3_ (^13^C NMR at 77.16 ppm) and C_6_F_6_ (^19^F-NMR at −164.9 ppm) as a reference standard. Chemical shifts were reported in ppm. The following abbreviations were used to denote peak multiplicities: s, singlet; d, doublet; t, triplet; q, quartet; quin, quintet; sept, septet; m, multiplet; br, broadened. Mass spectra and high-resolution mass spectra were recorded on JEOL JMS-700 mass spectrometers (double-focusing magnetic sector) (JEOL Ltd., Tokyo, Japan).

*N*-Tosyl-disubstituted 2-aminobenzylamine **1a** [44] and propargylic carbonates **2a [34]**, **2c [38]**, **2e [45]**, **2f** [38], **2g [34]**, and **2j [46]** were prepared according to procedures described in the literature.

### 3.1. 4-Methyl-N-(2-(((4-methylphenyl)sulfonamido)(phenyl)methyl)phenyl)benzenesulfonamide (***1b***)

To a stirred solution of *N*-(2-benzoylphenyl)-4-methylbenzenesulfonamide [47] (858 mg, 2.44 mmol) and *p*-toluenesulfonamide (502 mg, 2.93 mmol) in 1,2-dichloroethane (20 mL) were added TiCl_4_ (0.16 mL, 1.46 mmol) and Et_3_N (0.68 mL, 4.88 mmol) dropwise. The reaction mixture was stirred under reflux for 13 h. After cooling to room temperature, the mixture was filtered through Celite and concentrated under reduced pressure. The residue was dissolved in EtOH (25 mL), and to the stirred solution was added NaBH_4_ (185 mg, 4.88 mmol) at 0 °C. The mixture was stirred for 7 h at the same temperature, then quenched with water and extracted with CH_2_Cl_2_. The combined organic layers were washed with brine, dried over anhydrous Na_2_SO_4_, and concentrated under reduced pressure. The crude product was recrystallized from CHCl_3_ and hexane to give *N*-tosyl-disubstituted 2-aminobenzylamine **1b** [48] as a white solid (805 mg, 1.59 mmol, 65% yield over two steps). Mp 145–151 °C. IR (ATR): 3272, 1597, 1487 cm^−1^; ^1^H-NMR (500 MHz, CDCl_3_) δ 2.35 (3H, s), 2.42 (3H, s), 5.02 (1H, d, *J* = 8.5 Hz), 5.39 (1H, d, *J* = 8.5 Hz), 6.63 (2H, d, *J* = 7.5 Hz), 6.72 (1H, dd, *J* = 1.5, 8.0 Hz), 6.99 (1H, td, *J* = 7.5, 1.0 Hz), 7.09 (4H, m), 7.14 (1H, m), 7.20 (2H, m), 7.27 (1H, m), 7.44 (1H, dd, *J* = 8.0, 1.0 Hz), 7.51 (2H, d, *J* = 8.5 Hz), 7.71 (2H, d, *J* = 8.5 Hz); ^13^C-NMR (125 MHz, CDCl_3_) δ 21.6 (CH_3_), 21.7(CH_3_), 56.8 (CH), 126.3 (CH), 126.4 (CH), 126.5 (CH), 127.0 (CH), 127.3 (CH), 127.5 (CH), 127.7 (CH), 128.6 (CH), 128.9 (CH), 129.1 (CH), 129.6 (CH), 129.9 (CH), 134.3 (Cq), 134.6 (Cq), 136.4 (Cq), 137.2 (Cq), 143.8 (Cq), 144.0 (Cq); HRMS (ES) *m/z* calcd for C_27_H_26_N_2_O_4_S_2_ [M]^+^ 506.1334, found 506.1333.

### 3.2. Methyl 1-[4-(Methylsulfonyl)phenyl]prop-2-yn-1-yl Carbonate (***2b***)

To a stirred solution of trimethylsilylacetylene (2.08 mL, 15.0 mmol) in THF (90 mL), *n*-BuLi (2.8 M in THF, 5.4 mL, 15.0 mmol) was added dropwise at −78 °C. The mixture was stirred for 0.5 h at the same temperature. A solution of 4-(methylsulfonyl)benzaldehyde (1.88 g, 10.0 mmol) in THF (15 mL) was then added dropwise at −78 °C, and stirring was continued for 1 h. Subsequently, methyl chloroformate (2.32 mL, 30.0 mmol) was added dropwise at −78 °C, and then the temperature was allowed to rise to room temperature over 1 h. The reaction mixture was diluted with water and extracted with AcOEt. The combined organic layers were washed with brine, dried, and concentrated. The residue was dissolved in THF (75 mL), and to the stirred solution were added TBAF (1 M in THF, 25.0 mL, 25.0 mmol) and AcOH (2.86 mL, 50.0 mmol). The mixture was stirred for 1 h at room temperature, then diluted with water and extracted with AcOEt. The combined organic layers were washed with brine, dried, and concentrated. The crude product was purified by column chromatography on silica gel (hexane/AcOEt = 75:25 *v*/*v*) to give propargylic carbonate **2b** as a colorless oil (2.54 g, 9.47 mmol, 95% yield in two steps). IR (ATR): 3262, 3020, 1749, 1598 cm^−1^; ^1^H-NMR (500 MHz, CDCl_3_) δ 2.78 (1H, d, *J* = 2.0 Hz) 3.06 (3H, s), 3.85 (3H, s), 6.35 (1H, d, *J* = 2.0 Hz), 7.76 (2H, d, *J* = 8.5 Hz), 7.99 (2H, d, *J* = 8.5 Hz); ^13^C-NMR (125 MHz, CDCl_3_) δ 44.6 (CH_3_), 55.6 (CH_3_), 68.3 (CH), 77.6 (CH), 78.6 (Cq), 128.1 (CH), 128.6 (CH), 141.5 (Cq), 141.8 (Cq), 154.7 (Cq); HRMS (ES) *m/z* calcd for C_12_H_12_O_5_S [M]^+^ 268.0405, found 268.0402.

### 3.3. Methyl 1-(2,3,4-Trifluorophenyl)prop-2-yn-1-yl Carbonate (***2d***)

Propargylic carbonate **2d** was prepared from 2,3,4-trifluorobenzaldehyde by following the same procedure described for **2b**, yielding 1.12 g (6.99 mmol, 78%) of a colorless oil over two steps. IR (ATR): 3296, 1752, 1514, 1486 cm^−1^; ^1^H-NMR (500 MHz, CDCl_3_) δ 2.74 (1H, d, *J* = 2.0 Hz), 3.84 (3H, s), 6.50 (1H, d, *J* = 2.0 Hz), 7.00–7.06 (1H, m), 7.41–7.46 (1H, m); ^13^C-NMR (125 MHz, CDCl_3_) δ 55.6 (CH_3_), 62.8 (CH, d, *J*_C-F_ = 4.0 Hz), 77.2 (CH), 77.9 (Cq), 112.6 (CH, dd, *J*_C-F_ = 3.0, 17.2 Hz), 121.1 (Cq, dd, *J*_C-F_ = 3.9, 10.8 Hz), 123.3 (CH, quintet, 6.9, 4.0 Hz), 138.9 (Cq, dt, *J*_C-F_ = 14.8, 251 Hz), 149.5 (Cq, ddd, *J*_C-F_ = 3.0, 10.8 and 254 Hz), 152.1 (Cq, ddd, *J*_C-F_ = 3.0, 9.9 and 251 Hz), 154.5 (Cq); ^19^F-NMR (376 MHz, CDCl_3_) δ −134.7–134.8 (m, 1F), −140.2–140.3 (m, 1F), −162.4–162.5 (m, 1F); HRMS (ES) *m/z* calcd for C_11_H_7_F_3_O_3_ [M]^+^ 244.0347, found 240.0344.

### 3.4. 3-(4-Methoxyphenyl)-1-(2,4,6-trifluorophenyl)prop-2-yn-1-yl Methyl Carbonate (***2h***)

To a stirred solution of *p*-ethynylanisole (0.61 mL, 4.68 mmol) in THF (16 mL), *n*-BuLi (2.8 M in THF, 1.80 mL, 4.68 mmol) was added dropwise at −78 °C. The mixture was stirred for 0.5 h at the same temperature. A solution of 2,4,6-trifluorobenzaldehyde (500 mg, 3.12 mmol) in THF (5 mL) was then added dropwise at −78 °C, and stirring was continued for 1 h. Subsequently, methyl chloroformate (0.72 mL, 9.36 mmol) was added dropwise at −78 °C, and then the temperature was allowed to rise to room temperature over 1 h. The reaction mixture was diluted with water and extracted with AcOEt. The combined organic layers were washed with brine, dried, and concentrated. The crude product was purified by column chromatography on silica gel (hexane/AcOEt = 75:25 *v*/*v*) to give propargylic carbonate **2h** as a colorless oil (1.02 g, 2.91 mmol, 93% yield). IR (ATR): 2960, 2226, 1751, 1604 cm^−1^; ^1^H-NMR (500 MHz, CDCl_3_) δ 3.80 (3H, s), 3.82 (3H, s), 6.72 (2H, t, *J* = 8.5 Hz), 6.78 (1H, s), 6.83 (2H, d, *J* = 8.5 Hz), 7.39 (2H, d, *J* = 8.5 Hz), ^13^C-NMR (125 MHz, CDCl_3_) δ 55.4 (CH_3_), 60.1 (CH_3_), 81.7 (Cq), 87.4 (Cq), 101.0 (CH, td, *J* = 2.9, 26.1 Hz), 110.5 (Cq, td, *J* = 4.9, 16.8 Hz), 113.8 (Cq), 114.1 (CH), 133.8 (CH), 154.7 (Cq), 160.3 (Cq, m), 160.4 (Cq), 162.4 (Cq, dd, *J* = 8.9, 13.8 Hz), 164.4 (Cq, t, *J* = 15.0 Hz); ^19^F-NMR (376 MHz, CDCl_3_) δ −108.2–108.3 (m, 1F), −112.0 (t, *J* = 6.9 Hz, 2F); HRMS (ES) *m/z* calcd for C_18_H_13_F_3_O_4_ [M]^+^ 350.0766, found 350.0769.

### 3.5. Methyl 3-Phenyl-1-(2,4,6-trifluorophenyl)prop-2-yn-1-yl Carbonate (***2i***)

By following the same procedure described for **2h**, propargylic carbonate **2i** was prepared from 2,3,4-trifluorobenzaldehyde in 99% yield (997 mg, 3.11 mmol) as a colorless oil. IR (ATR): 3088, 2960, 1752, 1635 cm^−1^; ^1^H-NMR (500 MHz, CDCl_3_) δ 3.83 (3H, s), 6.71 (2H, t, *J* = 8.0 Hz), 6.80 (1H, s), 7.29–7.36 (3H, m), 7.45 (2H, dd, *J* = 2.0, 8.0 Hz); ^13^C-NMR (125 MHz, CDCl_3_) δ 55.4 (CH_3_), 59.9 (CH), 82.9 (Cq), 87.2 (Cq), 100.8 (CH, td, *J* = 3.0, 25.6 Hz), 110.2 (Cq, dd, *J* = 4.9, 16.8 Hz), 121.8 (Cq), 128.4 (CH), 129.2 (CH), 132.2 (CH), 154.7 (Cq), 160.3 (Cq, dd, *J* = 8.9, 15.3 Hz), 162.4 (Cq, m), 164.4 (Cq, t, *J* = 14.8 Hz); ^19^F-NMR (376 MHz, CDCl_3_) δ −110.0–110.1 (m, 1F), −113.0 (t, *J* = 6.9 Hz, 2F); HRMS (ES) *m/z* calcd for C_17_H_11_F_3_O_3_ [M]^+^ 320.0660, found 320.0655.

### 3.6. Procedure for the Synthesis of 1,4-Benzodiazepines—Reaction of ***1a*** and ***2a*** (Table 1, Entry 9)

To a stirred solution of propargylic carbonate **2a** (29.9 mg, 123 µmol) in dioxane (0.5 mL) were added *N*-tosyl-disubstituted 2-aminobenzylamine **1a** (40.5 mg, 94.2 µmol) and Pd(PPh_3_)_4_ (10.9 mg, 9.4 µmol) at 25 °C. The reaction mixture was stirred for 3 h at the same temperature. After filtration through a small amount of silica gel and concentration under reduced pressure, the crude residue was purified by column chromatography on silica gel (hexane/AcOEt = 5:1 *v*/*v*) to afford the 1,4-benzodiazepines **(*Z*)-3a** (38.0 mg, 69.8 µmol, 74%) and **(*E*)-3a** (12.8 mg, 23.6 µmol, 25%) as colorless crystals.

### 3.7. (Z)-2-Benzylidene-1,4-ditosyl-2,3,4,5-tetrahydro-1H-benzo[e][1,4]diazepine [***(*Z*)-3a***]

Yield: 74% (38.0 mg, 69.8 µmol); colorless quadrilaterals (AcOEt, mp. 147–150 °C); IR (ATR): 2855, 1597, 1489 cm^−1^; ^1^H-NMR (500 MHz, CDCl_3_) δ 2.25 (3H, s), 2.35 (3H, s), 3.59 (1H, d, *J* = 15.0 Hz), 4.17 (1H, d, *J* = 15.0 Hz), 4.42 (1H, d, *J* = 15.0 Hz)–4.47 (1H, d, *J* = 15.0 Hz), 6.45 (1H, s), 7.01 (2H, d, *J* = 8.0 Hz), 7.05 (2H, d, *J* = 8.0 Hz), 7.17–7.22 (4H, m), 7.24–7.29 (7H, m), 7.36 (2H, d, *J* = 8.5 Hz); ^13^C-NMR (125 MHz, CDCl_3_) δ 21.5 (CH_3_), 21.7 (CH_3_), 51.8 (CH_2_), 55.8 (CH_2_), 144.1 (Cq), 143.5 (Cq), 138.3 (Cq), 136.8 (Cq), 136.1 (CH), 136.0 (Cq), 133.5 (Cq), 131.0 (Cq), 130.7 (CH), 129.6 (CH), 129.4 (CH), 129.0 (CH), 129.0 (CH), 128.5 (CH), 128.3 (CH), 128.2 (CH), 127.8 (CH), 127.6 (CH); HRMS (EI) *m/z* calcd for C_30_H_28_N_2_O_4_S_2_ [M]^+^ 544.1490, found 544.1496. For single-crystal data, see Supplementary Materials.

### 3.8. (E)-2-Benzylidene-1,4-ditosyl-2,3,4,5-tetrahydro-1H-benzo[e][1,4]diazepine [***(*E*)-3a***]

Yield: 25% (12.8 mg, 23.6 µmol); colorless quadrilaterals (AcOEt, mp. 165–171 °C); IR (ATR): 2364, 1597, 1490 cm^−1^; ^1^H-NMR (500 MHz, CDCl_3_) δ 2.38 (3H, s), 2.45 (3H, s), 4.29–4.31 (4H, m), 6.71 (1H, s), 7.07 (2H, d, *J* = 8.0 Hz), 7.23–7.40 (13H, m), 7.70 (2H, d, *J* = 8.0 Hz); ^13^C-NMR (125 MHz, CDCl_3_) δ 49.8 (CH_2_), 51.91 (CH_2_), 127.4 (CH), 127.8 (CH), 127.9 (CH), 128.3 (CH), 128.6 (CH), 128.7 (CH), 129.1 (CH), 129.3 (CH), 129.5 (CH), 129.8 (CH), 130.4 (CH), 133.1 (Cq), 133.8 (Cq), 135.3 (Cq), 135.7 (Cq), 137.3 (CH), 137.7 (Cq), 139.9 (Cq), 143.4 (Cq), 144.2 (Cq); HRMS (EI) *m/z* calcd for C_30_H_28_N_2_O_4_S_2_ [M]^+^ 544.1490, found 544.1497. For single-crystal data, see Supplementary Materials.

### 3.9. (Z)-2-(4-(Methylsulfonyl)benzylidene)-1,4-ditosyl-2,3,4,5-tetrahydro-1H-benzo[e][1,4]diazepine [***(*Z*)-3b***]

By following the same procedure described with respect to **3a**, 1,4-benzodiazepine **(*Z*)-3b** was prepared from **1a** and **2b**, with a yield of 61% (26.6 mg, 42.7 µmol) as a colorless oil. IR (ATR): 2925, 2855, 1597, 1489 cm^−1^; ^1^H-NMR (500 MHz, CDCl_3_) δ 2.29 (3H, s), 2.38 (3H, s), 3.08 (3H, s), 3.52 (1H, d, *J* = 15.5 Hz), 4.14 (1H, d, *J* = 11.5 Hz), 4.44–4.49 (2H, m), 6.53 (1H, s), 7.05 (2H, d, *J* = 8.0 Hz), 7.09 (2H, d, *J* = 8.0 Hz), 7.14 (2H, d, *J* = 8.0 Hz), 7.25–7.28 (4H, m), 7.39 (2H, d, *J* = 8.0 Hz), 7.44 (2H, d, *J* = 8.0 Hz), 7.86 (2H, d, *J* = 8.0 Hz); ^13^C-NMR (125 MHz, CDCl_3_) δ 21.6 (CH_3_), 21.7 (CH_3_), 44.6 (CH_3_), 51.9 (CH_2_), 55.4 (CH_2_), 127.3 (CH), 127.6 (CH), 127.7 (CH), 128.8 (CH), 129.3 (CH), 129.4 (CH), 129.5 (CH), 129.8 (CH), 130.9 (CH), 134.0 (CH), 134.3 (Cq), 136.1 (Cq), 136.4 (Cq), 137.9 (Cq), 139.2 (Cq), 140.1 (Cq), 143.7 (Cq), 144.7 (Cq); HRMS (EI) *m/z* calcd for C_31_H_30_N_2_O_6_S_3_ [M]^+^ 622.1266, found 622.1271.

### 3.10. (E)-2-(4-(Methylsulfonyl)benzylidene)-1,4-ditosyl-2,3,4,5-tetrahydro-1H-benzo[e][1,4]diazepine [***(*E*)-3b***]

By following the same procedure described with respect to **3a**, 1,4-benzodiazepine **(*E*)-3b** was prepared from **1a** and **2b**, with a yield of 38% (16.6 mg, 26.6 µmol), as a colorless oil. IR (ATR): 2924 2852, 1597, 1490 cm^−1^; ^1^H-NMR (500 MHz, CDCl_3_) δ 2.41 (3H, s), 2.44 (3H, s), 3.08 (3H, s), 4.15–4.16 (3H, m), 6.80 (1H, s), 7.17 (2H, d, *J* = 8.5 Hz), 7.25–7.29 (5H, m), 7.36 (3H, d, *J* = 8.0 Hz), 7.46 (2H, d, *J* = 8.0 Hz), 7.64 (2H, d, *J* = 8.5 Hz), 7.95 (2H, d, *J* = 8.5 Hz); ^13^C-NMR (125 MHz, CDCl_3_) δ 21.7 (CH_3_), 21.8 (CH_3_), 44.6 (CH_3_), 49.7 (CH_2_), 51.9 (CH_2_), 127.4 (CH), 127.8 (CH), 128.3 (CH), 128.8 (CH), 129.6 (CH), 129.8 (CH), 130.0 (CH), 130.0 (CH), 130.5 (CH), 134.4 (CH), 135.0 (Cq), 135.5 (Cq), 136.2 (Cq), 137.4 (Cq), 139.4 (Cq), 139.6 (Cq),140.3 (Cq), 143.9 (Cq), 144.5 (Cq); HRMS (EI) *m/z* calcd for C_31_H_30_N_2_O_6_S_3_ [M]^+^ 622.1266, found 622.1269.

### 3.11. (Z)-2-(4-Fluorobenzylidene)-1,4-ditosyl-2,3,4,5-tetrahydro-1H-benzo[e][1,4]diazepine [***(*Z*)-3c***]

By following the same procedure described with respect to **3a**, 1,4-benzodiazepine **(*Z*)-3c** was prepared from **1a** and **2c**, with a yield of 71% (28.0 mg, 50.0 µmol), as a colorless solid (AcOEt, mp. 151–155 °C). IR (ATR): 3023, 1596, 1510, 1488 cm^−1^; ^1^H-NMR (500 MHz, CDCl_3_) δ 2.27 (3H, s), 2.37 (3H, s), 3.49 (1H, d, *J* = 15.5 Hz), 4.14 (1H, d, *J* = 14.0 Hz, 1H), 4.38 (2H, d, *J* = 14.0 Hz), 4.44 (2H, d, *J* = 15.5 Hz), 6.40 (1H, s), 6.97 (2H, t, *J* = 8.5 Hz), 7.03 (2H, d, *J* = 8.5 Hz, 2H), 7.08 (2H, d, *J* = 8.5 Hz), 7.16–7.32 (8H, m), 7.36 (2H, d, *J* = 8.5 Hz); ^13^C-NMR (125 MHz, CDCl_3_) δ 21.5 (CH_3_), 21.7 (CH_3_), 51.8 (CH_2_), 55.6 (CH_2_), 115.4 (CH), 127.6 (CH), 127.7 (CH), 128.4 (CH, d, *J*_C-F_ = 7.9 Hz), 129.1 (CH, d, *J*_C-F_ = 14.8 Hz), 129.5 (CH), 129.5 (Cq, d, *J*_C-F_ = 3.0 Hz), 129.6 (CH), 130.7 (CH), 130.9 (CH, d, *J*_C-F_ = 7.9 Hz), 131.0 (Cq), 134.9 (CH), 135.9 (Cq), 136.1 (Cq), 136.5 (Cq), 138.1 (Cq), 143.5 (Cq), 144.3 (Cq), 162.8 (Cq, d, *J*_C-F_ = 248 Hz); ^19^F-NMR (376 MHz, CDCl_3_) δ −115.4 (s, 1F); HRMS (ESI) *m/z* calcd for C_30_H_27_FN_2_O_4_S_2_ [M]^+^ 562.1396, found 562.1403.

### 3.12. (E)-2-(4-Fluorobenzylidene)-1,4-ditosyl-2,3,4,5-tetrahydro-1H-benzo[e][1,4]diazepine [***(*E*)-3c***]

By following the same procedure described with respect to **3a**, 1,4-benzodiazepine **(*E*)-3c** was prepared from **1a** and **2c**, with a yield of 28% (11.0 mg, 19.6 µmol), as a colorless solid (AcOEt, mp. 165–169 °C). IR (ATR): 2922, 1601, 1508, 1490 cm^−1^; ^1^H-NMR (500 MHz, CDCl_3_) δ2.38 (3H, s), 2.43 (3H, s), 4.19–4.22 (4H, m), 6.66 (1H, s), 7.06 (2H, t, *J* = 8.5 Hz), 7.11 (2H, d, *J* = 8.0 Hz), 7.20–7.24 (4H, m), 7.27–7.33 (6H, m), 7.66 (2H, d, *J* = 8.0 Hz); ^13^C-NMR (125 MHz, CDCl_3_) δ 21.7 (CH_3_), 21.8 (CH_3_), 49.9 (CH_2_), 52.0 (CH_2_), 115.8 (CH, d, *J*_C-F_ = 21.8 Hz), 127.4 (CH), 127.8 (CH), 127.9 (CH), 128.5 (CH), 129.4 (CH), 129.7 (CH), 129.9 (CH), 130.5 (CH), 131.0 (CH, d, *J*_C-F_ = 7.9 Hz), 133.4 (Cq), 135.2 (Cq), 135.7 (Cq), 136.3 (Cq), 137.7 (Cq), 140.0 (Cq), 143.6 (Cq), 144.3(Cq), 162.8 (Cq, d, *J*_C-F_ = 249.7 Hz); ^19^F-NMR (376 MHz, CDCl_3_) δ −115.4–115.5 (m, 1F); HRMS (ES) *m/z* calcd for C_30_H_27_FN_2_O_4_S_2_ [M]^+^ 562.1396, found 562.1388.

### 3.13. (Z)-1,4-Ditosyl-2-(2,3,4-trifluorobenzylidene)-2,3,4,5-tetrahydro-1H-benzo[e][1,4]diazepine [***(*Z*)-3d***]

By following the same procedure described with respect to **3a**, 1,4-benzodiazepine **(*Z*)-3d** was prepared from **1a** and **2d**, with a yield of 69% (33.4 mg, 55.9 µmol), as colorless needles (AcOEt, mp. 175–179 °C). IR (ATR): 2925, 1598, 1471 cm^−1^; ^1^H-NMR (500 MHz, CDCl_3_) δ 2.27 (3H, s), 2.38 (3H, s), 3.62 (1H, m), 4.40 (1H, m), 4.43 (2H, m), 6.51 (1H, s), 6.91 (1H, q, *J* = 7.5 Hz), 7.05 (2H, d, *J* = 8.0 Hz), 7.11 (2H, d, *J* = 8.0 Hz), 7.21–7.30 (5H, m), 7.36–7.40 (4H, m); ^13^C-NMR (125 MHz, CDCl_3_) δ 21.5 (CH_3_), 21.6 (CH_3_), 51.7 (CH_2_), 55.4 (CH_2_), 111.7 (CH, dd, *J*_C-F_ 3.0, 17.8 Hz), 119.2 (Cq, dd, *J*_C-F_ = 4.0, 9.9 Hz), 123.9 (CH, m), 126.7 (CH), 127.5 (CH), 127.7 (CH), 128.5 (CH), 129.1 (CH), 129.5 (CH), 129.7 (CH), 130.8 (CH), 134.6 (Cq), 135.7 (Cq), 135.9 (Cq), 136.3 (Cq), 138.0 (Cq), 139.8 (Cq, dt, *J*_C-F_ = 15.9, 251.6 Hz), 143.6 (Cq), 144.6 (Cq), 149.2 (Cq, ddd, *J*_C-F_ = 4.0, 9.9, 252.6 Hz), 151.3 (Cq, ddd, *J*_C-F_ = 3.0, 8.9, 252.6 Hz); ^19^F-NMR (376 MHz, CDCl_3_) δ −136.2–136.3 (m, 1F), −137.4 (m, 1F), −163.7–163.8 (m, 1F); HRMS (EI) *m/z* calcd for C_30_H_25_F_3_N_2_O_4_S_2_ [M]^+^ 598.1208, found 598.1212.

### 3.14. (E)-1,4-Ditosyl-2-(2,3,4-trifluorobenzylidene)-2,3,4,5-tetrahydro-1H-benzo[e][1,4]diazepine [***(*E*)-3d***]

By following the same procedure described with respect to **3a**, 1,4-benzodiazepine **(*E*)-3d** was prepared from **1a** and **2d**, with a yield of 30% (14.5 mg, 24.3 µmol), as colorless needles (AcOEt, mp. 154–159 °C). IR (ATR): 2924, 1598, 1471 cm^−1^; ^1^H-NMR (500 MHz, CDCl_3_) δ 2.39 (3H, s), 2.45 (3H, s), 4.18 (2H, s), 4.28 (2H, s), 6.58 (1H, s), 6.99–7.06 (1H, m), 7.12–7.17 (3H, m), 7.3 (3H, m), 7.28–7.41 (8H, m), 7.71 (2H, d, *J* = 8.0 Hz); ^13^C-NMR (125 MHz, CDCl_3_) δ 21.6 (CH_3_), 21.8 (CH_3_), 50.0 (CH_2_), 51.9 (CH_2_), 112.6 (Cq, dd, *J*_C-F_ = 4.0, 17.8 Hz), 119.4 (Cq, dd, *J*_C-F_ = 4.0, 11.9 Hz), 124.6 (Cq, m), 127.3 (CH), 127.8 (CH), 127.9 (CH), 127.9 (CH), 128.8 (CH), 129.6 (CH), 129.7 (CH), 130.0 (CH), 130.5 (CH), 135.2 (Cq), 136.0 (Cq), 136.4 (Cq), 137.6 (Cq), 139.7 (Cq), 140.2 (Cq, d, *J*_C-F_ = 252.6 Hz), 144.0 (Cq), 144.5 (Cq), 151.4 (Cq, d, *J*_C-F_ = 254.6 Hz); ^19^F-NMR (376 MHz, CDCl_3_) δ −135.5–135.6 (m, 1F), −137.0–137.1 (m, 1F), −163.1–163.2 (m, 1F); HRMS (EI) *m/z* calcd for C_30_H_25_F_3_N_2_O_4_S_2_ [M]^+^ 598.1208, found 598.1212.

### 3.15. (Z)-1,4-Ditosyl-2-(4-(trifluoromethyl)benzylidene)-2,3,4,5-tetrahydro-1H-benzo[e][1,4]diazepine[***(*Z*)-3e***]

By following the same procedure described with respect to **3a**, 1,4-benzodiazepine **(*Z*)-3e** was prepared from **1a** and **2e**, with a yield of 61% (26.1 mg, 42.7 µmol), as colorless quadrilaterals (AcOEt, mp. 171–180 °C). IR (ATR): 3015, 1596, 1487 cm^−1^; ^1^H-NMR (500 MHz, CDCl_3_) δ 2.35–2.36 (6H, m), 3.67 (1H, d, *J* = 15.5 Hz), 4.17 (1H, d, *J* = 15.5 Hz), 4.47 (2H, d, *J* = 15.5 Hz), 4.53 (2H, d, *J* = 15.5 Hz), 6.50 (1H, s), 7.03 (2H, d, *J* = 5.0 Hz), 7.04 (2H, d, *J* = 5.0 Hz), 7.09 (1H, d, *J* = 7.0 Hz), 7.14–7.18 (3H, m), 7.22–7.30 (4H, m), 7.38 (2H, d, *J* = 8.0 Hz), 7.52 (2H, d, *J* = 8.0 Hz); ^13^C-NMR (125 MHz, CDCl_3_) δ 21.5 (CH_3_), 21.6 (CH_3_), 52.0 (CH_2_), 55.7 (CH_2_), 123.0 (Cq), 125.1 (CH, d, *J*_C-F_ = 4.0 Hz), 127.7 (CH, d, *J*_C-F_ = 9.9 Hz), 128.4 (CH), 128.6 (CH), 129.2 (CH), 129.3 (CH), 129.5 (CH), 129.6 (CH), 130.3 (Cq, d, *J*_C-F_ = 32.5 Hz), 130.9 (CH), 133.3 (Cq), 134.6 (CH), 136.2 (Cq), 136.3 (Cq), 136.5 (Cq), 137.2 (Cq), 138.0 (Cq), 138.2 (Cq), 143.6 (Cq), 144.4 (Cq); ^19^F-NMR (376 MHz, CDCl_3_) δ −65.8 (s, 3F); HRMS (ESI) *m/z* calcd for C_31_H_27_F_3_N_2_O_4_S_2_ [M]^+^ 612.1364, found 612.1370.

### 3.16. (E)-1,4-Ditosyl-2-(4-(trifluoromethyl)benzylidene)-2,3,4,5-tetrahydro-1H-benzo[e][1,4]diazepine [***(*E*)-3e***]

By following the same procedure described with respect to **3a**, 1,4-benzodiazepine **(*E*)-3e** was prepared from **1a** and **2e**, with a yield of 38% (16.3 mg, 26.6 µmol), as colorless quadrilaterals (AcOEt, mp. 186–191 °C). IR (ATR): 3037, 1598, 1490 cm^−1^; ^1^H-NMR (500 MHz, CDCl_3_) δ 2.38 (3H, s), 2.44 (3H, s), 4.18 (4H, m), 6.75 (1H, s), 7.12 (2H, d, *J* = 8.5 Hz), 7.25–7.37 (10H, m), 7.62 (2H, d, *J* = 8.5 Hz), 7.66 (2H, d, *J* = 8.0 Hz); ^13^C-NMR (125 MHz, CDCl_3_) δ 21.6 (CH_3_), 21.8 (CH_3_), 49.7 (CH_2_), 51.9 (CH_2_), 125.2 (Cq, q, *J*_C-F_ = 272.5 Hz), 125.7 (CH, d, *J*_C-F_ = 3.0 Hz), 127.4 (CH), 127.8 (CH), 128.2 (CH), 128.7 (CH), 129.5 (CH), 129.5 (CH), 129.7 (CH), 129.9 (CH), 130.3 (Cq, q, *J*_C-F_ = 32.7 Hz), 130.5 (CH), 135.1 (Cq), 135.2 (Cq), 135.4 (Cq), 135.7 (Cq), 137.5 (CH), 139.6 (Cq), 143.7 (Cq), 144.4 (Cq); ^19^F-NMR (376 MHz, CDCl_3_) δ −65.9 (s, 3F); HRMS (ESI) *m/z* calcd for C_31_H_27_F_3_N_2_O_4_S_2_ [M]^+^ 612.1364, found 612.1364.

### 3.17. N-(1,3-Diphenylprop-2-yn-1-yl)-4-methyl-N-(2-(((4-methylphenyl)sulfonamido)methyl)phenyl)benzenesulfonamide(***6***)

To a stirred solution of propargylic carbonate **2g** (25.3 mg, 95.2 µmol) in dioxane (0.5 mL) were added *N*-tosyl-disubstituted 2-aminobenzylamine **1a** (31.5 mg, 73.2 µmol) and Pd(PPh_3_)_4_ (8.4 mg, 7.3 µmol) at 50 °C. The reaction mixture was stirred for 1 h at the same temperature. After filtration through a small amount of silica gel and concentration under reduced pressure, the crude residue was purified by column chromatography on silica gel (hexane/AcOEt = 4:1 *v*/*v*) to afford **6**. Yield: 97% (44.1 mg, 71.0 µmol); colorless quadrilaterals (AcOEt, mp. 173–176 °C); IR (ATR): 3292, 3027, 1598, 1491 cm^−1^; ^1^H-NMR (500 MHz, CDCl_3_) δ 2.41 (3H, s), 2.53 (3H, s), 3.23 (1H, dd, *J* = 13.0, 9.0 Hz), 3.32 (1H, dd, *J* = 13.0, 4.5 Hz), 4.70 (1H, dd, *J* = 9.0, 4.5 Hz), 6.51 (1H, s), 6.94 (2H, d, *J* = 7.5 Hz), 7.03–7.15 (6H, m), 7.22–7.37 (8H, m), 7.42 (2H, d, *J* = 8.0 Hz), 7.68 (2H, d, *J* = 8.5 Hz), 7.73 (2H, d, *J* = 8.5 Hz); ^13^C-NMR (125 MHz, CDCl_3_) δ 21.7 (CH_3_), 21.8 (CH_3_), 42.5 (CH_2_), 56.3 (CH), 85.2 (Cq), 88.5 (Cq), 122.0 (CH), 127.5 (CH), 127.8 (CH), 128.5 (CH), 128.5 (CH), 128.6 (CH), 128.9 (CH), 129.0 (CH), 129.5 (CH), 129.7 (CH), 129.8 (CH), 130.5 (CH), 131.4 (CH), 131.6 (CH), 133.8 (Cq), 135.6 (Cq), 135.7 (Cq), 136.9 (Cq), 139.6 (Cq), 143.3 (Cq), 144.2 (Cq); HRMS (ESI) *m/z* calcd for C_36_H_32_N_2_O_4_S_2_ [M]^+^ 620.1803, found 556.2766 [M−SO_2_]^+^. For single-crystal data, see Supplementary Materials.

### 3.18. (Z)-3-Benzylidene-2-phenyl-1,4-ditosyl-2,3,4,5-tetrahydro-1H-benzo[e][1,4]diazepine [***(*Z*)-7g***]

To a stirred solution of propargylic carbonate **2g** (25.5 mg, 95.6 µmol) in dioxane (0.5 mL) were added *N*-tosyl-disubstituted 2-aminobenzylamine **1a** (31.6 mg, 73.4 µmol), Pd_2_(dba)_3_·CHCl_3_ (3.8 mg, 3.7 µmol), and DPPP (6.1 mg, 14.7 µmol) at 50 °C. The reaction mixture was stirred for 1 h at the same temperature. After filtration through a small amount of silica gel and concentration under reduced pressure, the crude residue was purified by column chromatography on silica gel (hexane/AcOEt = 4:1 *v*/*v*) to afford **(*Z*)-7g**. Yield: 99% (44.9 mg, 72.3 µmol); colorless quadrilaterals (AcOEt, mp. 183–187 °C); IR (ATR): 3023, 1598, 1492 cm^−1^; ^1^H-NMR (500 MHz, CDCl_3_) δ 2.37 (6H, m), 4.23 (1H, d, *J* = 11.5 Hz), 4.39 (1H, d, *J* = 11.5 Hz), 5.79 (1H,s), 6.02 (1H, s), 6.90 (2H, d, *J* = 7.5 Hz), 7.01–7.09 (5H, m), 7.20 (4H, d, *J* = 8.5 Hz), 7.28 (1H, m), 7.34–7.40 (7H, m), 7.46 (1H, d, *J* = 7.5 Hz), 7.59 (2H, d, *J* = 8.0 Hz); ^13^C-NMR (125 MHz, CDCl_3_) δ 21.7 (CH_3_), 21.7 (CH_3_), 50.5 (CH_2_), 68.0 (CH), 127.6 (CH), 127.9 (CH), 128.0 (CH), 128.1 (CH), 128.1 (CH), 128.7 (CH), 128.8 (CH), 129.0 (CH), 129.4 (CH), 129.7 (CH), 129.9 (CH), 130.4 (CH), 131.2 (CH), 132.5 (Cq), 133.9 (Cq), 134.3 (Cq), 135.6 (Cq), 137.3 (Cq), 138.8 (Cq), 139.5 (CH), 140.5 (Cq), 143.7 (Cq), 144.0 (Cq); HRMS (ESI) *m/z* calcd for C_36_H_32_N_2_O_4_S_2_ [M]^+^ 620.1803, found 556.2747 [M−SO_2_]^+^. For single-crystal data, see Supplementary Materials.

### 3.19. (Z)-2-(4-Methoxyphenyl)-1,4-ditosyl-3-(2,4,6-trifluorobenzylidene)-2,3,4,5-tetrahydro-1H-benzo[e][1,4]diazepine [***(*Z*)-7h***]

By following the same procedure described with respect to **(*Z*)-7g**, 1,4-benzodiazepine **(*Z*)-7h** was prepared from **1a** and **2h**, with a yield of 71% (29.3 mg, 41.6 µmol), as colorless quadrilaterals (AcOEt, mp. 196–201 °C). IR (ATR): 2925, 1596, 1495 cm^−1^; ^1^H-NMR (500 MHz, CDCl_3_) δ 2.36 (3H, s), 2.47 (3H, s), 3.84 (3H, s), 4.26 (1H, d, *J* = 11.5 Hz), 4.43 (1H, d, *J* = 11.5 Hz), 5.69 (1H, s), 5.92 (1H, d, *J* = 2.0 Hz), 6.42 (2H, t, *J* = 8.0 Hz), 6.71 (2H, s), 6.89 (2H, d, *J* = 8.5 Hz), 7.05 (2H, d, *J* = 8.5 Hz), 7.14–7.17 (4H, m), 7.32–7.36 (4H, m), 7.69 (2H, d, *J* = 8.5 Hz); ^13^C-NMR (125 MHz, CDCl_3_) δ 21.7 (CH_3_), 21.8 (CH_3_), 50.5 (CH_2_), 55.5 (CH_3_), 67.3 (CH), 100.1 (CH, dt, *J*_C-F_ = 3.0, 25.8 Hz), 109.6 (Cq, dt, *J*_C-F_ = 4.0, 19.8 Hz), 114.3 (CH), 116.3 (CH), 126.5 (CH), 127.6 (CH), 127.9 (CH), 128.7 (CH), 129.4 (CH), 129.4 (CH), 130.0 (CH), 130.5 (CH), 131.6 (CH), 132.1 (Cq), 134.1 (Cq), 136.0 (Cq), 137.4 (Cq), 138.1 (Cq), 138.5 (Cq), 143.6 (Cq), 144.2 (Cq), 149.6 (Cq), 159.6 (Cq), 160.3 (Cq, dt, *J*_C-F_ = 14.9, 244.7 Hz); ^19^F-NMR (376 MHz, CDCl_3_) δ −108.5 (s, 2F) −111.6–111.7 (m, 1F); HRMS (ESI) *m/z* calcd for C_37_H_31_F_3_N_2_O_5_S_2_ [M]^+^ 704.1626, found 640.2001 [M−SO_2_]^+^.

### 3.20. (Z)-2-Phenyl-1,4-Ditosyl-3-(2,4,6-trifluorobenzylidene)-2,3,4,5-tetrahydro-1H-benzo[e][1,4]diazepine [***(*Z*)-7i***]

By following the same procedure described with respect to **(*Z*)-7g**, 1,4-benzodiazepine **(*Z*)-7h** was prepared from **1a** and **2i**, with a yield of 85% (45.2 mg, 66.9 µmol), as colorless quadrilaterals (AcOEt, mp. 182–186 °C). IR (ATR): cm^−1^; ^1^H-NMR (500 MHz, CDCl_3_) ^1^H-NMR (500 MHz, CDCl_3_) δ 2.36 (3H, s), 2.47 (3H, s), 4.25 (1H, d *J* = 11.5 Hz), 4.45 (1H, d, *J* = 11.5 Hz), 6.41 (2H, t, *J* = 8.0 Hz), 7.05 (2H, d, *J* = 8.0 Hz), 7.27 (1H, s), 7.31–7.37 (9H, m), 7.70 (2H, d, *J* = 8.0 Hz); ^13^C-NMR (125 MHz, CDCl_3_) δ 21.7 (CH_3_), 21.8 (CH_3_), 50.4 (CH_2_), 67.7 (CH), 100.1 (CH, d, *J*_C-F_ = 25.8 Hz), 109.5 (Cq, dt, *J*_C-F_ *=* 5.0, 18.8 Hz), 126.8 (CH), 127.6 (CH), 127.9 (CH), 128.2 (Cq), 128.4 (CH), 128.7 (CH), 128.9 (CH), 129.4 (CH), 129.5 (CH), 130.0 (CH), 130.5 (CH), 131.7 (CH), 134.1 (Cq), 136.0 (Cq), 137.3 (Cq), 138.2 (Cq), 138.3 (Cq), 140.0 (Cq), 143.7 (Cq), 144.3 (Cq), 160.3 (Cq, ddd, *J*_C-F_ = 10.0, 14.9, 254.6 Hz), 162.3 (Cq, dt, *J*_C-F_ = 15.9, 249.7 Hz); ^19^F-NMR (376 MHz, CDCl_3_) δ −108.4 (s, 2F) −111.5–111.6 (m, 1F); HRMS (ESI) *m/z* calcd for C_36_H_29_F_3_N_2_O_4_S_2_ [M]^+^ 674.1521, found 674.1534. For single-crystal data, see Supplementary Materials.

### 3.21. 2-Methylene-5-phenyl-1,4-ditosyl-2,3,4,5-tetrahydro-1H-benzo[e][1,4]diazepine (***3j***)

To a stirred solution of propargylic carbonate **2j** (24.6 mg, 129 µmol) in dioxane (0.5 mL) were added *N*-tosyl-disubstituted 2-aminobenzylamine **1b** (50.4 mg, 99.5 µmol) and Pd(PPh_3_)_4_ (11.6 mg, 10.0 µmol) at 80 °C. The reaction mixture was stirred for 1 h at the same temperature. After filtration through a small amount of silica gel and concentration under reduced pressure, the crude residue was purified by column chromatography on silica gel (hexane/AcOEt = 5:1 *v*/*v*) to produce the 1,4-benzodiazepines **3j**. Yield: 62% (33.6 mg, 61.7 µmol); colorless needles (CH_2_CI_2_, mp. 186–190 °C); IR (ATR): 3062, 1645, 1595, 1491 cm^−1^; ^1^H-NMR (500 MHz, CDCl_3_) δ 2.30 (3H, s), 2.34 (3H, s), 3.25 (1H, d, *J* = 15.0 Hz), 4.13 (1H, d, *J* = 15.0 Hz), 4.92 (1H, s), 5.31 (1H, s), 6.38 (1H, s), 6.98–7.02 (4H, m), 7.05–7.07 (2H, m), 7.10–7.12 (2H, m), 7.20–7.25 (2H, m), 7.36–7.39 (6H, m), 7.63 (1H, d, *J* = 8.0 Hz); ^13^C-NMR (125 MHz, CDCl_3_) δ 21.6 (CH_3_), 21.7 (CH_3_), 47.9 (CH_2_), 64.2 (CH), 119.5 (CH), 127.0 (CH), 127.2 (CH), 127.5 (CH), 127.9 (CH), 129.0 (CH), 129.1 (CH), 129.3 (CH), 129.3 (CH), 129.4 (CH), 132.2 (Cq), 133.8 (Cq), 135.0 (Cq), 138.0 (Cq), 138.9 (Cq), 139.6 (Cq), 143.4 (Cq), 144.2 (Cq); HRMS (ESI) *m/z* calcd for C_30_H_28_N_2_O_4_S_2_ [M]^+^ 544.1490, found 544.1490.

## 4. Conclusions

In summary, we developed an efficient palladium-catalyzed reaction for the synthesis of substituted 1,4-benzodiazepines via the cyclization of *N*-tosyl-disubstituted 2-aminobenzylamines with propargylic carbonates. The reaction proceeds through π-allylpalladium intermediates, enabling intramolecular nucleophilic attack to furnish seven-membered heterocycles. Notably, stereoselectivity and nearly quantitative yields were achieved across a variety of propargylic substrates. Moreover, regioselectivity was found to be governed by both steric and electronic effects, particularly in cases involving unsymmetrical diaryl-substituted carbonates. The utility of this reaction was further demonstrated by constructing a benzodiazepine framework resembling those found in clinically relevant drugs. These findings provide a versatile synthetic platform for the preparation of structurally diverse benzodiazepine derivatives with potential medicinal applications.

## Figures and Tables

**Table 1:** Initial attempts using **1a** with phenyl-substituted propargylic carbonate **2a**.

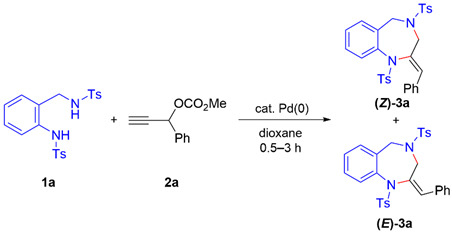				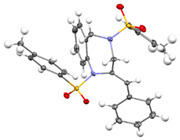
				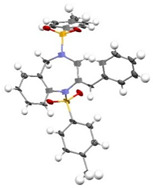
**Entry**	**Catalyst**	**Temp. (°C)**	**(*Z*)-3a:(*E*)-3a**	**Total Yields (%)**
1 ^a^	Pd_2_(dba)_3_·CHCl_3_, DPPM	50	3:1	21
2 ^a^	Pd_2_(dba)_3_·CHCl_3_, DPPE	50	3:1	30
3 ^a^	Pd_2_(dba)_3_·CHCl_3_, DPPP	50	2:1	43
4 ^a^	Pd_2_(dba)_3_·CHCl_3_, DPPB	50	3:1	22
5 ^a^	Pd_2_(dba)_3_·CHCl_3_, DPPPent	50	2:1	51
6 ^b^	Pd(PPh_3_)_4_	50	2:1	98
7 ^b^	Pd(PPh_3_)_4_	110	2:1	77
8 ^b^	Pd(PPh_3_)_4_	80	2:1	99
9 ^b^	Pd(PPh_3_)_4_	25	3:1	99

^a^ 5 mol% for Pd and 20 mol% for ligand were used. ^b^ 10 mol% for Pd was used.

**Table 2:** Attempts using **1a** with diphenyl-substituted propargylic carbonate **2g**.

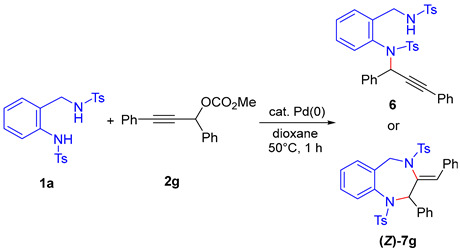			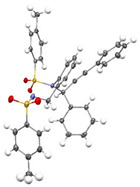	
			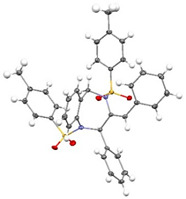	
**Entry**	**Catalyst**	**Product**		**Yield %**
1 ^a^	Pd(PPh_3_)_4_	**6**		97
2 ^b^	Pd_2_(dba)_3_·CHCl_3_, DPPM	**(*Z*)-7g**		45
3 ^b^	Pd_2_(dba)_3_·CHCl_3_, DPPE	**(*Z*)-7g**		76
4 ^b^	Pd_2_(dba)_3_·CHCl_3_, DPPP	**(*Z*)-7g**		99

^a^ 10 mol% for Pd was used. ^b^ 5 mol% for Pd and 20 mol% for ligand were used.

## Data Availability

The original contributions presented in the study are included in the article/Supplementary Materials. Further inquiries can be directed to the corresponding author.

## Supplementary Materials

The following supporting information can be downloaded at: https://www.mdpi.com/article/10.3390/molecules30143004/s1, Copies of ^1^H and ^13^C NMR Spectra for products. Single-crystal information [**(*Z*)-3a**, **(*E*)-3a**, **6**, **(*Z*)-7g** and **(*Z*)-7i**].
