# Supplementary material for: Synthesis of Substituted 1,4-Benzodiazepines by Palladium-Catalyzed Cyclization of N-Tosyl-Disubstituted 2-Aminobenzylamines with Propargylic Carbonates
Source: Molecules. 2025 Jul 17;30(14):3004. doi: 10.3390/molecules30143004 (PMC12299192; doi:10.3390/molecules30143004)

Supporting Information

for

Synthesis of Substituted 1,4-Benzodiazepines by Palladium-Catalyzed Cyclization of *N*-Tosyl-Disubstituted 2-Aminobenzylamines with Propargylic Carbonates

Masahiro Yoshida[1,*], Saya Okubo[1], Akira Kurosaka[1], Shunya Mori[1], Touya Kariya[1] and Kenji Matsumoto[2]

[1] Faculty of Pharmaceutical Sciences, Tokushima Bunri University, 180 Nishihamabouji, Yamashiro-cho, Tokushima, 770-8514, Japan.
E-mail: yoshida@ph.bunri-u.ac.jp
[2] Department of Engineering, Graduate School of Science and Engineering, Kagoshima University, 1-21-40 Korimoto, Kagoshima, 890-0065, Japan

Contents

1. Crystal structure determination by X-ray diffraction analysis

2. ^{1}H, ^{13}C NMR and ^{19}F NMR Spectra of Compounds

1. Crystal structure determination by X-ray diffraction analysis

A single crystal was mounted on a glass fiber and analyzed at −180 °C (Bruker Apex III Ultra diffractometer, Mo/Kα radiation). Data correction and reduction were performed using the Apex III crystallographic package. The structure was solved by direct methods using SHELXS-97 and refined by means of a full-matrix least- squares technique based on F2 using SHELXL-2014/7. All non-hydrogen atoms were refined anisotropically, and all hydrogen atoms were positioned geometrically.

Compound (*Z*)-3a (CCDC 2454097)

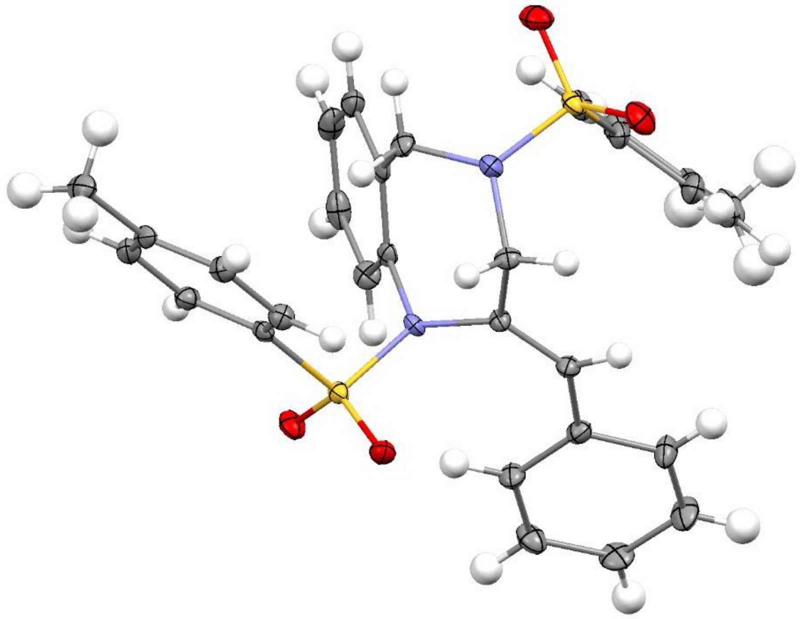

Although the crystal quality was insufficient to obtain high-resolution data, the available data were adequate to allow for a reliable determination of the molecular structure.

A total of 720 frames were collected. The total exposure time was 2.00 hours. The frames were integrated with the Bruker SAINT software package using a narrow-frame algorithm. The integration of the data using a triclinic unit cell yielded a total of 8939 reflections to a maximum θ angle of 29.07° (0.73 Å resolution), of which 6776 were independent (average redundancy 1.319, completeness = 87.2%, R_{int} = 0.99%, R_{sig} = 1.69%) and 6251 (92.25%) were greater than 2σ(F^2). The final cell constants of a = 11.2060 Å, b = 12.3310 Å, c = 12.3450 Å, α = 60.380°, β = 78.490°, γ = 85.950°, volume = 1452.3 $Å^3$, are based upon the refinement of the XYZ-centroids of 6419 reflections above 20 σ(I) with 4.835° < 2θ < 58.27°. Data were corrected for absorption effects using the multi-scan method (SADABS). The ratio of minimum to maximum apparent transmission was 0.934. The structure was solved and refined using the Bruker SHELXTL Software Package, using the space group P -1, with Z = 2 for the formula unit, $C_{30}H_{28}N_2O_4S_2$. The final anisotropic full-matrix least-squares refinement on F^2 with 346 variables converged at R1 = 12.83% for the observed data and wR2 = 46.79% for all data. The goodness-of-fit was 2.425. The largest peak in the final difference electron density synthesis was 6.855 $e^-/Å^3$ and the largest hole was -0.539 $e^-/Å^3$ with an RMS deviation of 0.267 $e^-/Å^3$. On the basis of the final model, the calculated density was 1.245 g/cm^3 and F(000), 572 e^-.

Compound (*E*)-3a (CCDC 2455059)

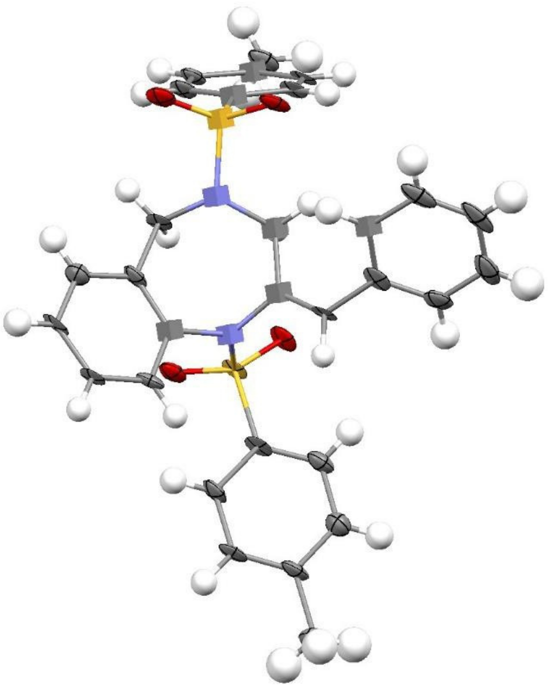

Although the crystal quality was insufficient to obtain high-resolution data, the available data were adequate to allow for a reliable determination of the molecular structure.

A total of 720 frames were collected. The total exposure time was 4.00 hours. The frames were integrated with the Bruker SAINT software package using a narrow-frame algorithm. The integration of the data using a monoclinic unit cell yielded a total of 11,876 reflections to a maximum θ angle of 23.18° (0.90 Å resolution), of which 3660 were independent (average redundancy 3.245, completeness = 93.9%, R_{int} = 21.84%, R_{sig} = 16.26%) and 2773 (75.77%) were greater than $2\sigma(F^2)$. The final cell constants of a = 9.113(7) Å, b = 18.441(14) Å, c = 16.324(13) Å, β = 90.800(9)°, volume = 2743.(4) $Å^3$, are based upon the refinement of the XYZ-centroids of 5530 reflections above 20 σ(I) with 4.417° < 2θ < 45.61°. Data were corrected for absorption effects using the multi-scan method (SADABS). The ratio of minimum to maximum apparent transmission was 0.604. The calculated minimum and maximum transmission coefficients (based on crystal size) are 0.9190 and 0.9230.

The structure was solved and refined using the Bruker SHELXTL Software Package, using the space group P 1 21/n 1, with Z = 4 for the formula unit, $C_{30}H_{28}N_2O_4S_2$. The final anisotropic full-matrix least-squares refinement on F^2 with 345 variables converged at R1 = 13.62% for the observed data and wR2 = 35.42% for all data. The goodness-of-fit was 1.423. The largest peak in the final difference electron density synthesis was 1.029 $e^-/Å^3$ and the largest hole was -1.382 $e^-/Å^3$ with an RMS deviation of 0.212 $e^-/Å^3$. On the basis of the final model, the calculated density was 1.319 g/cm^3 and F(000), 1144 e^-.

Compound 6 (CCDC 2454077)

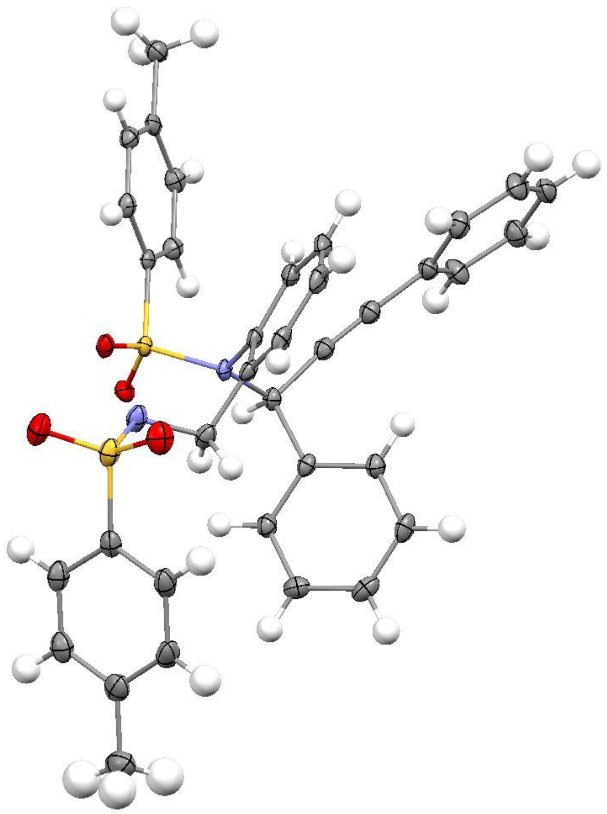

A total of 720 frames were collected. The total exposure time was 8.00 hours. The frames were integrated with the Bruker SAINT software package using a narrow-frame algorithm. The integration of the data using a triclinic unit cell yielded a total of 8592 reflections to a maximum θ angle of 27.11° (0.78 Å resolution), of which 6500 were independent (average redundancy 1.322, completeness = 96.4%, R_{int} = 3.22%, R_{sig} = 5.74%) and 5483 (84.35%) were greater than 2σ(F^2). The final cell constants of a = 8.8792(15) Å, b = 14.083(2) Å, c = 14.386(2) Å, α = 62.488(2)°, β = 78.927(2)°, γ = 73.393(2)°, volume = 1525.1(4) $Å^3$, are based upon the refinement of the XYZ-centroids of 5144 reflections above 20 σ(I) with 4.799° < 2θ < 54.22°.

The structure was solved and refined using the Bruker SHELXTL Software Package, using the space group P -1, with Z = 2 for the formula unit, $C_{36}H_{32}N_2O_4S_2$. The final anisotropic full-matrix least-squares refinement on F^2 with 399 variables converged at R1 = 5.24% for the observed data and wR2 = 16.14% for all data. The goodness-of-fit was 1.121.

Compound (*Z*)-7g (CCDC 2454078)

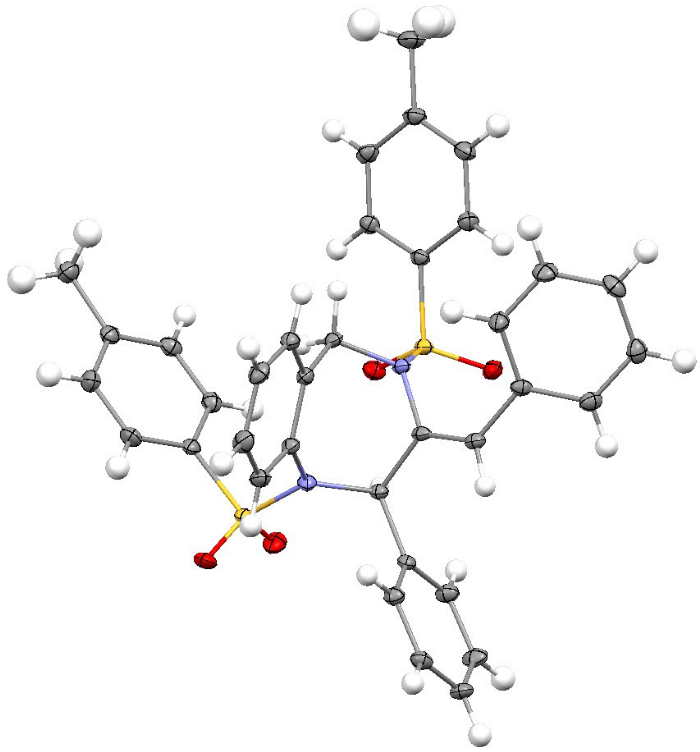

A total of 720 frames were collected. The total exposure time was 3.00 hours. The frames were integrated with the Bruker SAINT software package using a narrow-frame algorithm. The integration of the data using a monoclinic unit cell yielded a total of 18,809 reflections to a maximum θ angle of 29.26° (0.73 Å resolution), of which 7579 were independent (average redundancy 2.482, completeness = 91.2%, R_{int} = 3.25%, R_{sig} = 4.18%) and 5991 (79.05%) were greater than 2σ(F^2). The final cell constants of a = 11.4433(8) Å, b = 21.0623(14) Å, c = 12.8159(9) Å, β = 99.0880(10)°, volume = 3050.1(4) Å^3, are based upon the refinement of the XYZ-centroids of 5829 reflections above 20 σ(I) with 4.839° < 2θ < 58.00°. The structure was solved and refined using the Bruker SHELXTL Software Package, using the space group P 1 21/c 1, with Z = 4 for the formula unit, $C_{36}H_{32}N_2O_4S_2$. The final anisotropic full-matrix least-squares refinement on F^2 with 399 variables converged at R1 = 3.79% for the observed data and wR2 = 9.66% for all data. The goodness-of-fit was 1.012.

Compound (*Z*)-7i (CCDC 2454080)

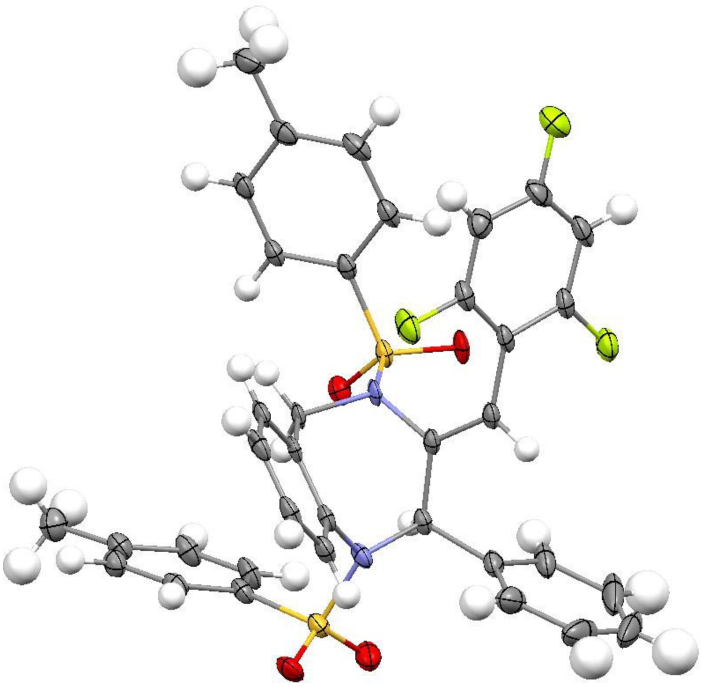

A total of 720 frames were collected. The total exposure time was 2.00 hours. The frames were integrated with the Bruker SAINT software package using a narrow-frame algorithm. The integration of the data using a triclinic unit cell yielded a total of 9306 reflections to a maximum θ angle of 27.59° (0.77 Å resolution), of which 7126 were independent (average redundancy 1.306, completeness = 96.0%, R_{int} = 1.97%, R_{sig} = 3.85%) and 6168 (86.56%) were greater than 2σ(F^2). The final cell constants of a = 10.0059(18) Å, b = 12.910(2) Å, c = 13.064(2) Å, α = 73.517(2) °, *β* = 89.965(2) °, *γ* = 82.330(2) °, volume = 1602.5(5) $Å^3$, are based upon the refinement of the XYZ-centroids of 5758 reflections above 20 σ(I) with 5.142° < 2 *θ* < 55.15° . The structure was solved and refined using the Bruker SHELXTL Software Package, using the space group P -1, with Z = 2 for the formula unit, $C_{36}H_{29}F_3N_2O_4S_2$. The final anisotropic full-matrix least-squares refinement on F^2 with 426 variables converged at R1 = 5.90% for the observed data and wR2 = 16.08% for all data. The goodness-of-fit was 0.989.

2. ^{1}H and ^{13}C NMR Spectra of Compounds

^{1}H-NMR (500 MHz, $CDCl_3$)

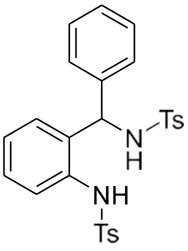

1b

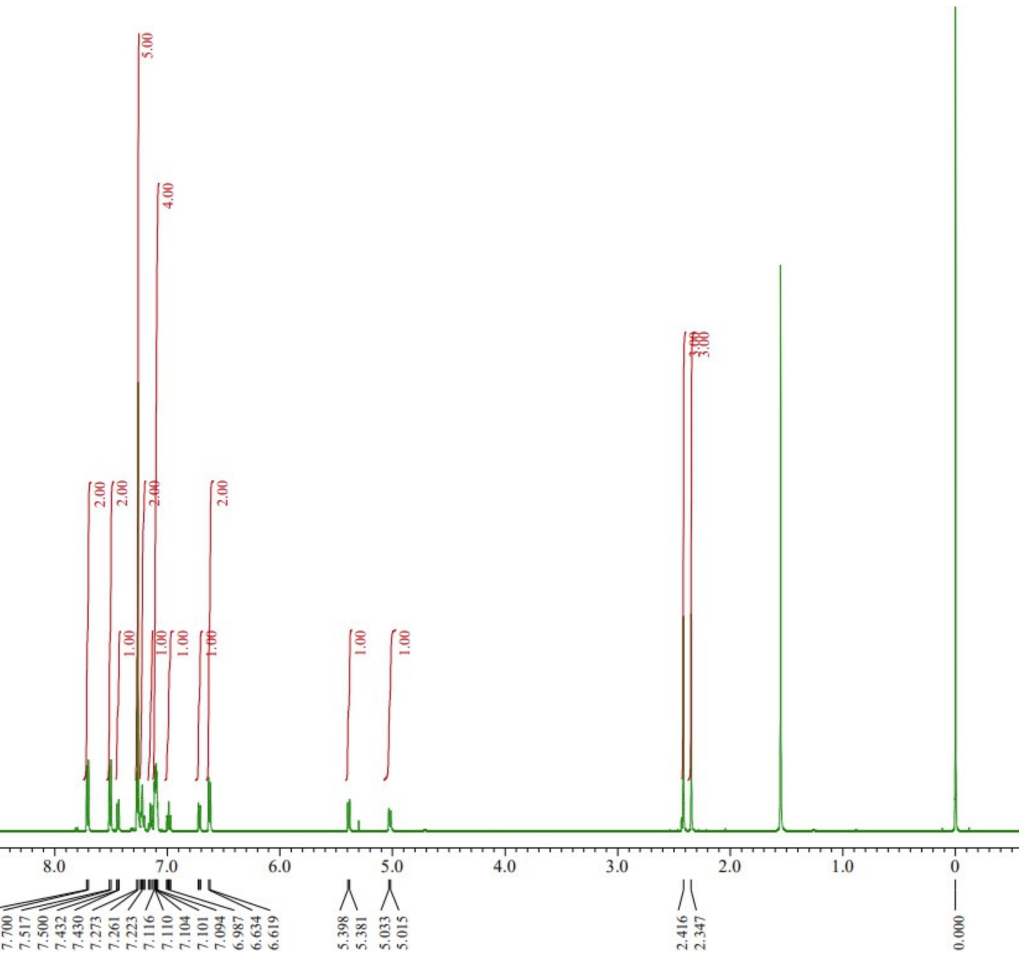

^{13}C-NMR (125 MHz, $CDCl_3$)

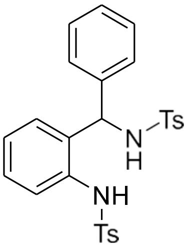

1b

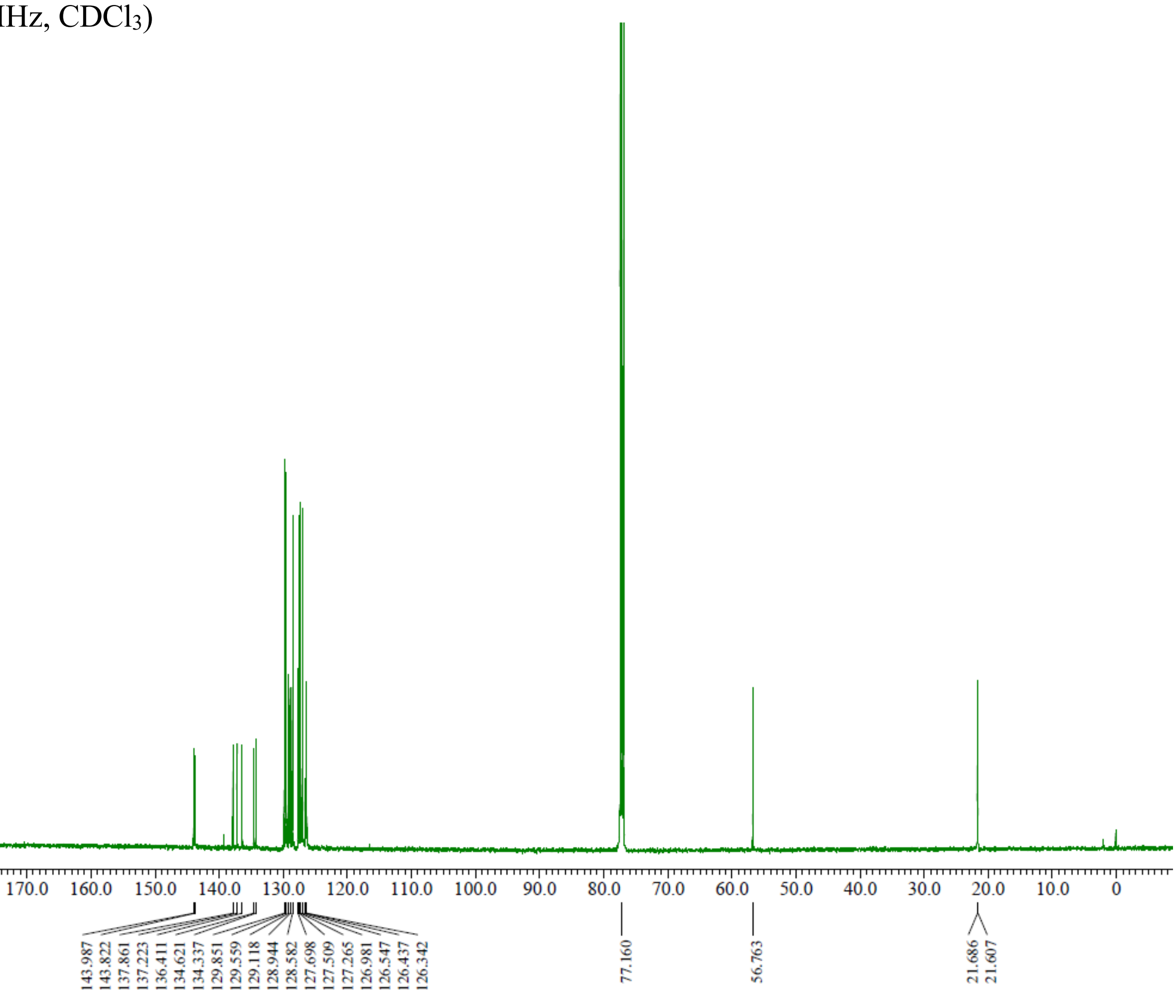

^{1}H-NMR (500 MHz, $CDCl_3$)

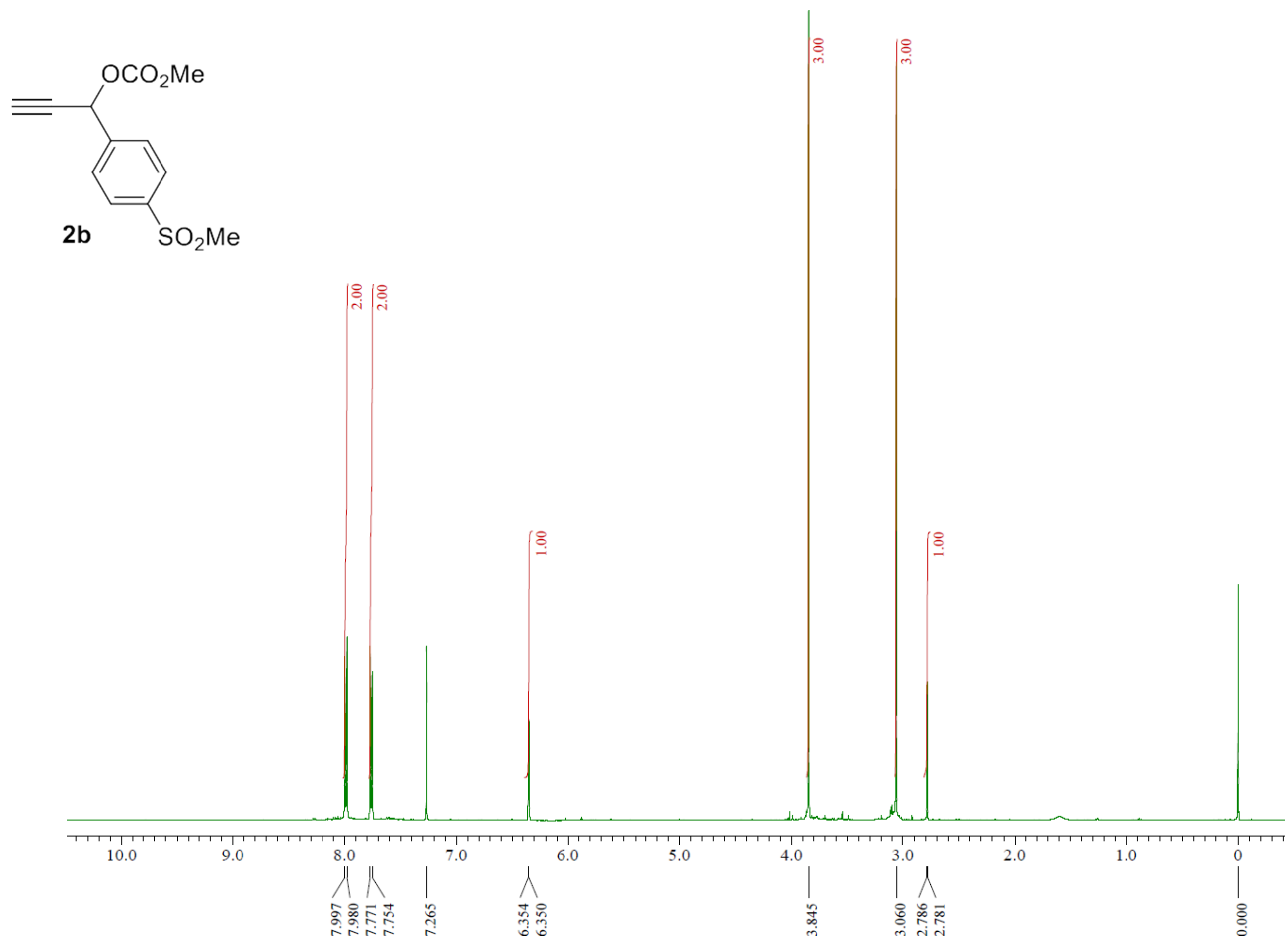

^{13}C-NMR (125 MHz, $CDCl_3$)

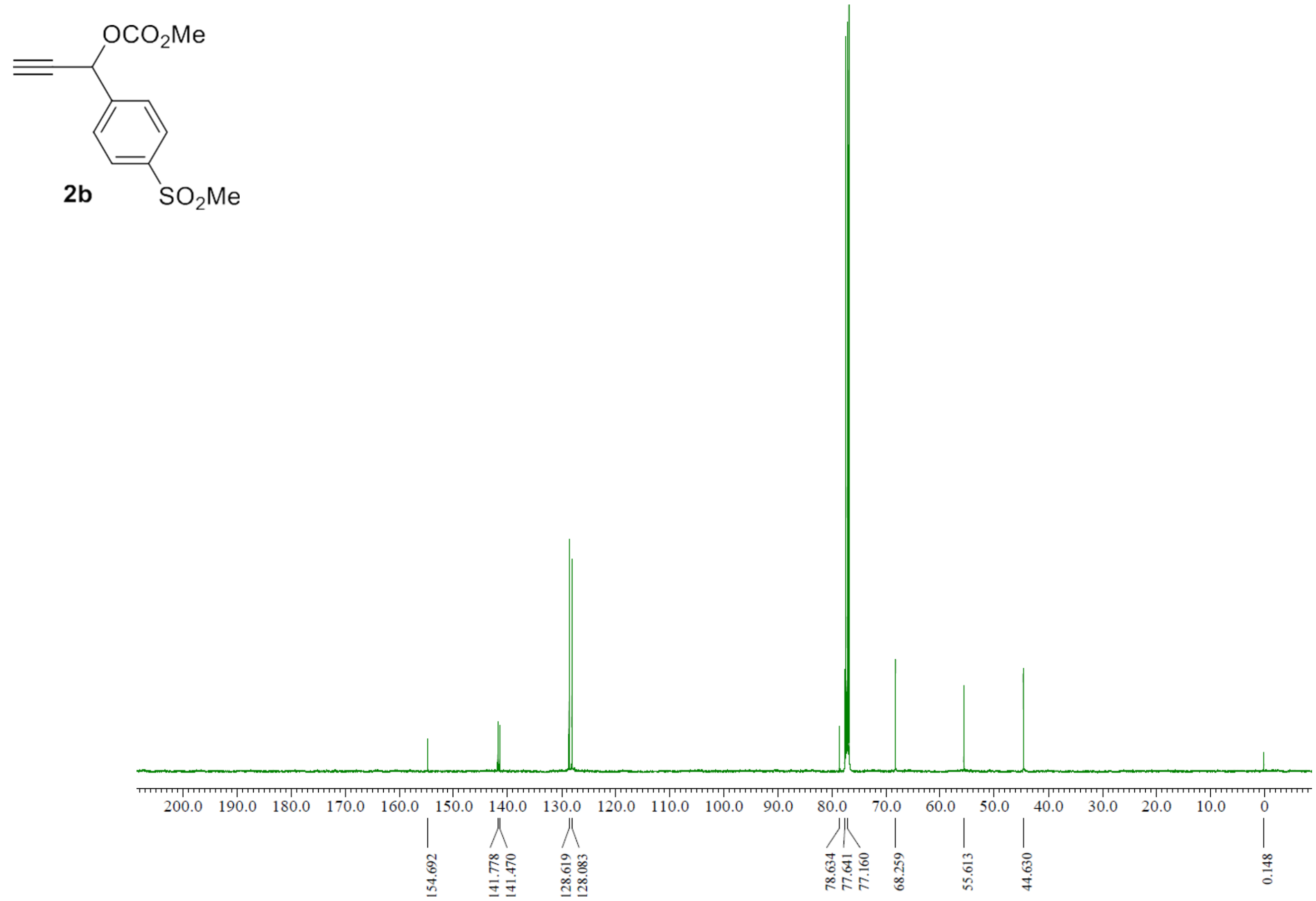

^{1}H-NMR (500 MHz, $CDCl_3$)

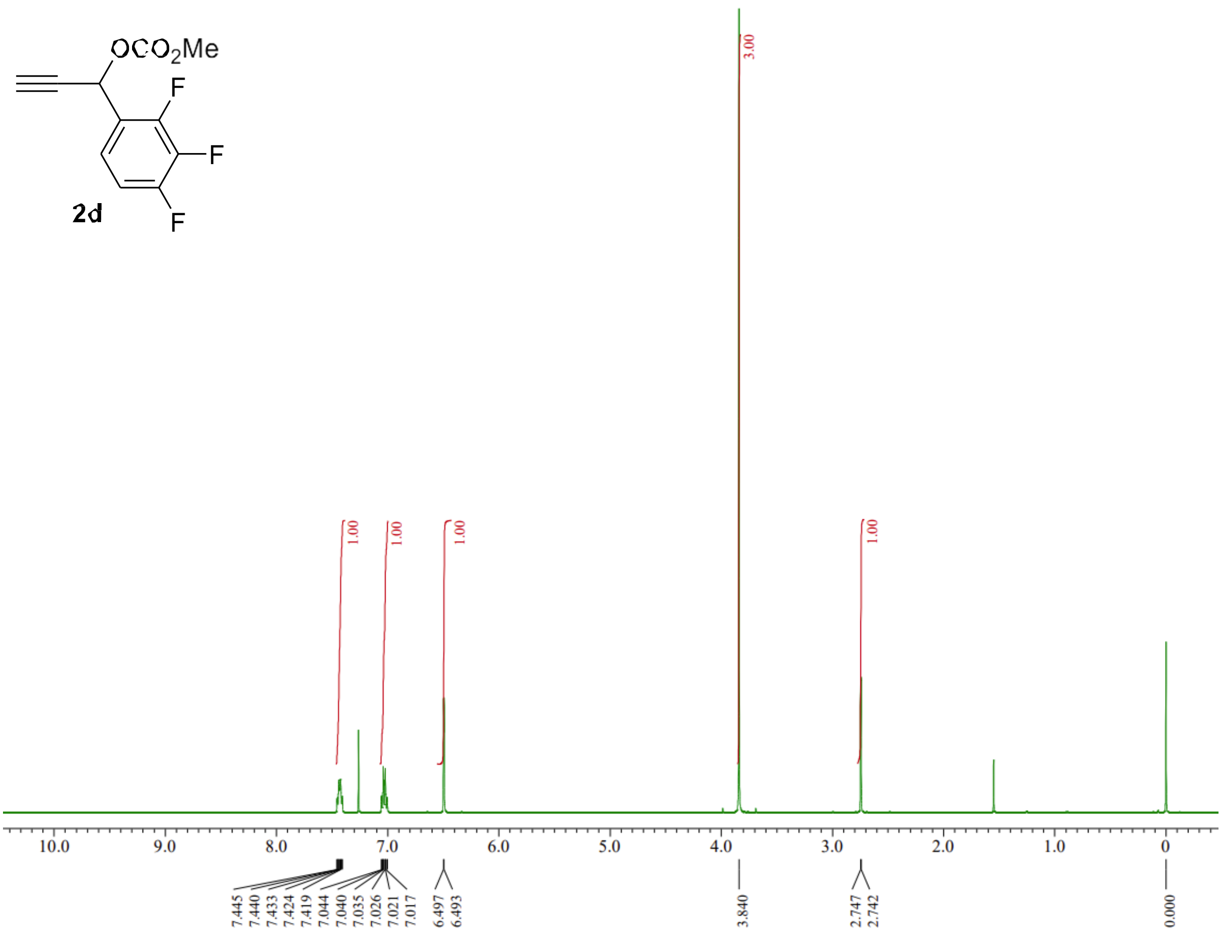

^{13}C-NMR (125 MHz, $CDCl_3$)

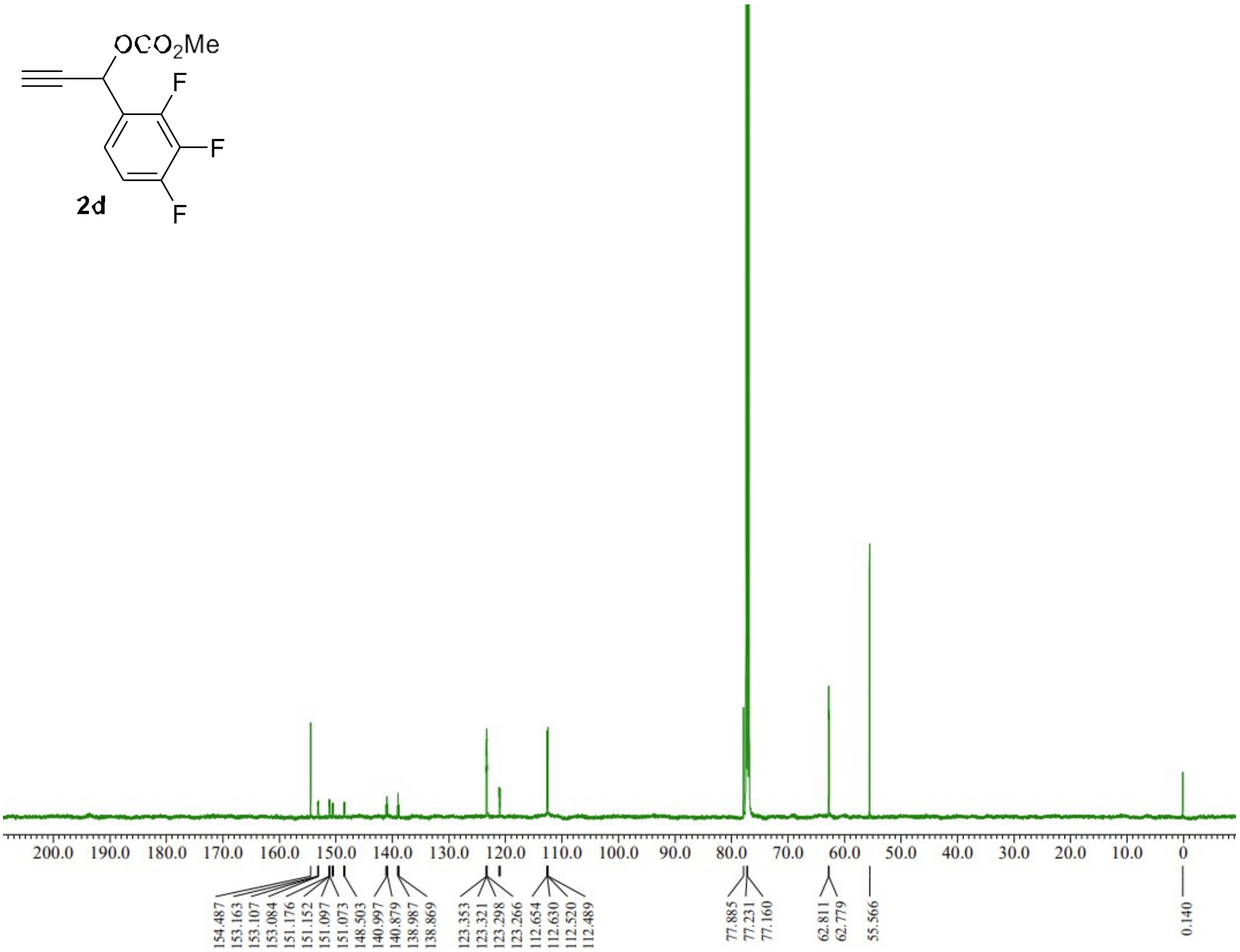

^{19}F-NMR (376 MHz, $CDCl_3$)

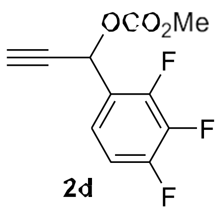

2d

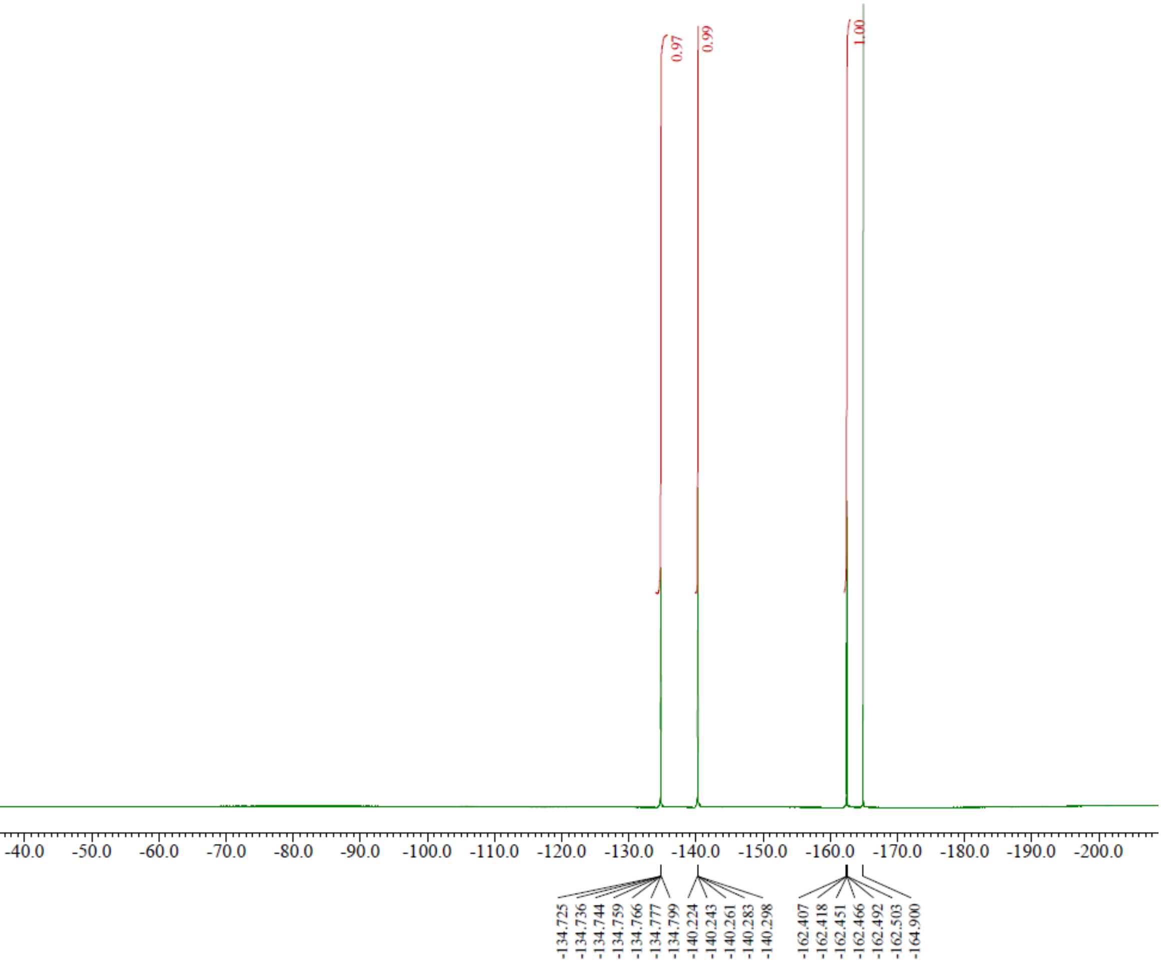

^{1}H-NMR (500 MHz, $CDCl_3$)

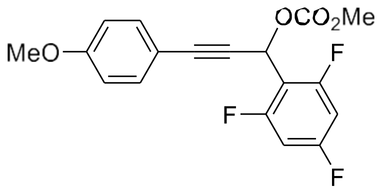

2h

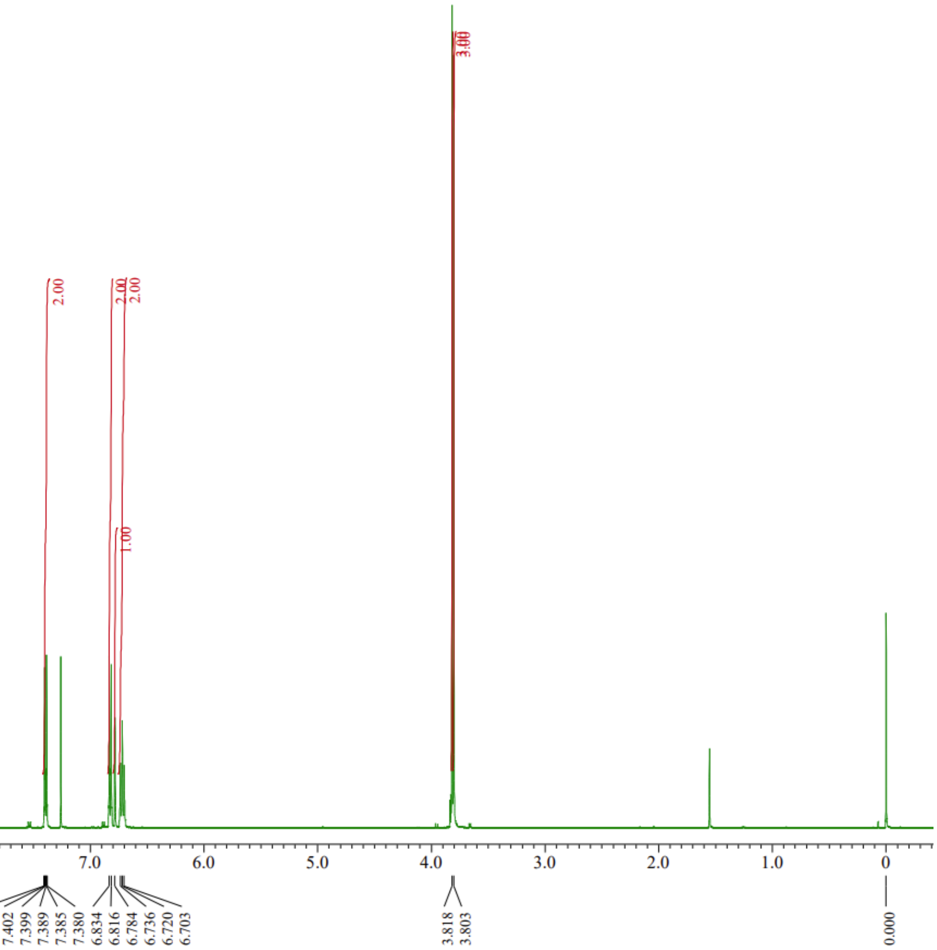

^{13}C-NMR (125 MHz, $CDCl_3$)

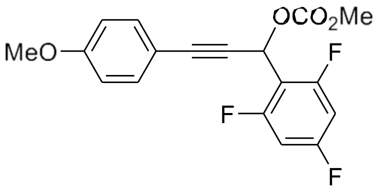

2h

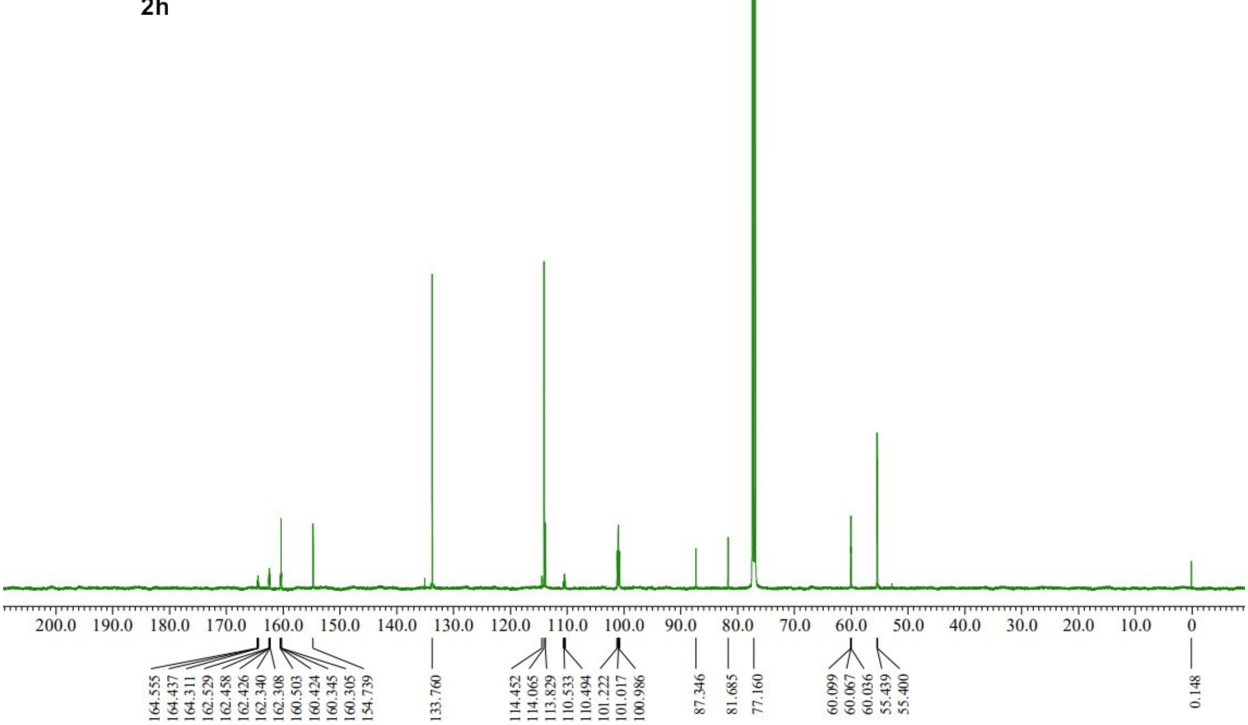

^{19}F-NMR (376 MHz, $CDCl_3$)

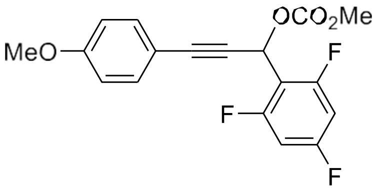

2h

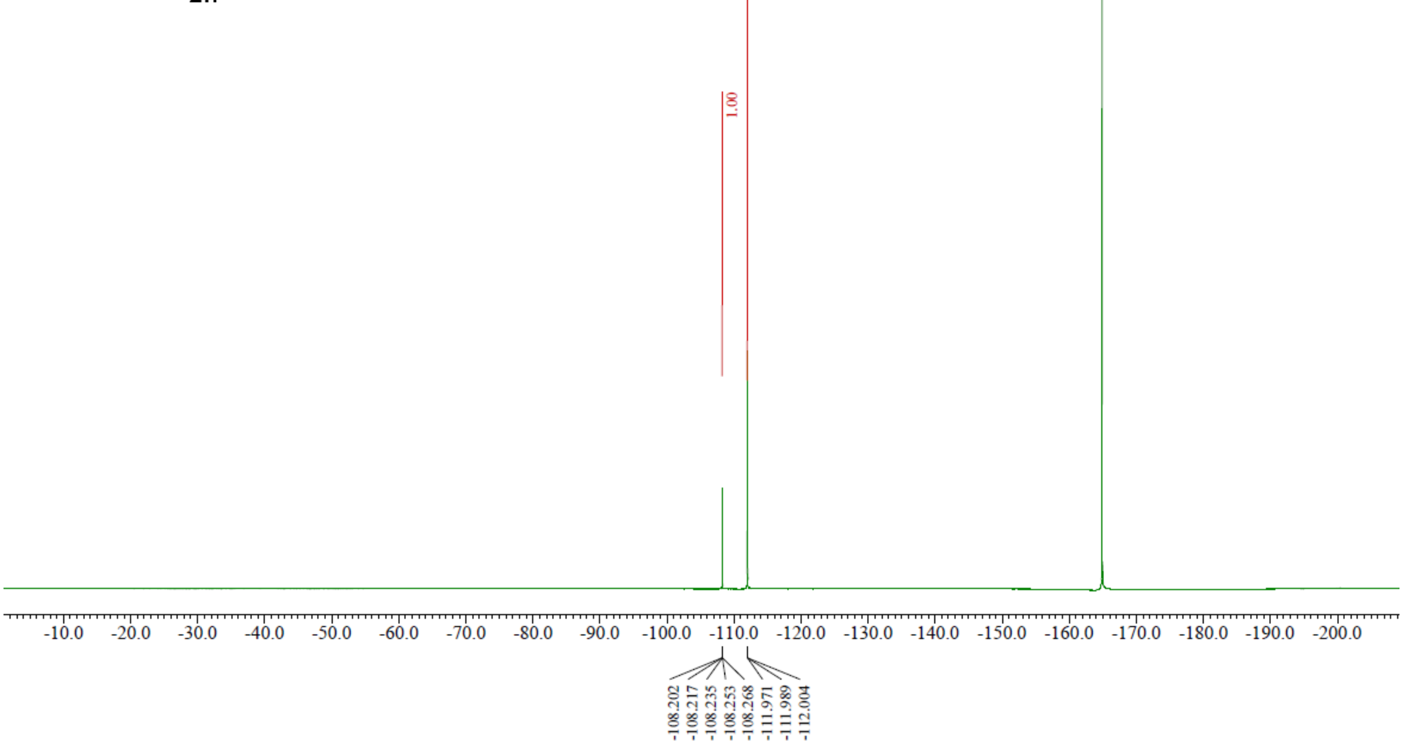

^{1}H-NMR (500 MHz, $CDCl_3$)

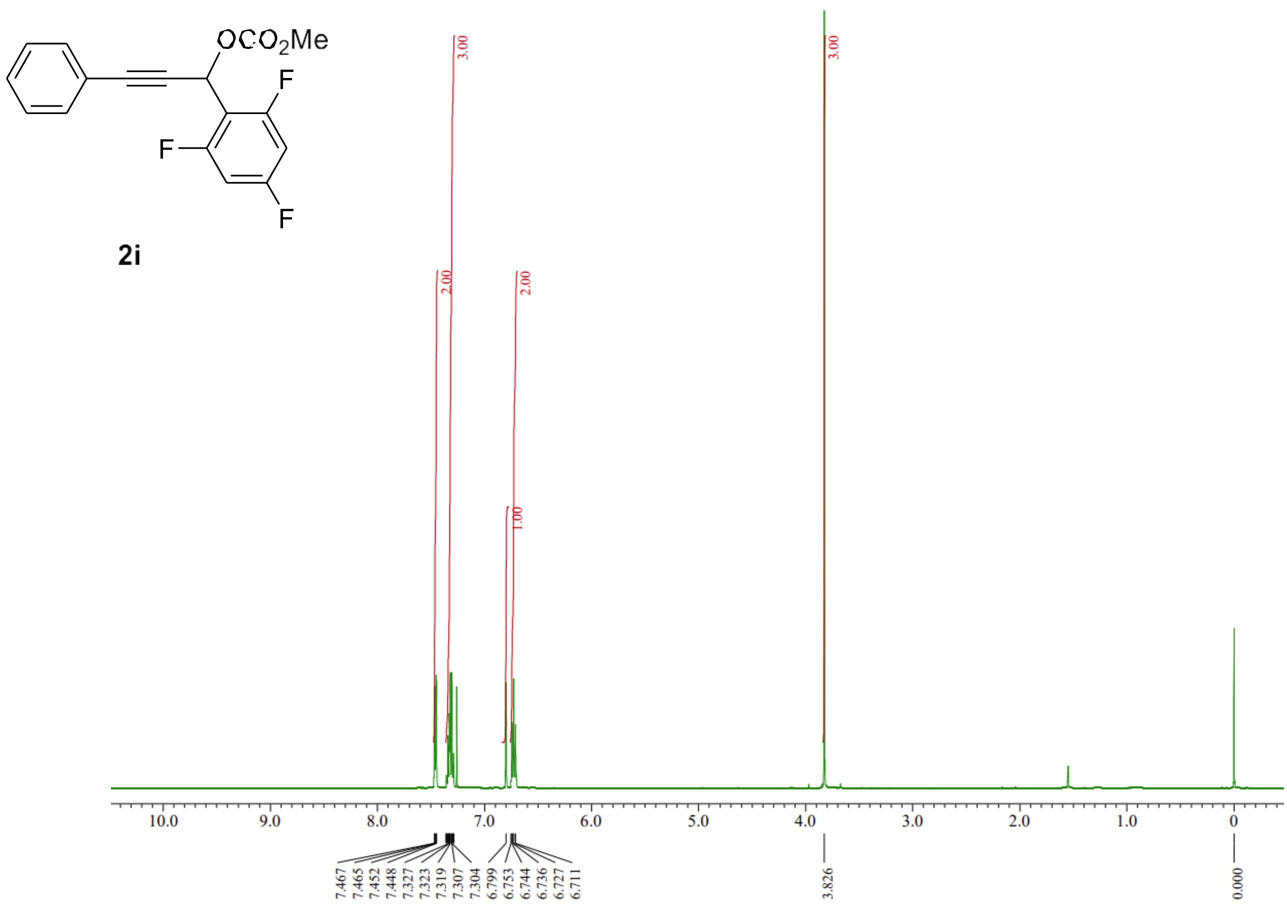

^{13}C-NMR (125 MHz, $CDCl_3$)

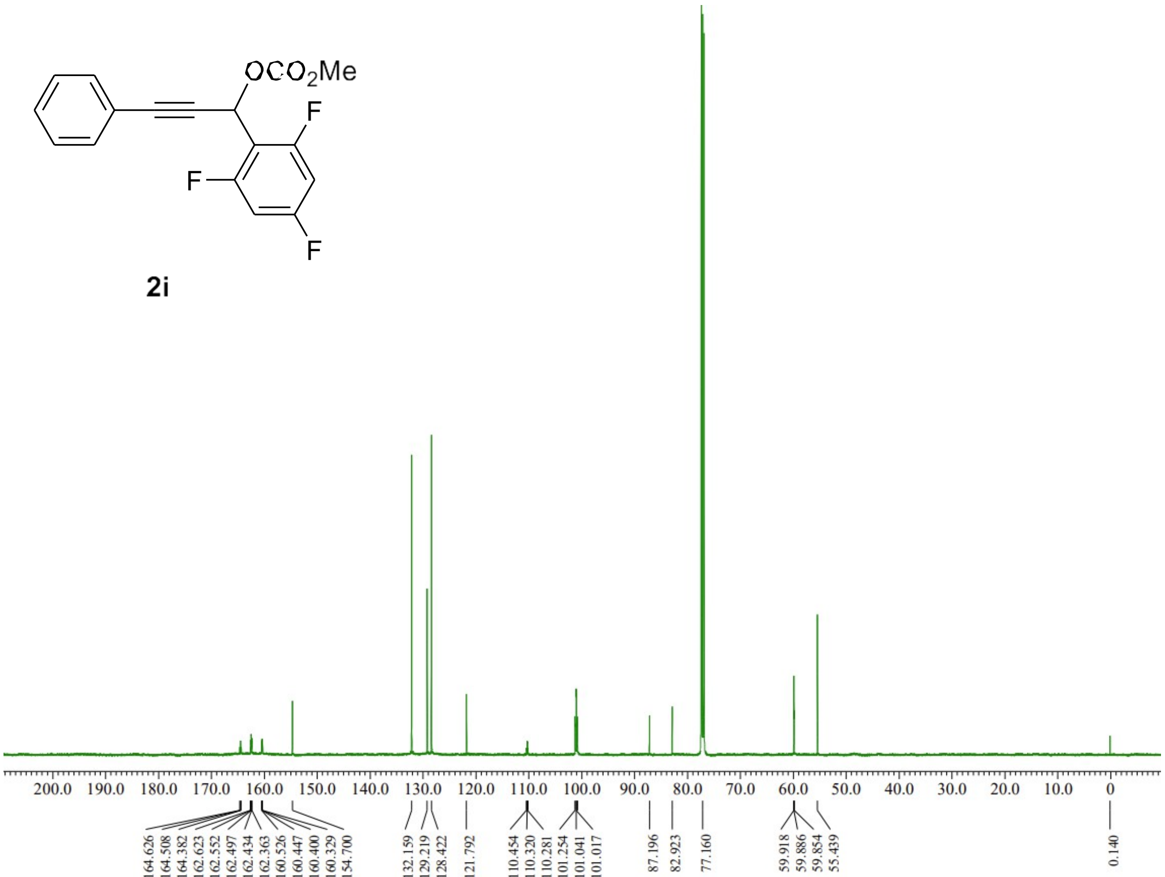

^{19}F-NMR (376 MHz, $CDCl_3$)

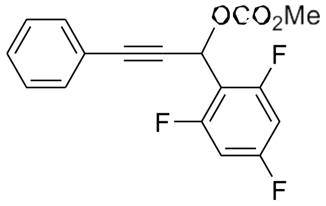

2i

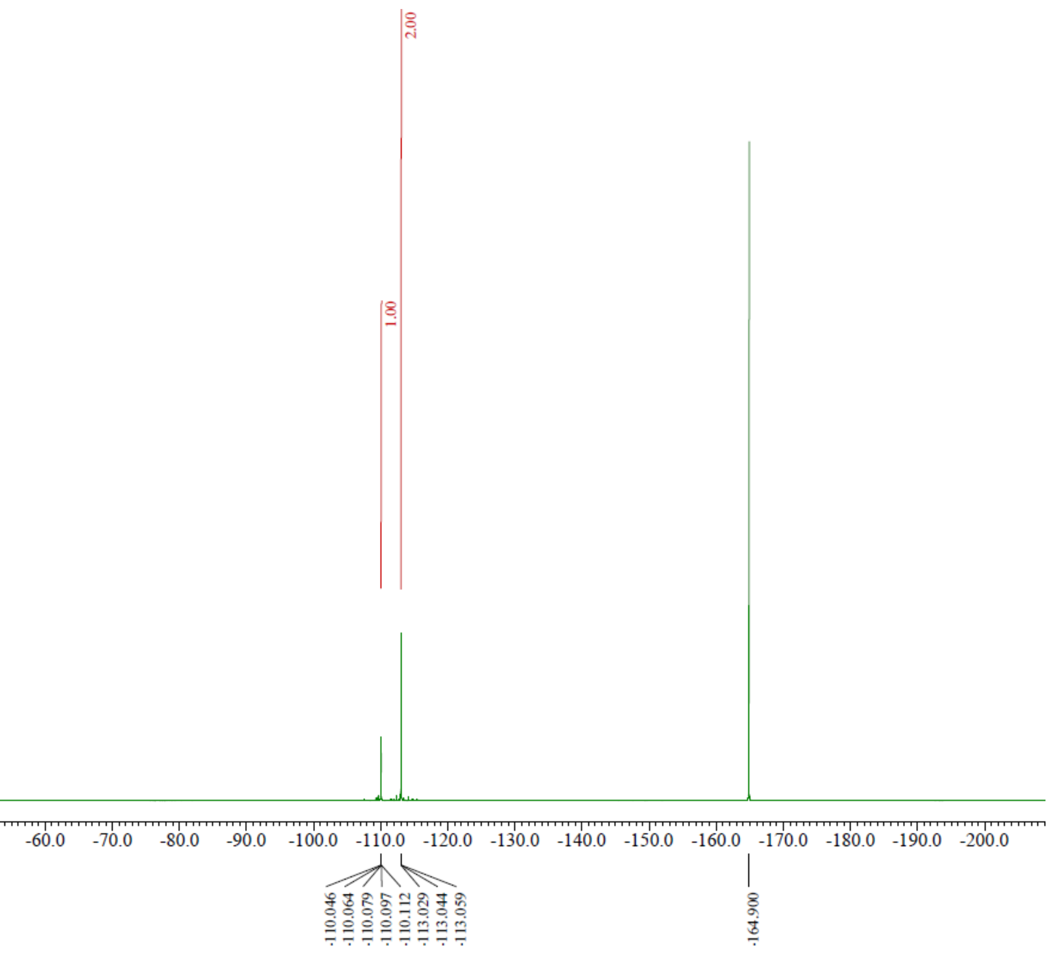

^{1}H-NMR (500 MHz, $CDCl_3$)

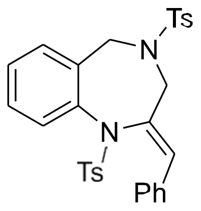

(*Z*)-3a

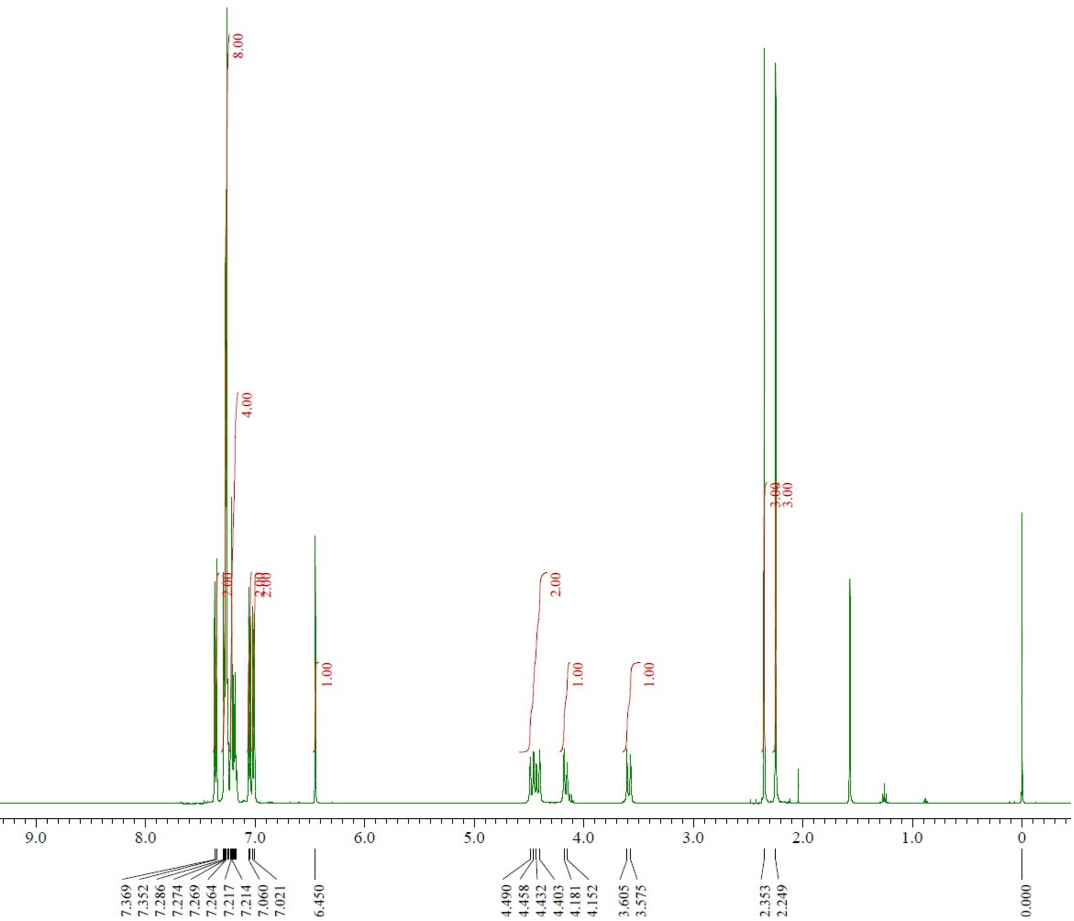

^{13}C-NMR (125 MHz, $CDCl_3$)

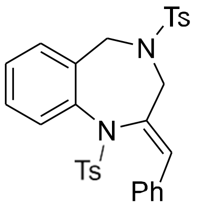

(*Z*)-3a

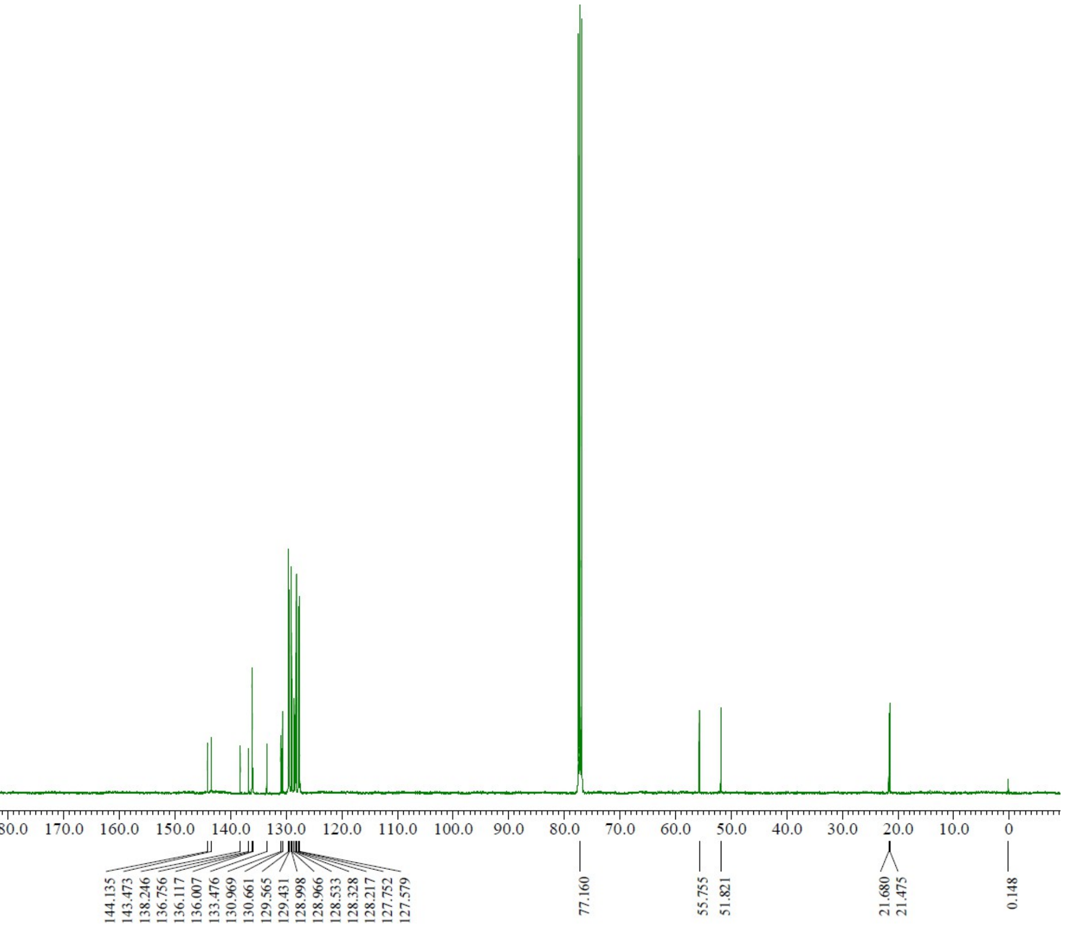

^{1}H-NMR (500 MHz, $CDCl_3$)

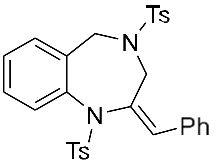

(*E*)-3a

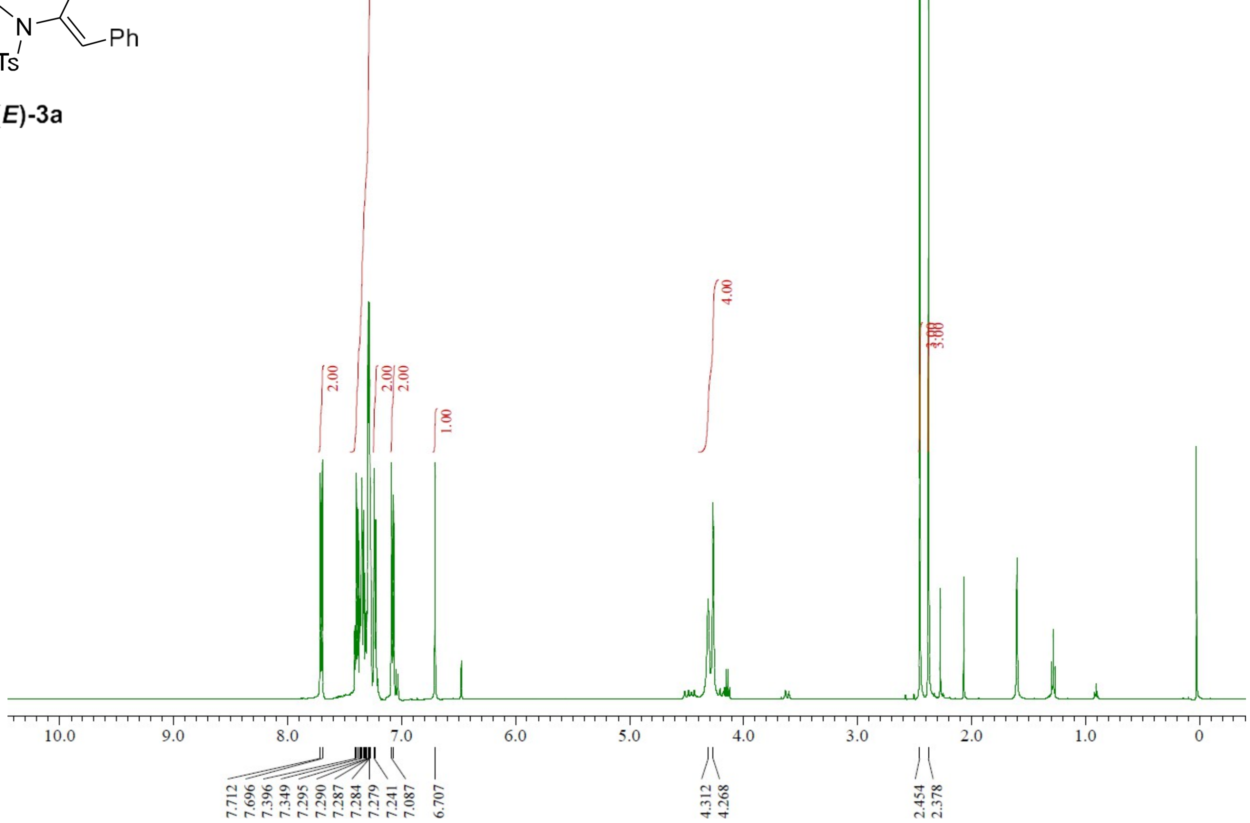

^{13}C-NMR (125 MHz, $CDCl_3$)

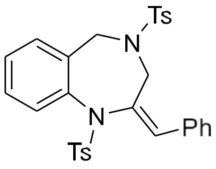

(*E*)-3a

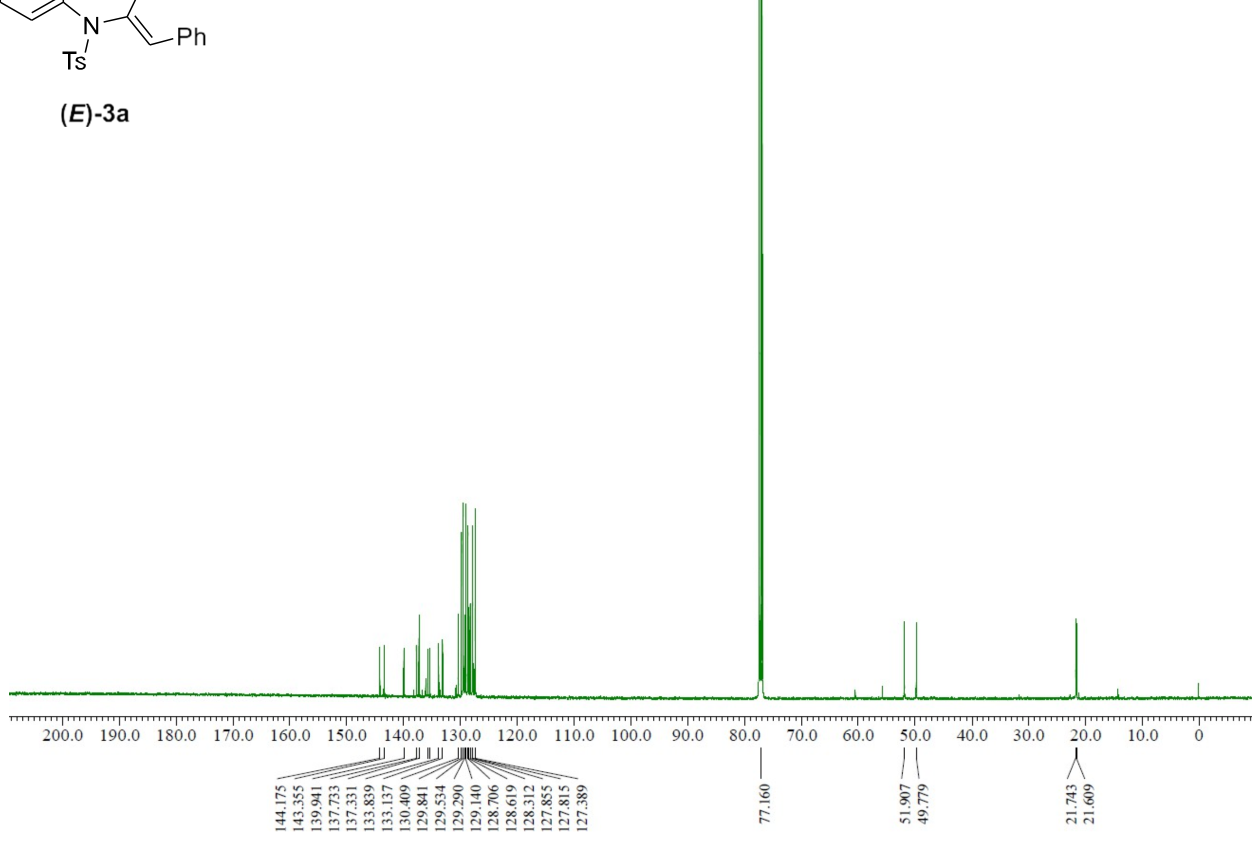

^{1}H-NMR (500 MHz, $CDCl_3$)

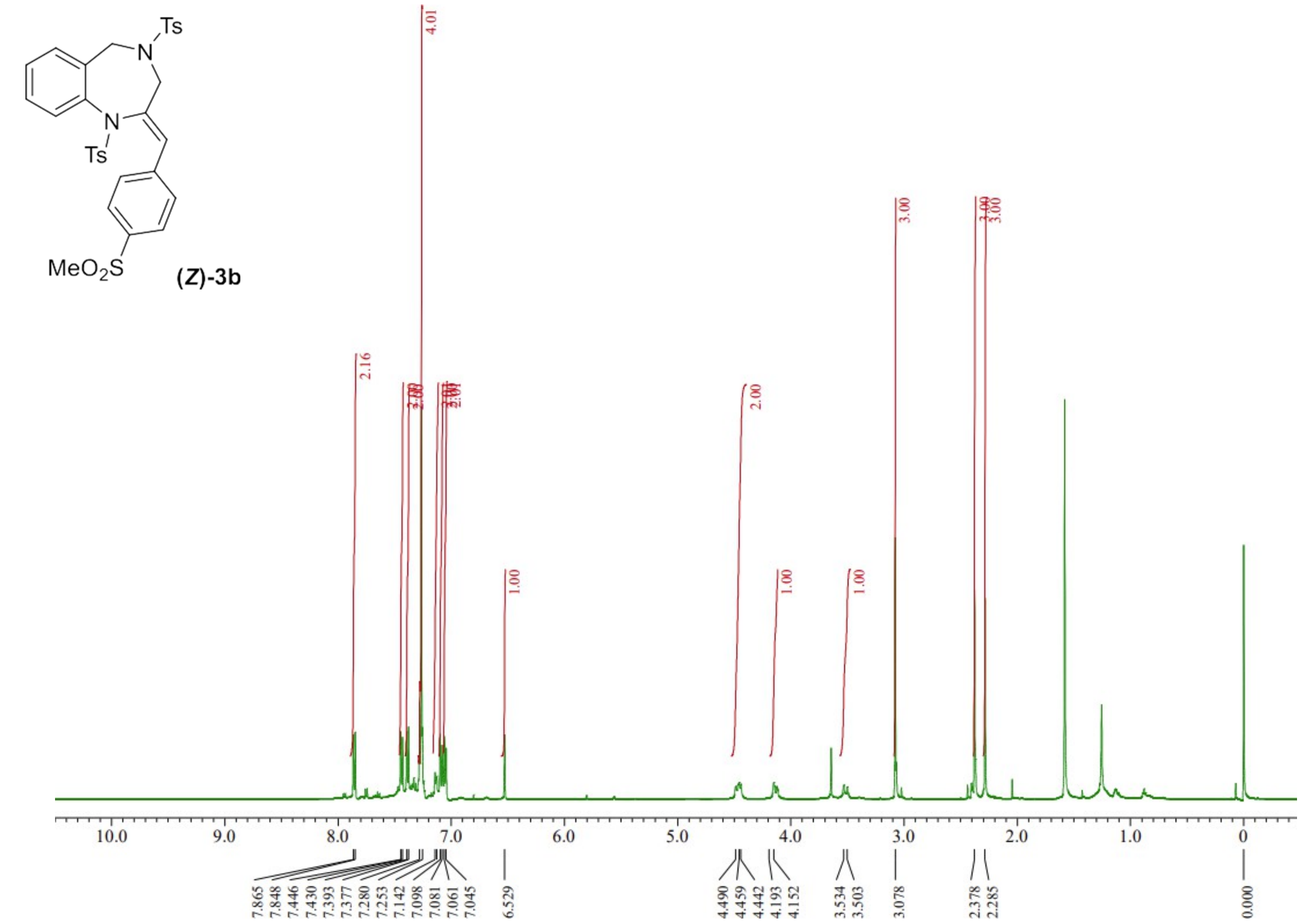

^{13}C-NMR (125 MHz, $CDCl_3$)

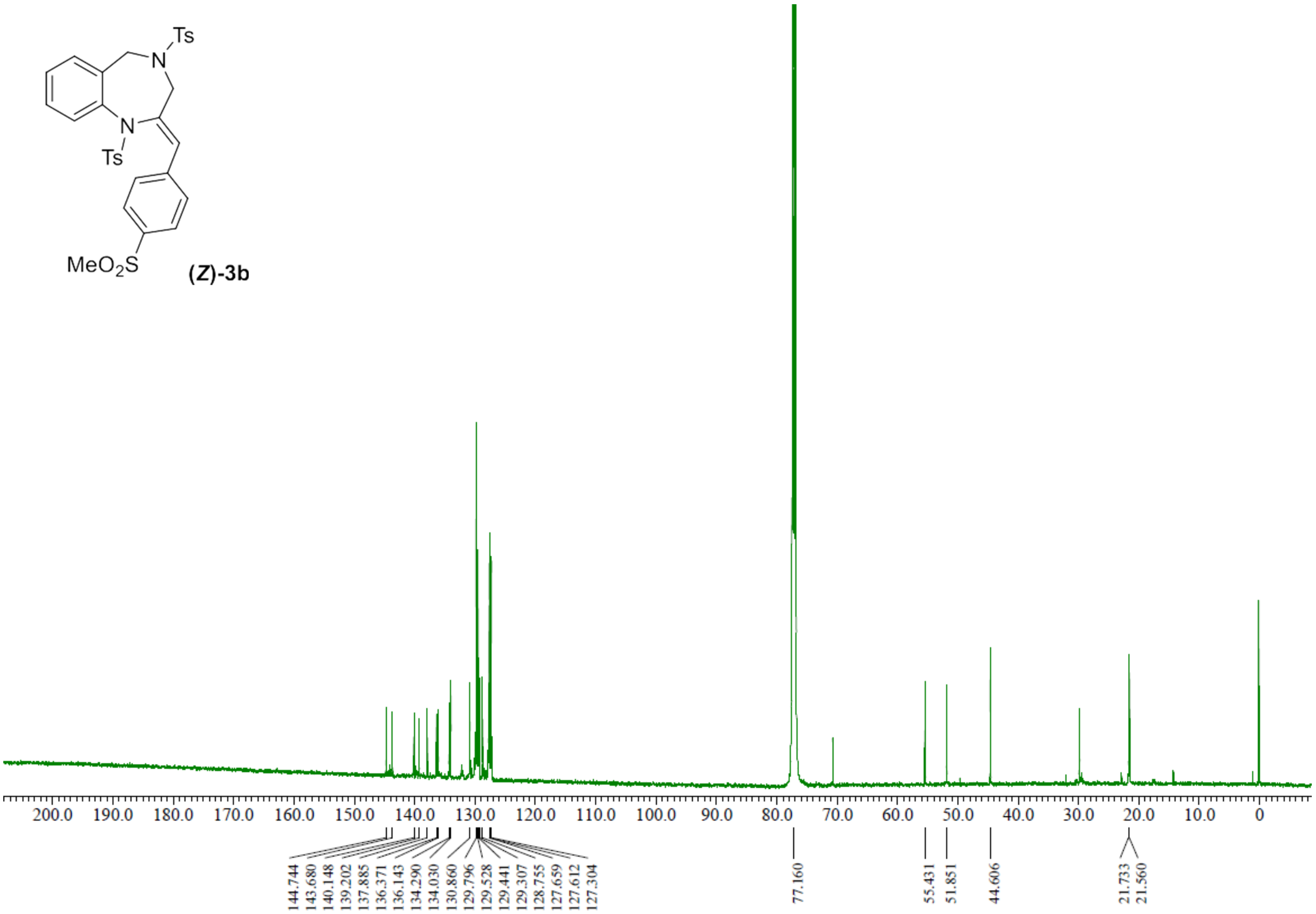

^{1}H-NMR (500 MHz, $CDCl_3$)

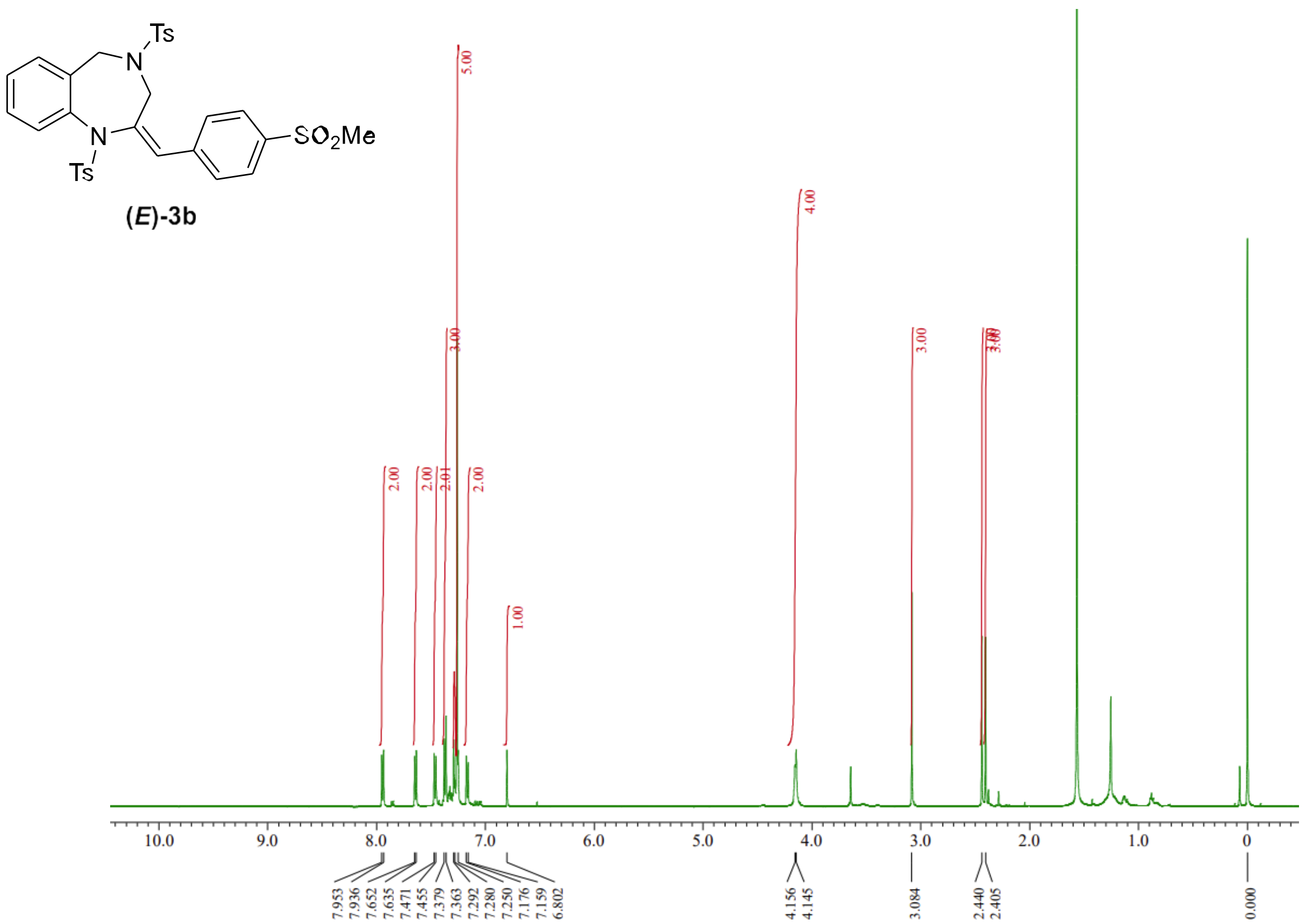

^{13}C-NMR (125 MHz, $CDCl_3$)

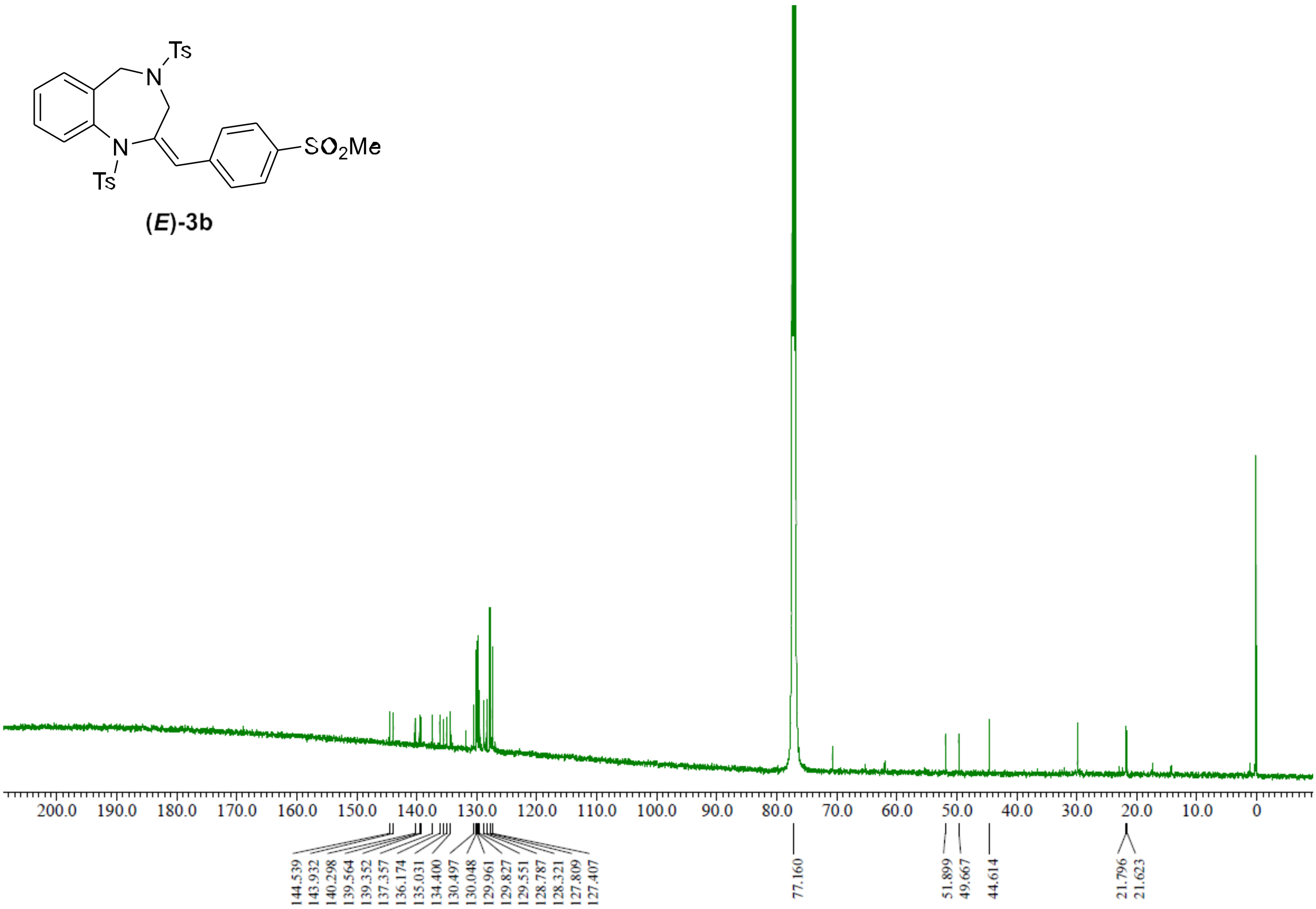

^{1}H-NMR (500 MHz, $CDCl_3$)

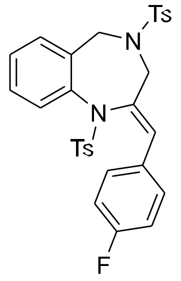

(Z)-3c

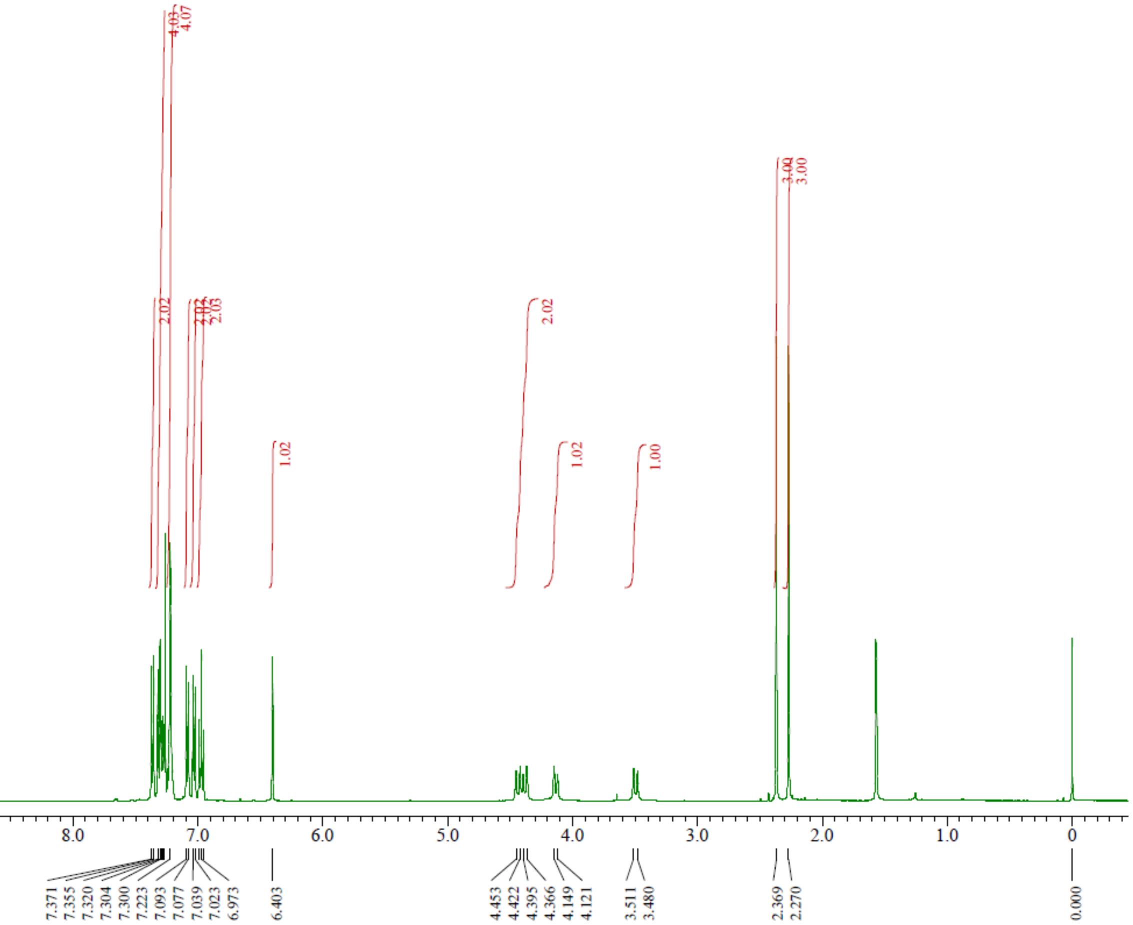

^{13}C-NMR (125 MHz, $CDCl_3$)

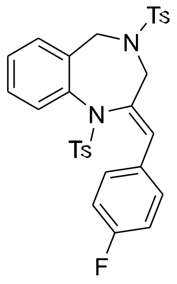

(Z)-3c

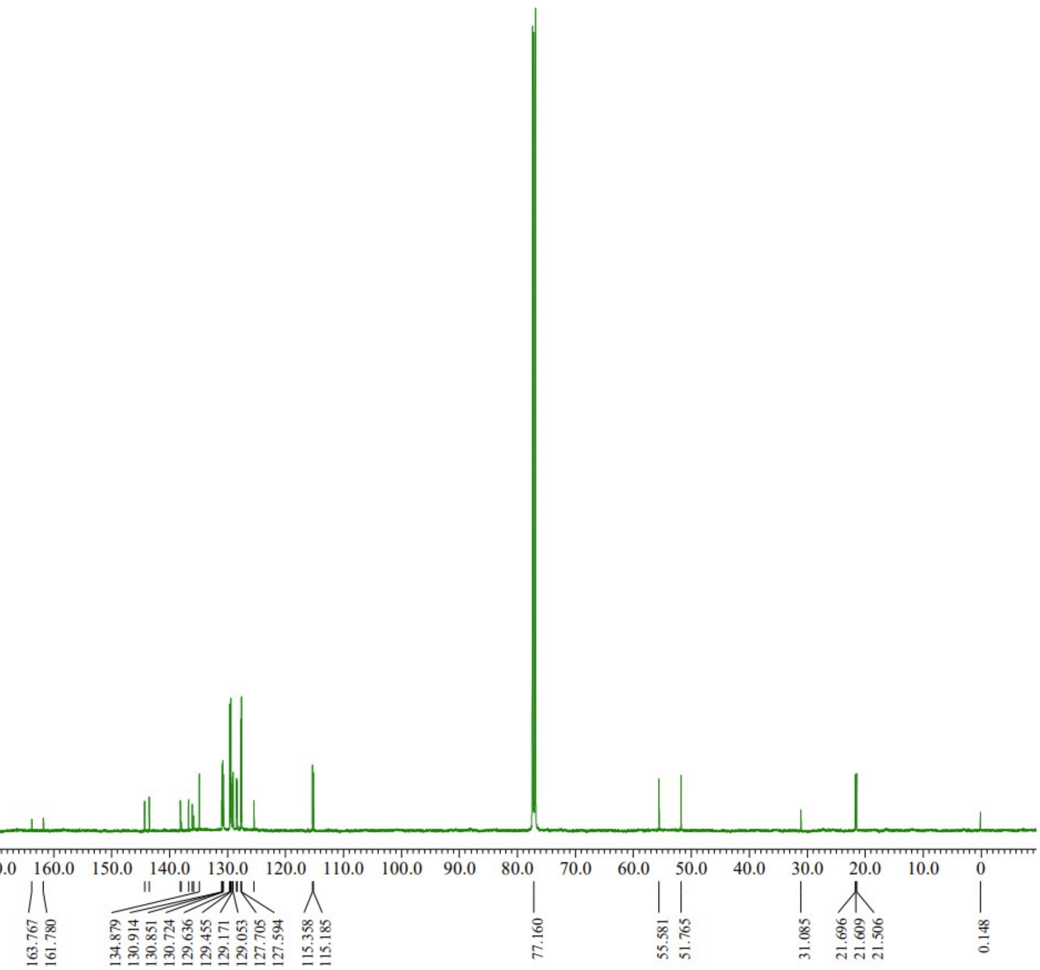

^{19}F-NMR (376 MHz, $CDCl_3$)

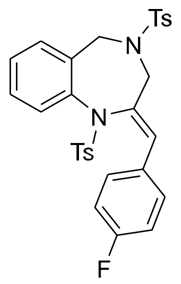

(*Z*)-3c

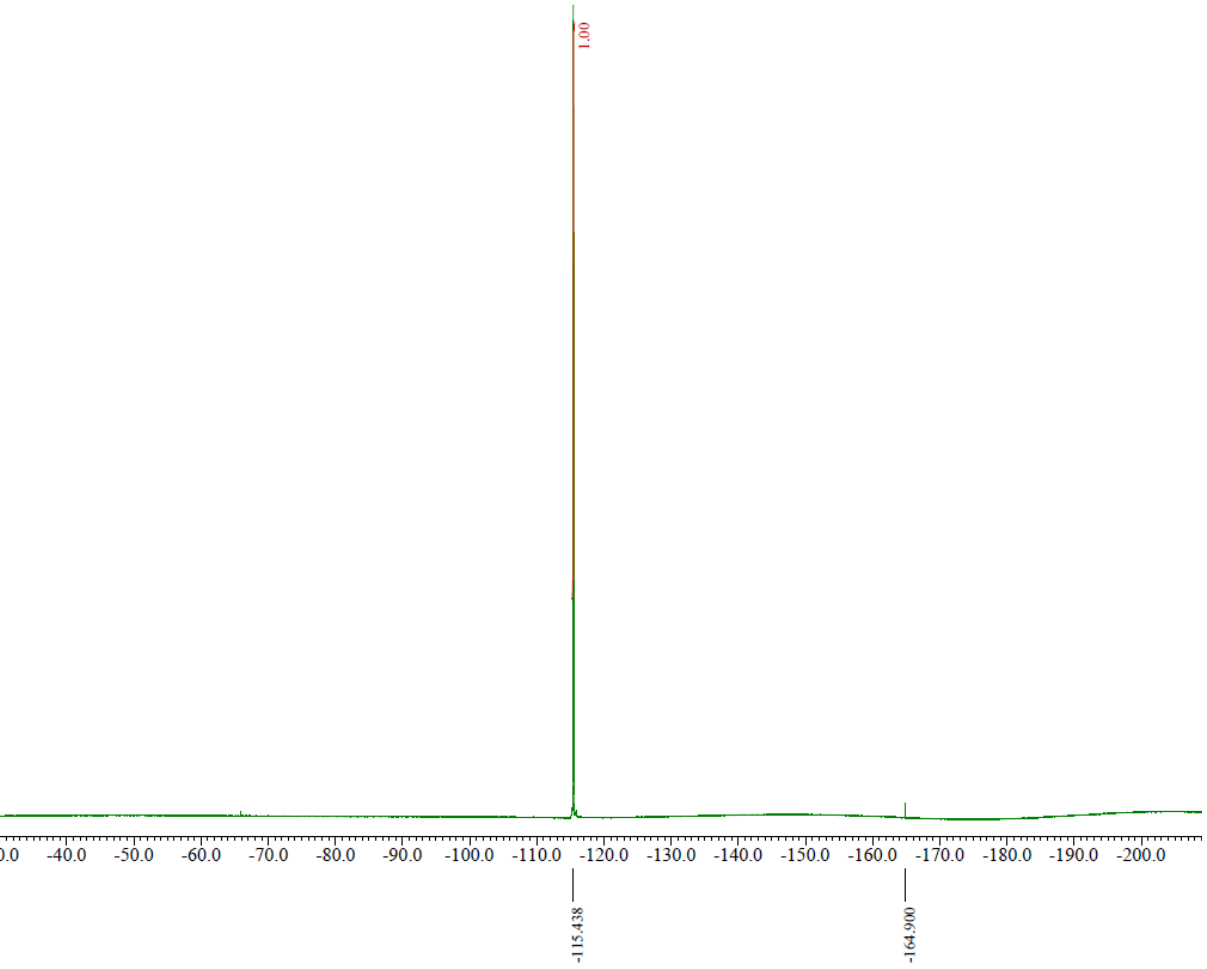

^{1}H-NMR (500 MHz, $CDCl_3$)

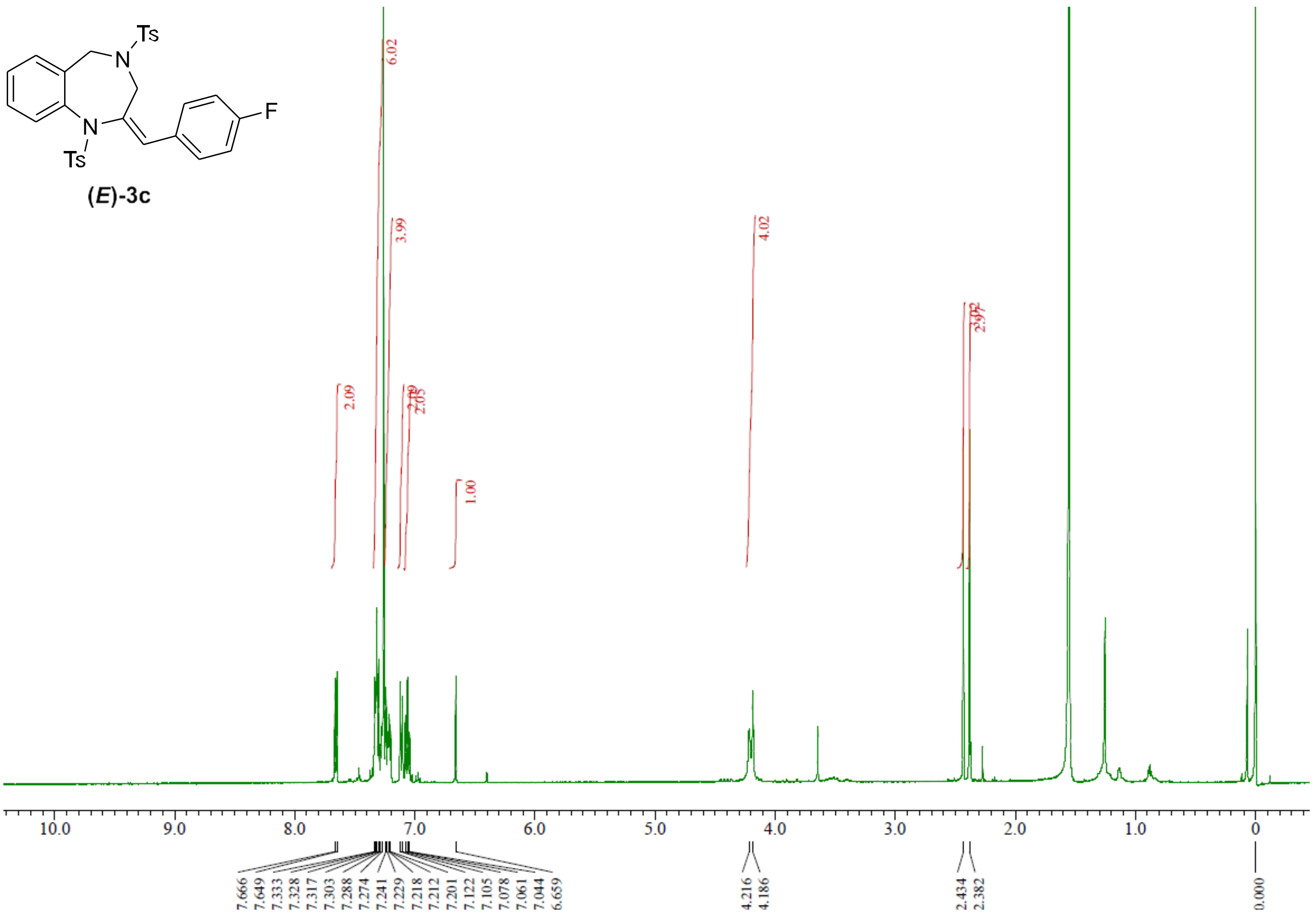

^{13}C-NMR (125 MHz, $CDCl_3$)

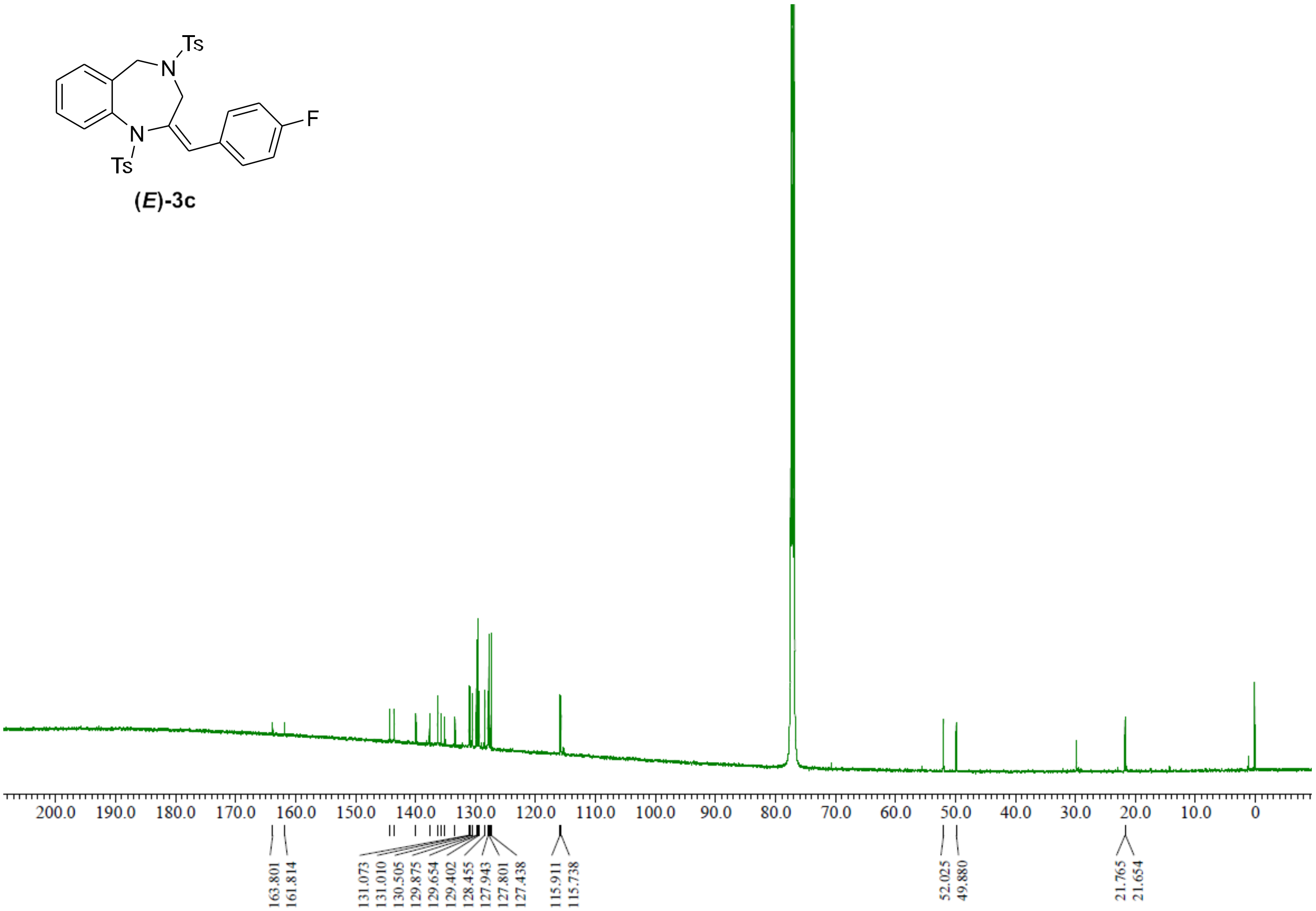

^{19}F-NMR (376 MHz, $CDCl_3$)

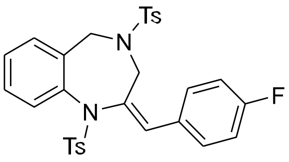

(*E*)-3c

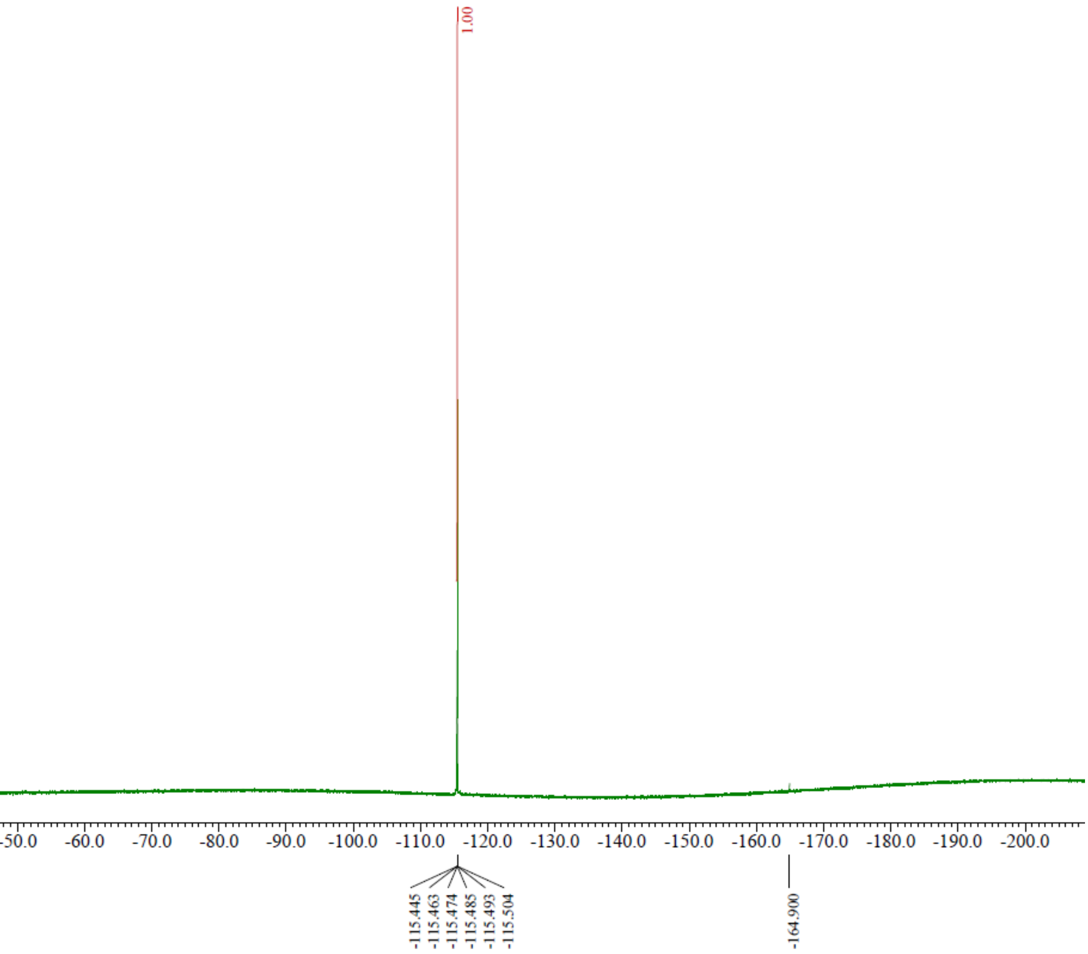

^{1}H-NMR (500 MHz, $CDCl_3$)

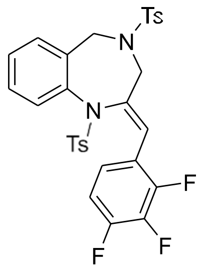

(*Z*)-3d

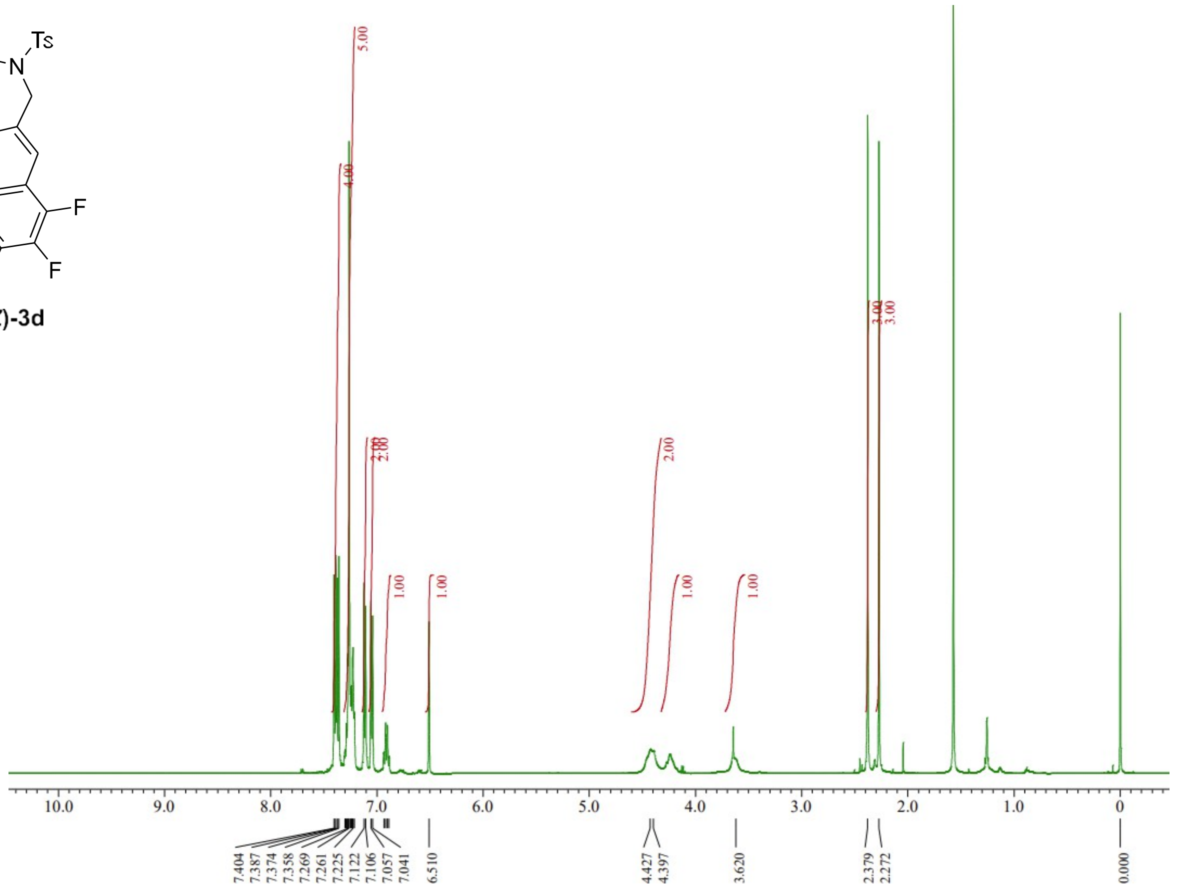

^{13}C-NMR (125 MHz, $CDCl_3$)

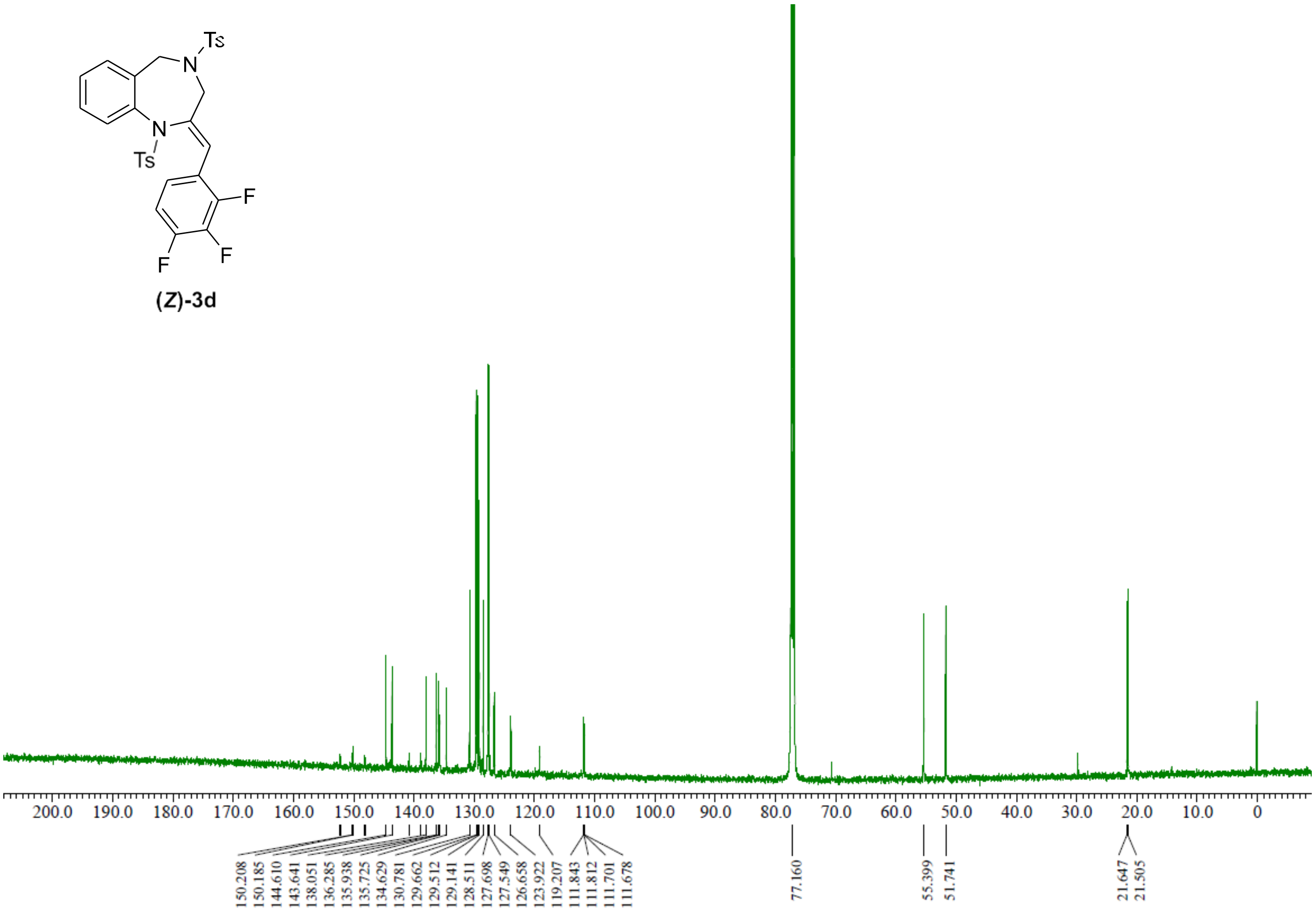

^{19}F-NMR (376 MHz, $CDCl_3$)

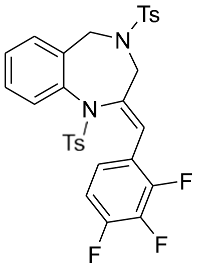

(*Z*)-3d

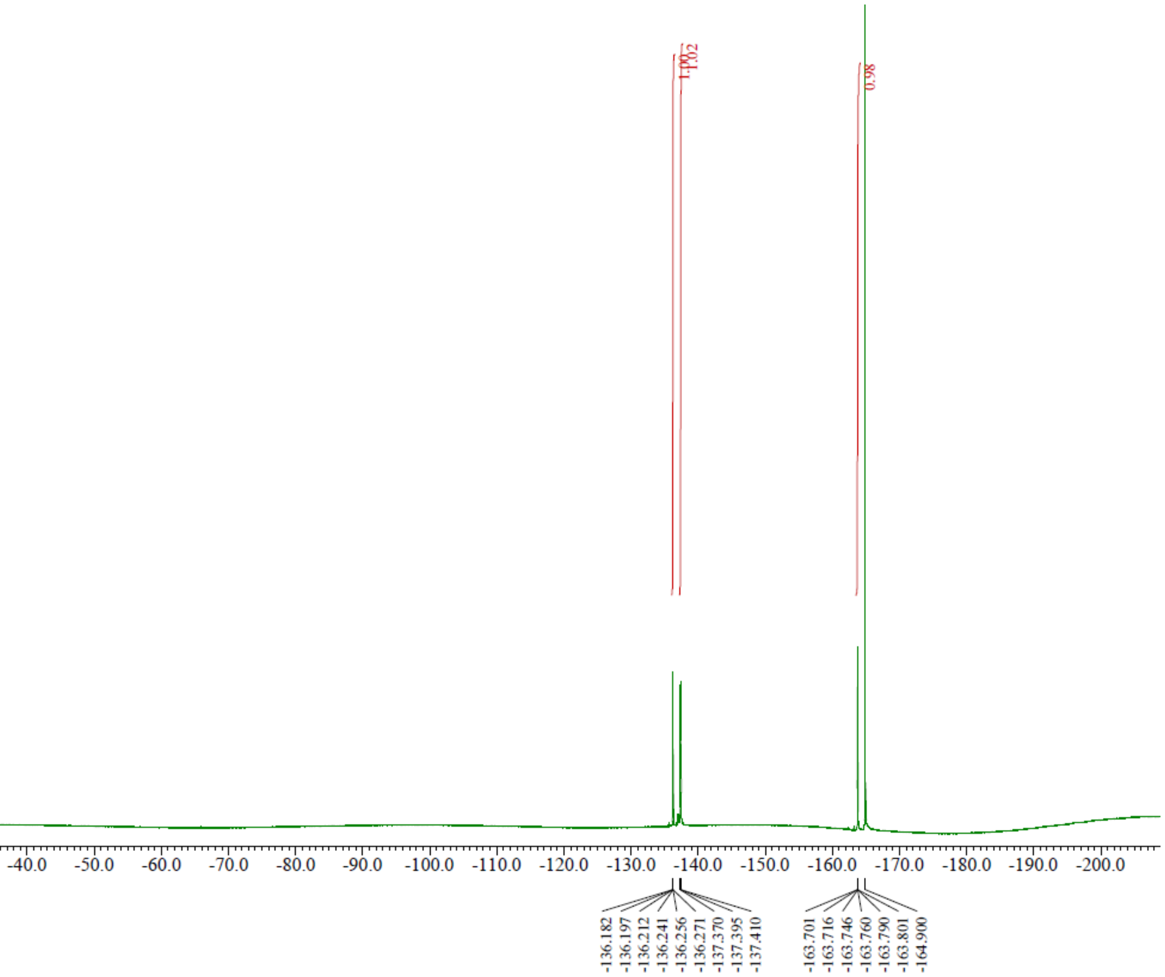

^{1}H-NMR (500 MHz, $CDCl_3$)

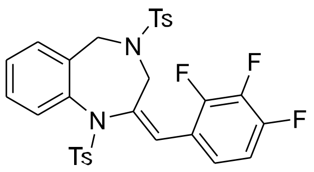

(*E*)-3d

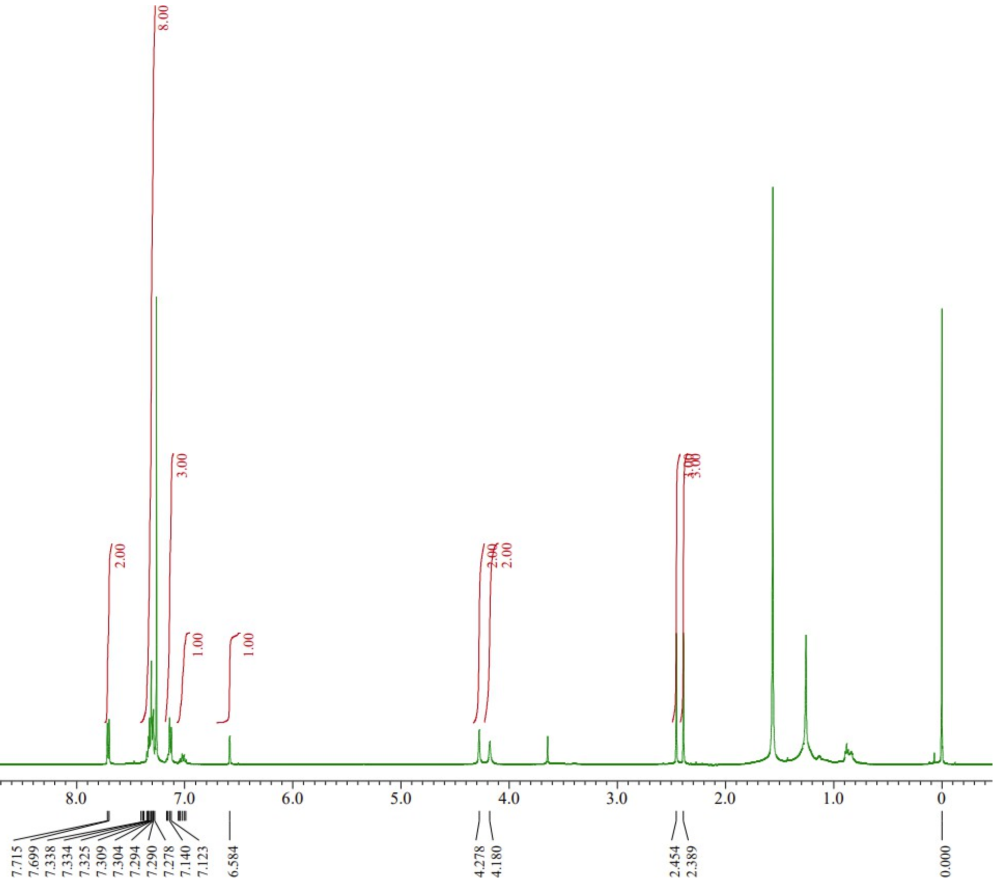

^{13}C-NMR (125 MHz, $CDCl_3$)

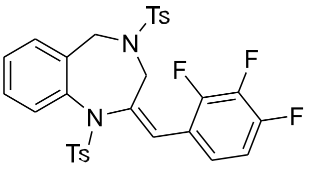

(*E*)-3d

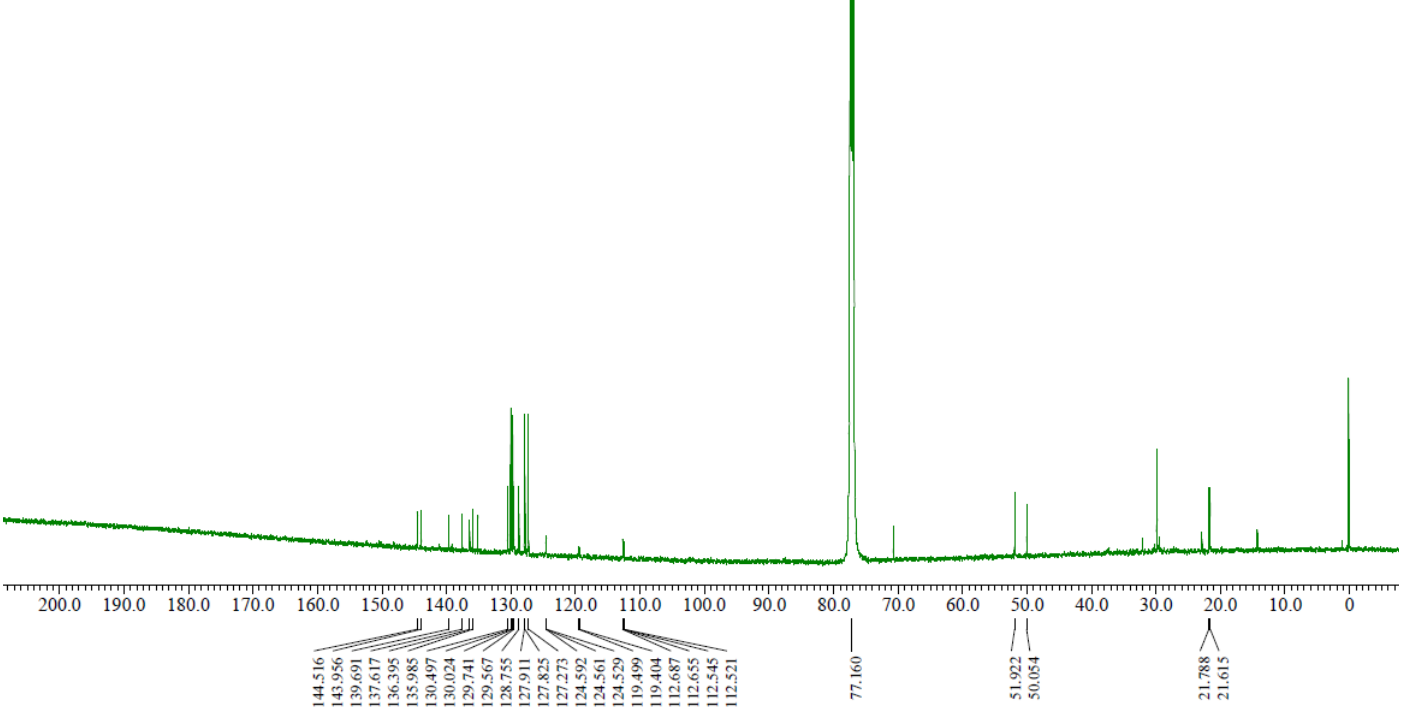

^{19}F-NMR (376 MHz, $CDCl_3$)

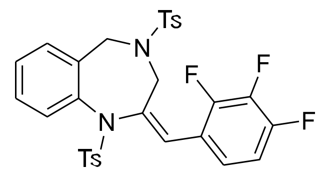

(*E*)-3d

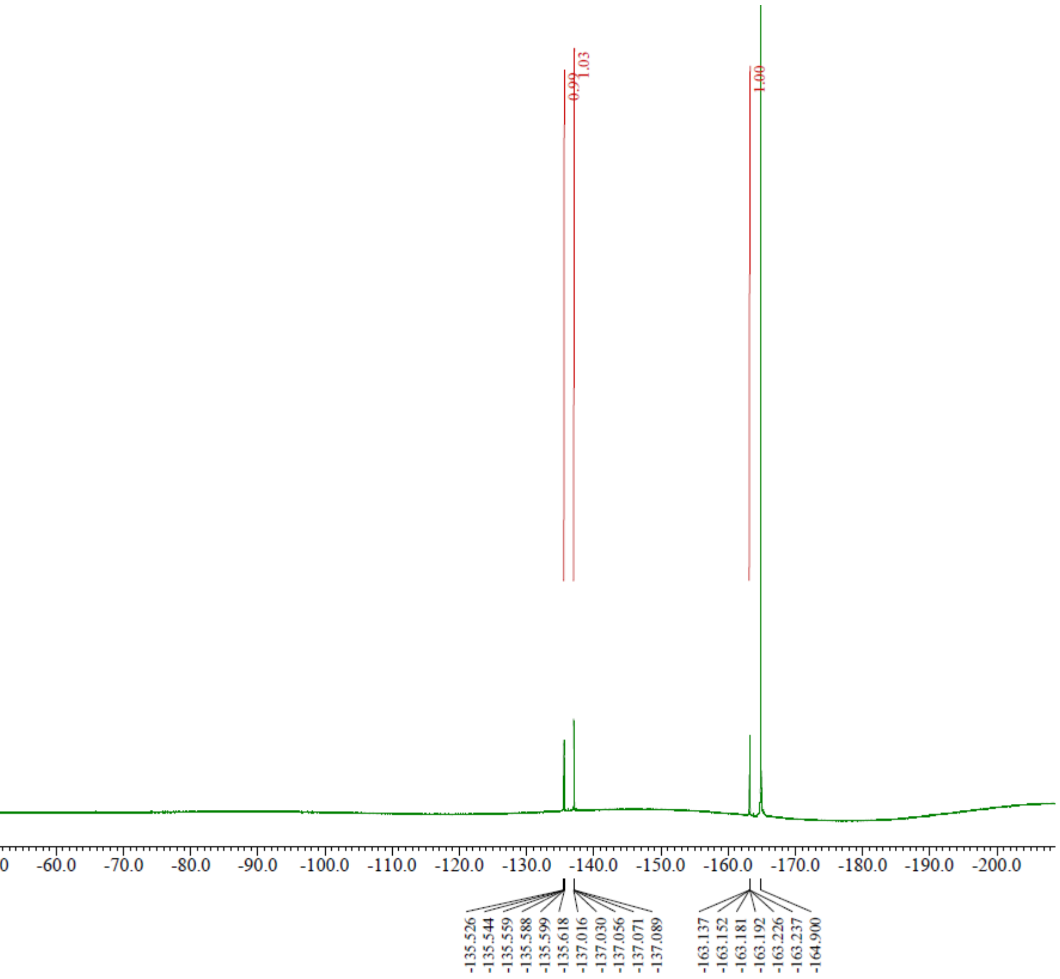

^{1}H-NMR (500 MHz, $CDCl_3$)

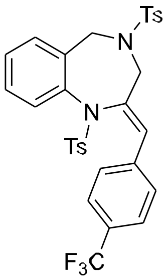

(Z)-3e

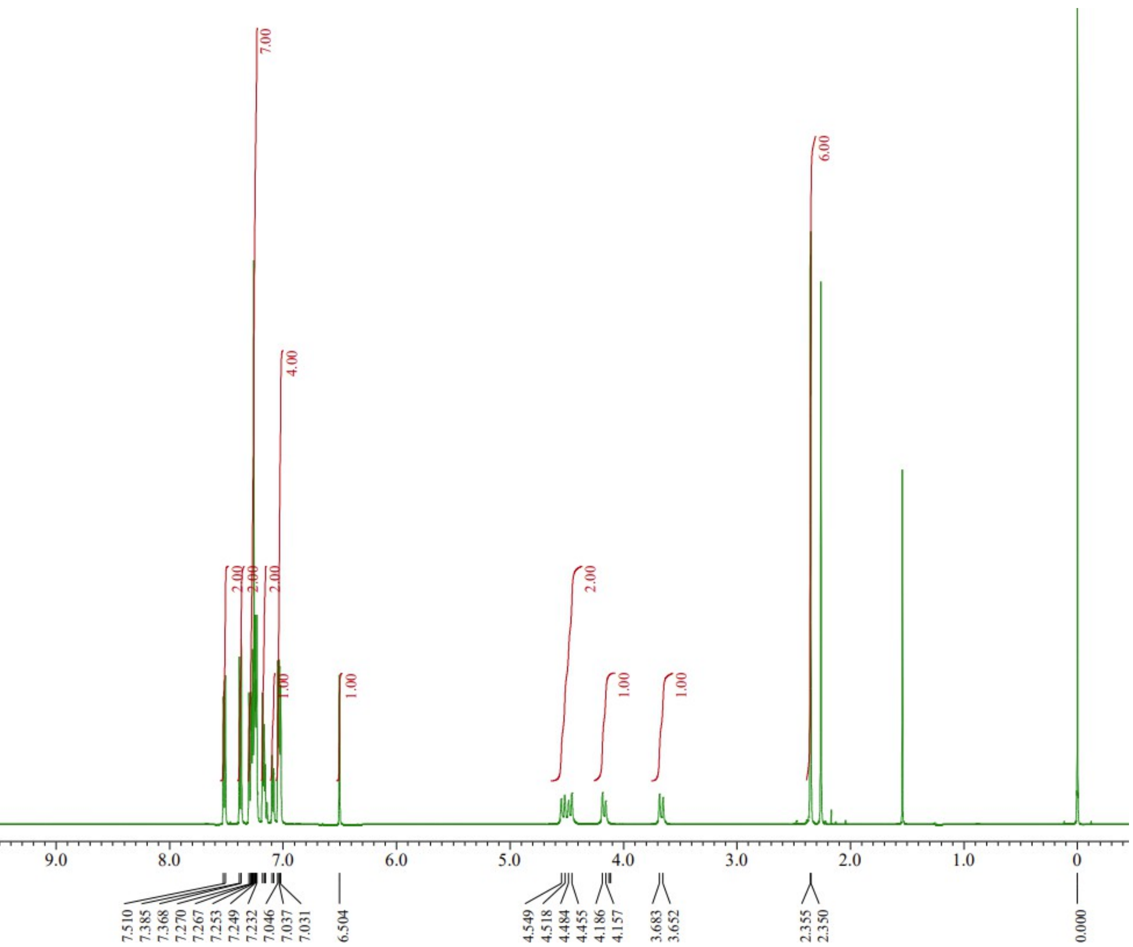

^{13}C-NMR (125 MHz, $CDCl_3$)

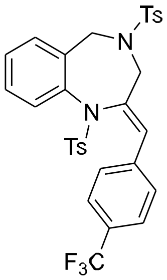

(Z)-3e

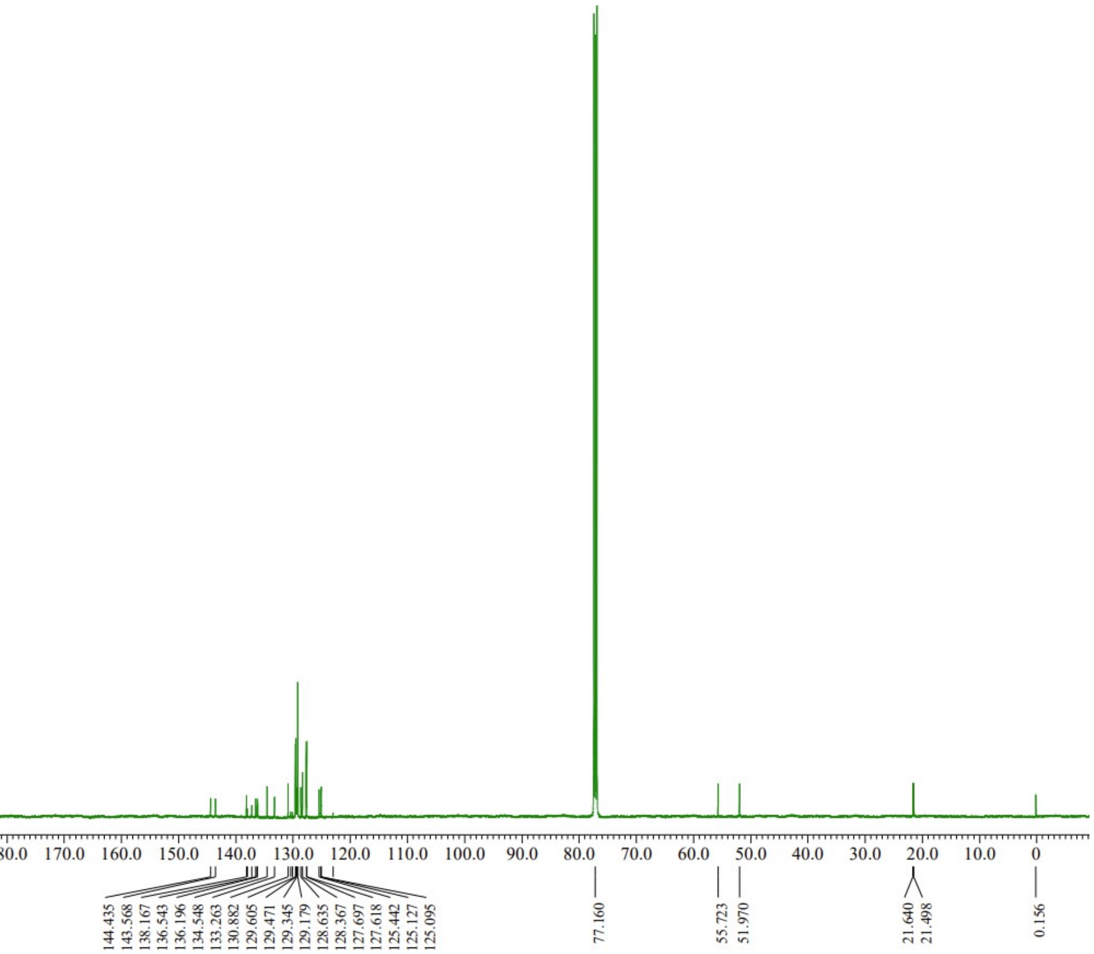

^{19}F-NMR (376 MHz, $CDCl_3$)

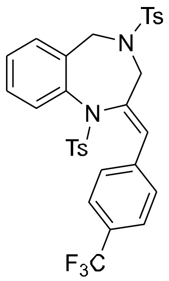

(*Z*)-3e

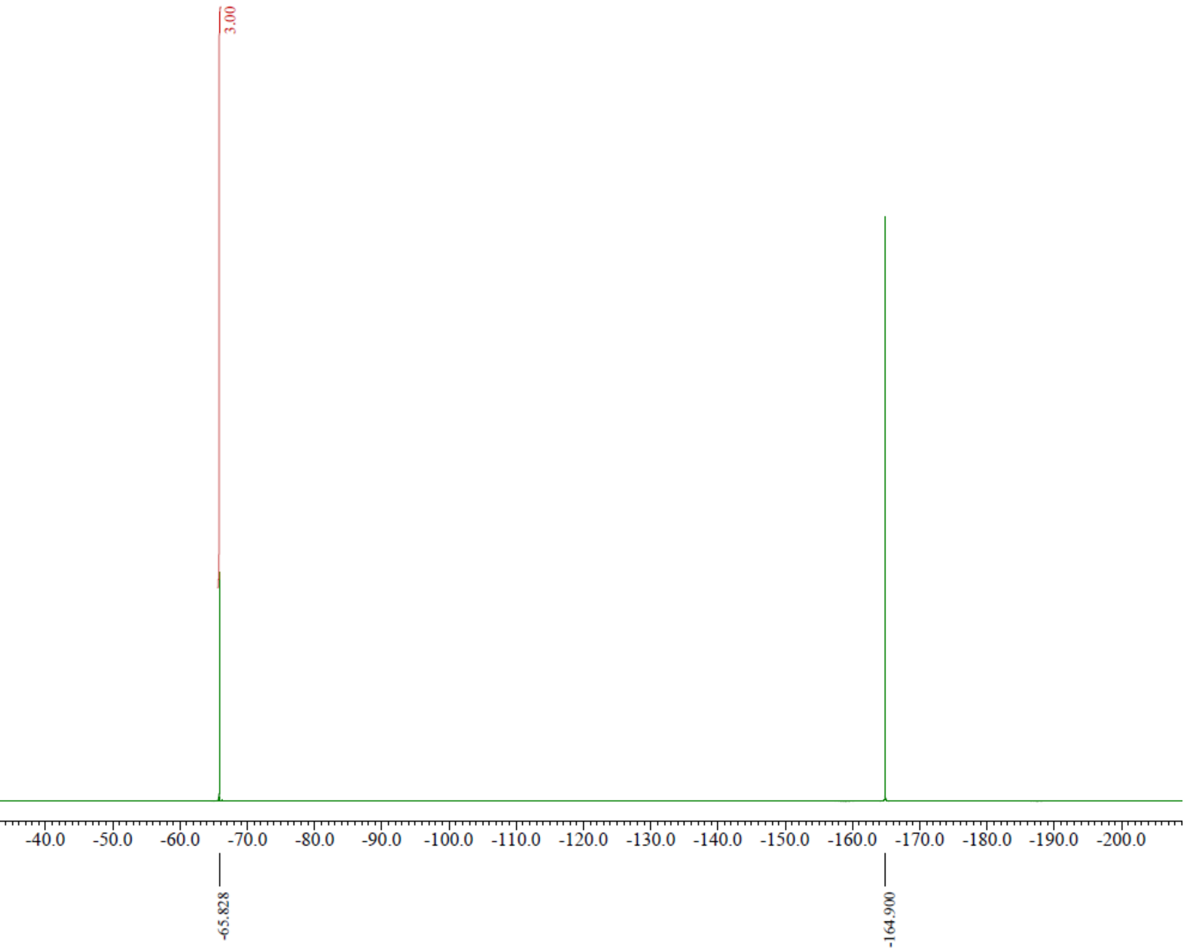

^{1}H-NMR (500 MHz, $CDCl_3$)

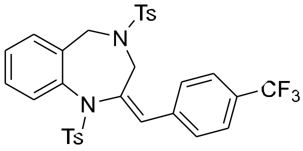

(*E*)-3e

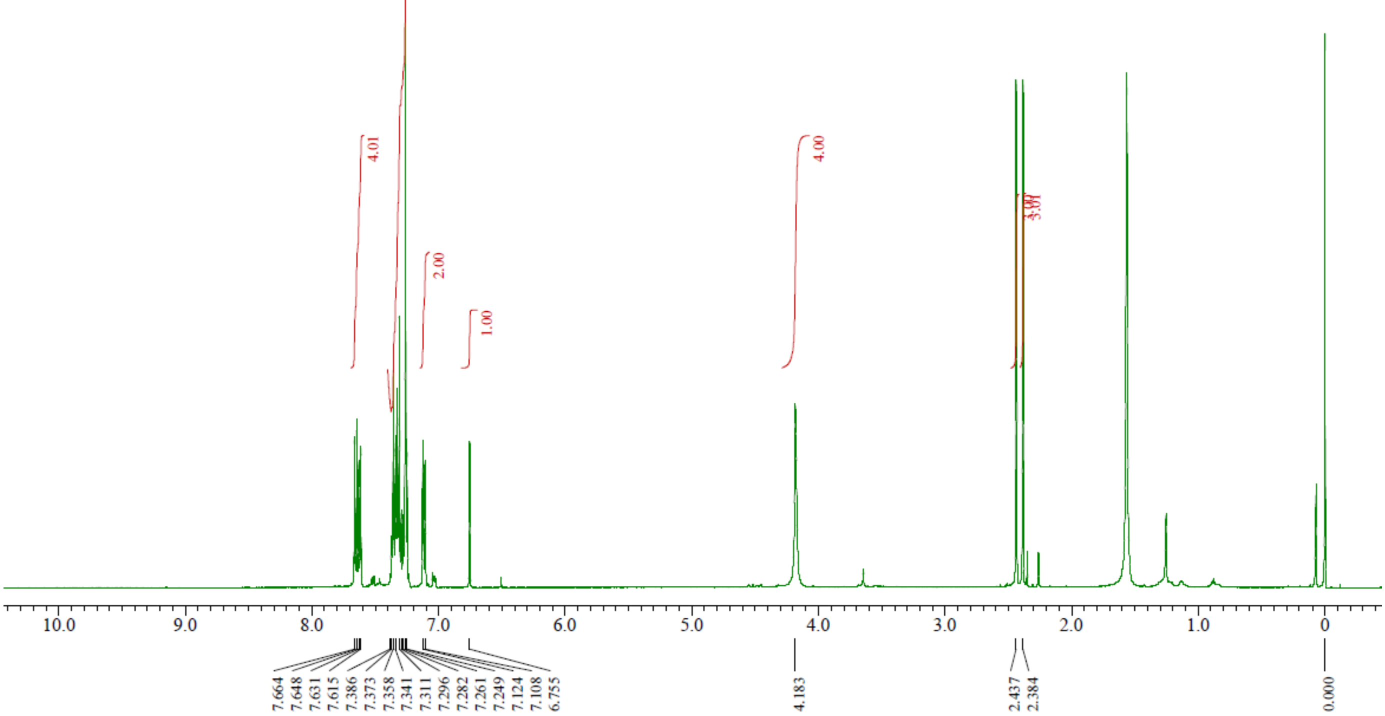

^{13}C-NMR (125 MHz, $CDCl_3$)

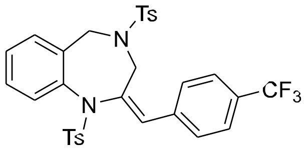

(*E*)-3e

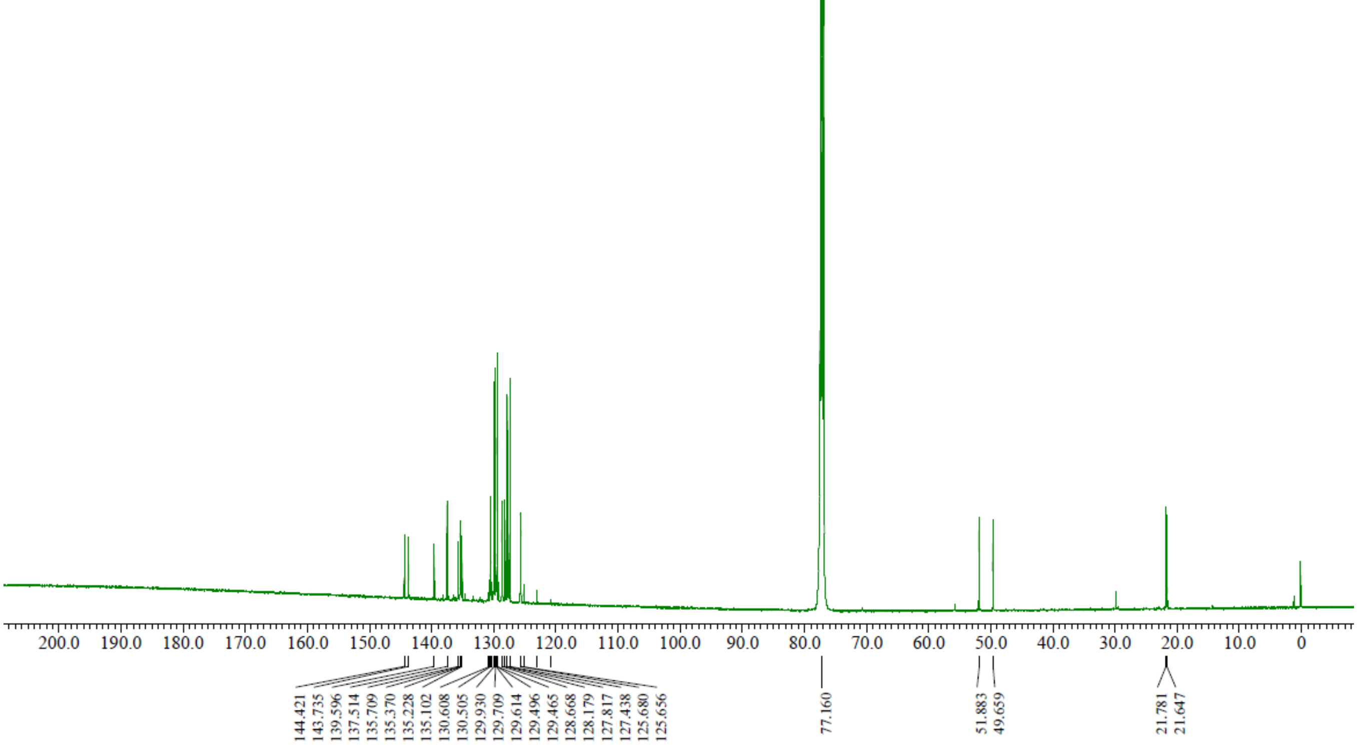

^{19}F-NMR (376 MHz, $CDCl_3$)

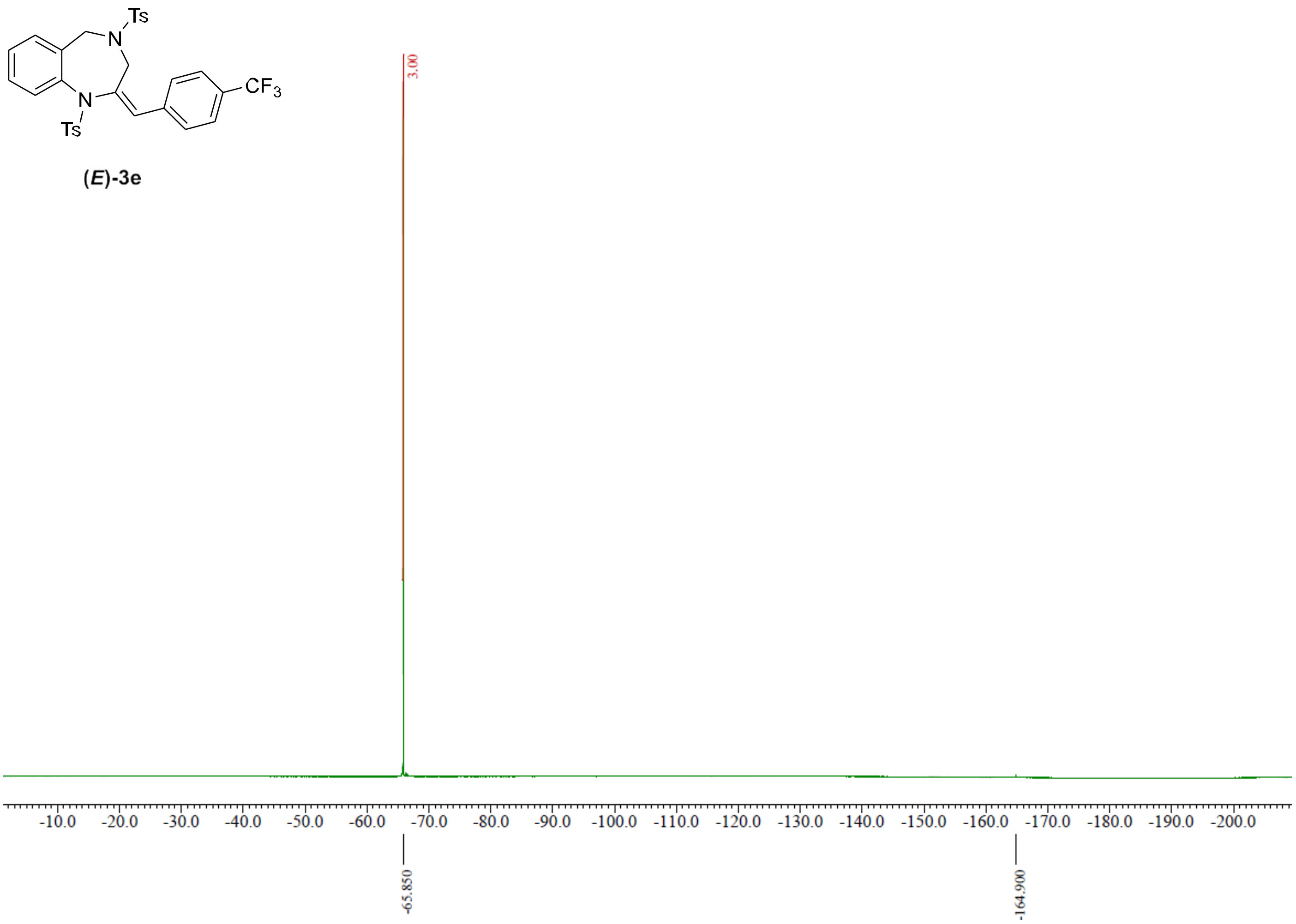

^{1}H-NMR (500 MHz, $CDCl_3$)

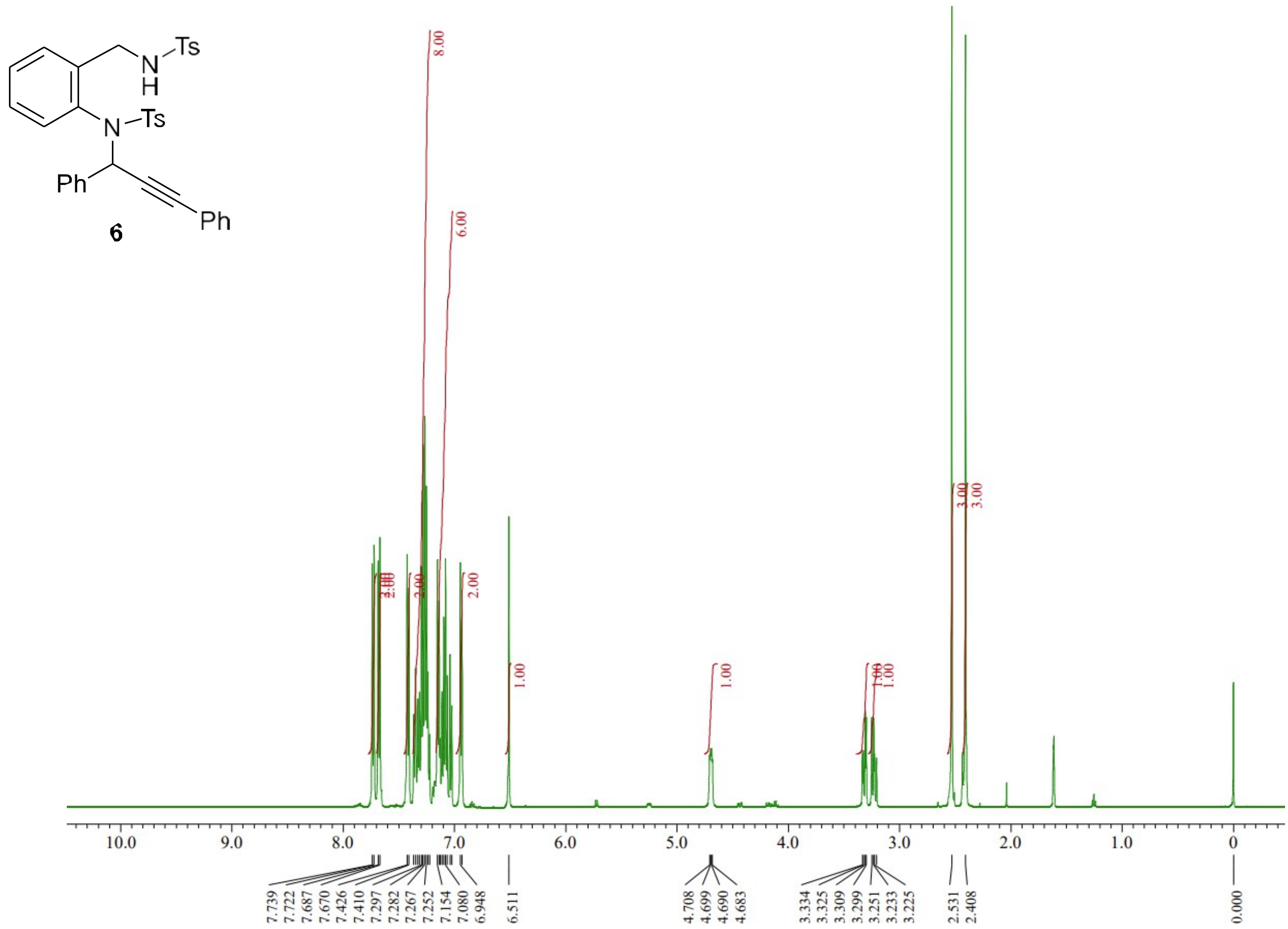

^{13}C-NMR (125 MHz, $CDCl_3$)

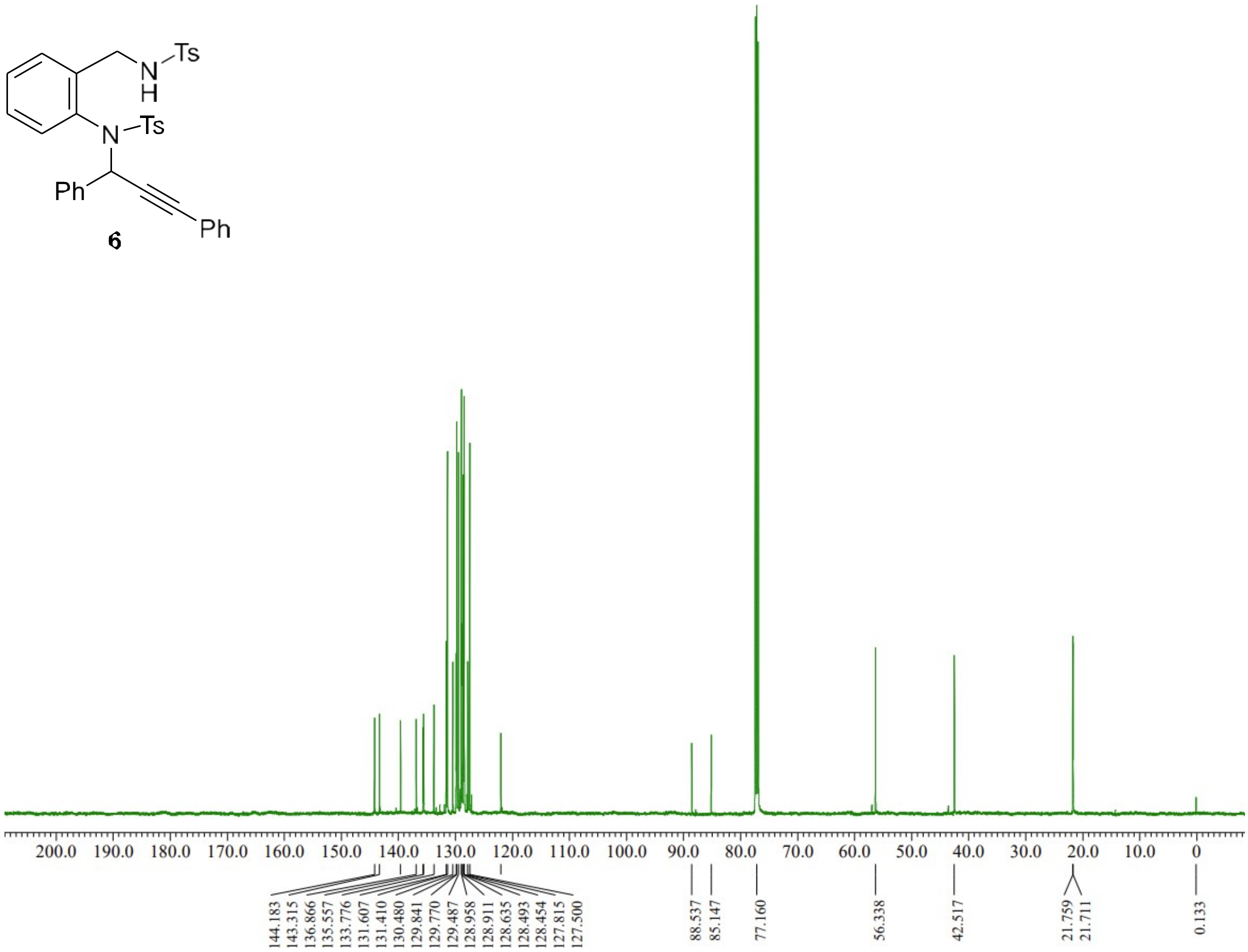

^{1}H-NMR (500 MHz, $CDCl_3$)

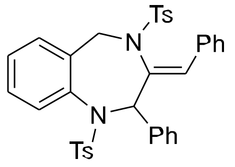

(*Z*)-7g

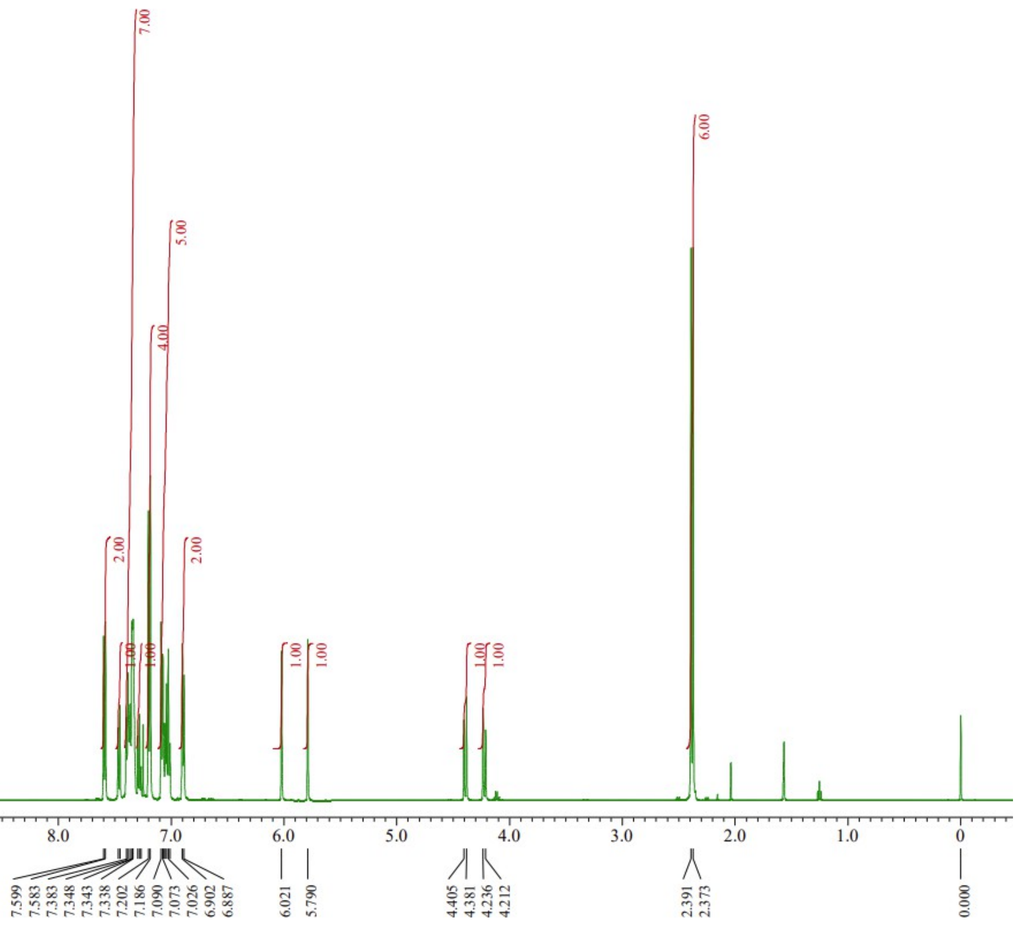

^{13}C-NMR (125 MHz, $CDCl_3$)

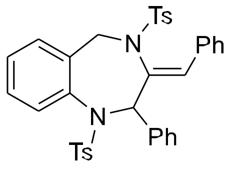

(*Z*)-7g

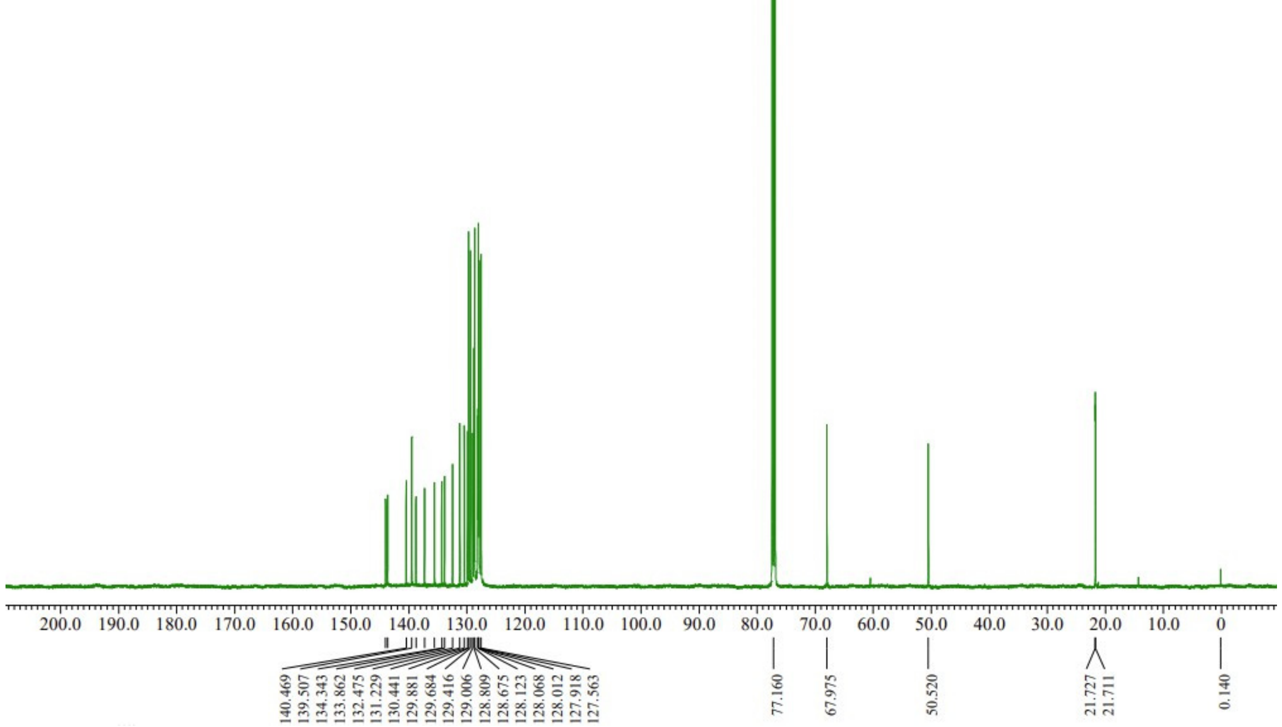

^{1}H-NMR (500 MHz, $CDCl_3$)

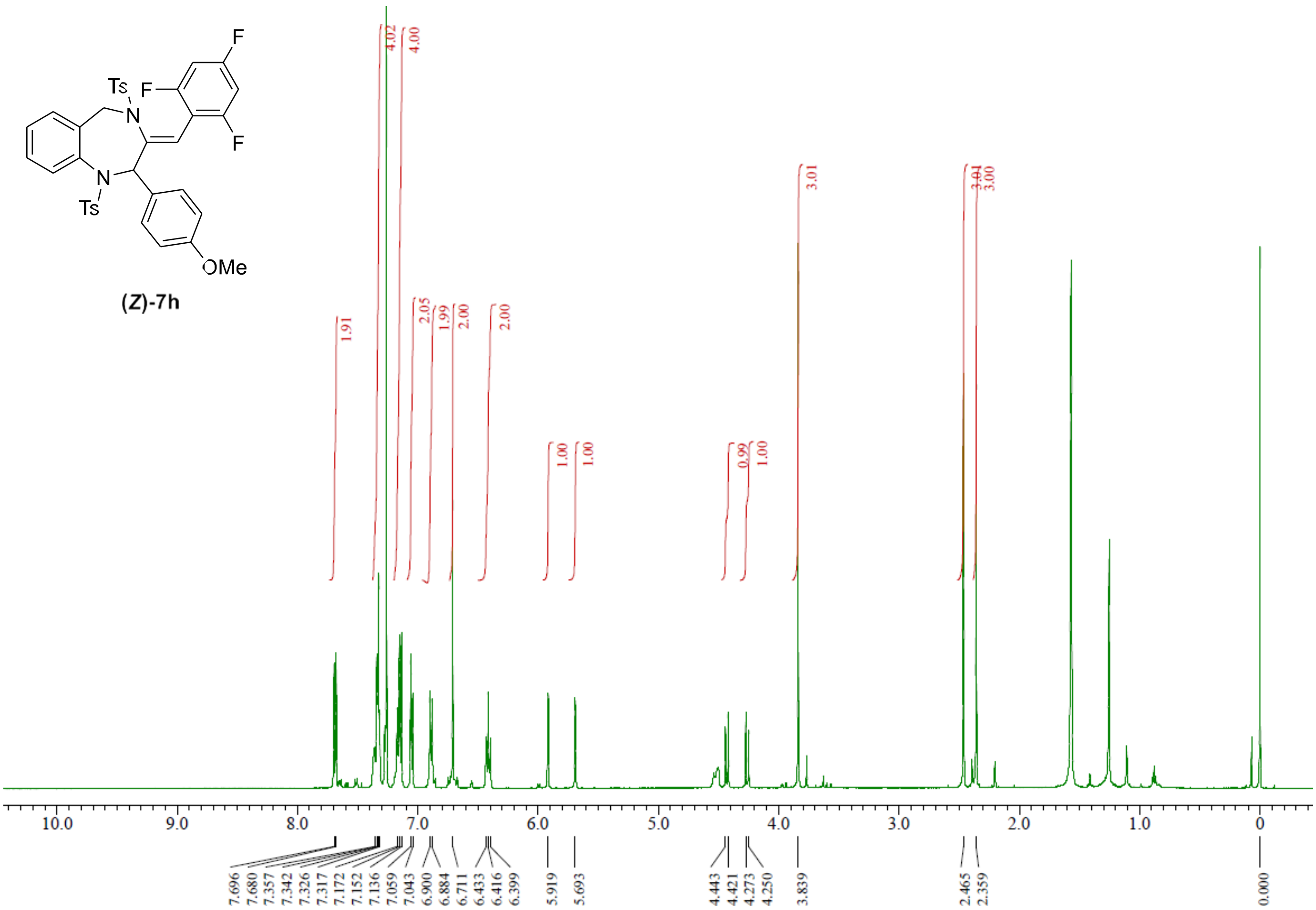

^{13}C-NMR (125 MHz, $CDCl_3$)

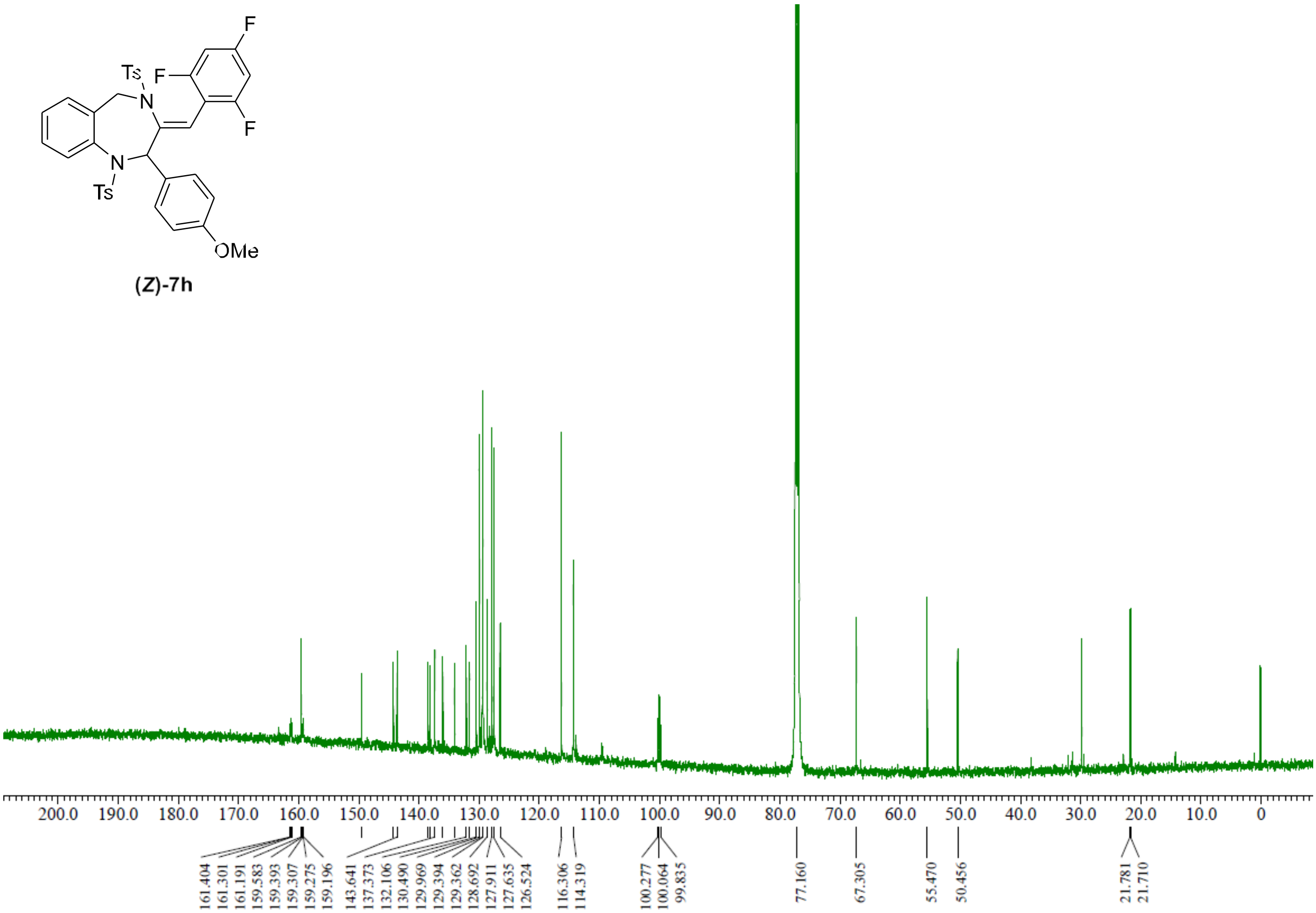

^{19}F-NMR (376 MHz, $CDCl_3$)

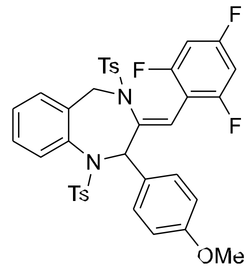

(*Z*)-7h

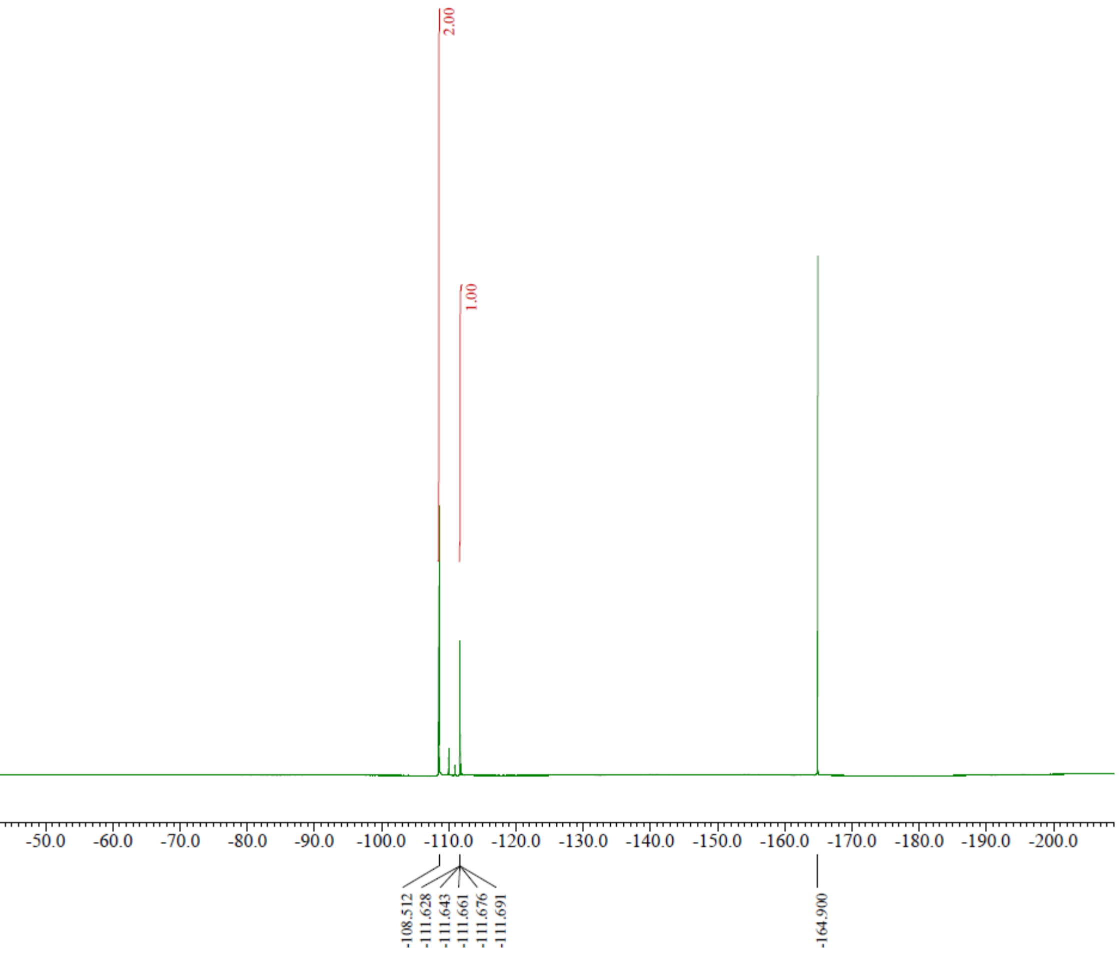

^{1}H-NMR (500 MHz, $CDCl_3$)

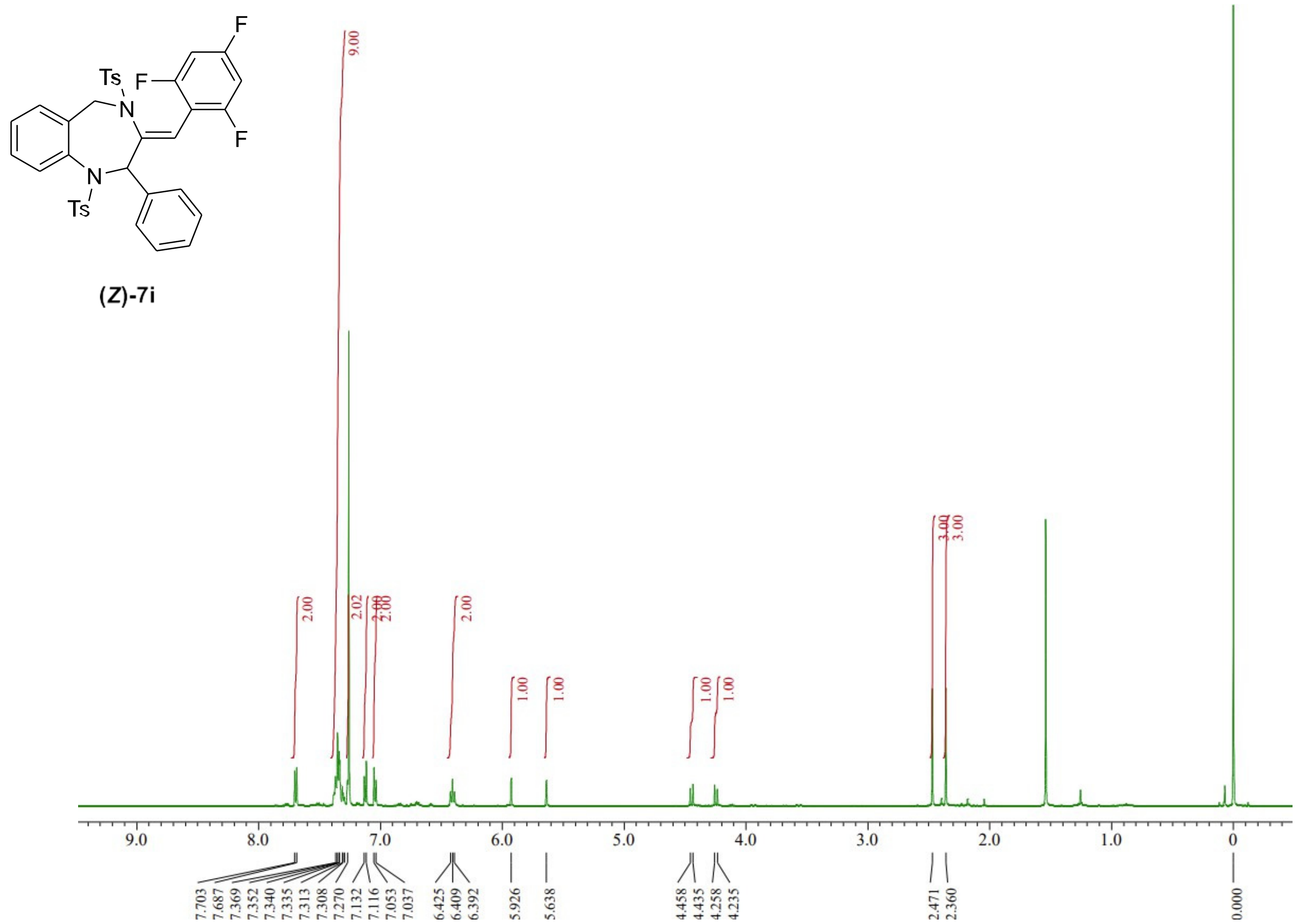

^{13}C-NMR (125 MHz, $CDCl_3$)

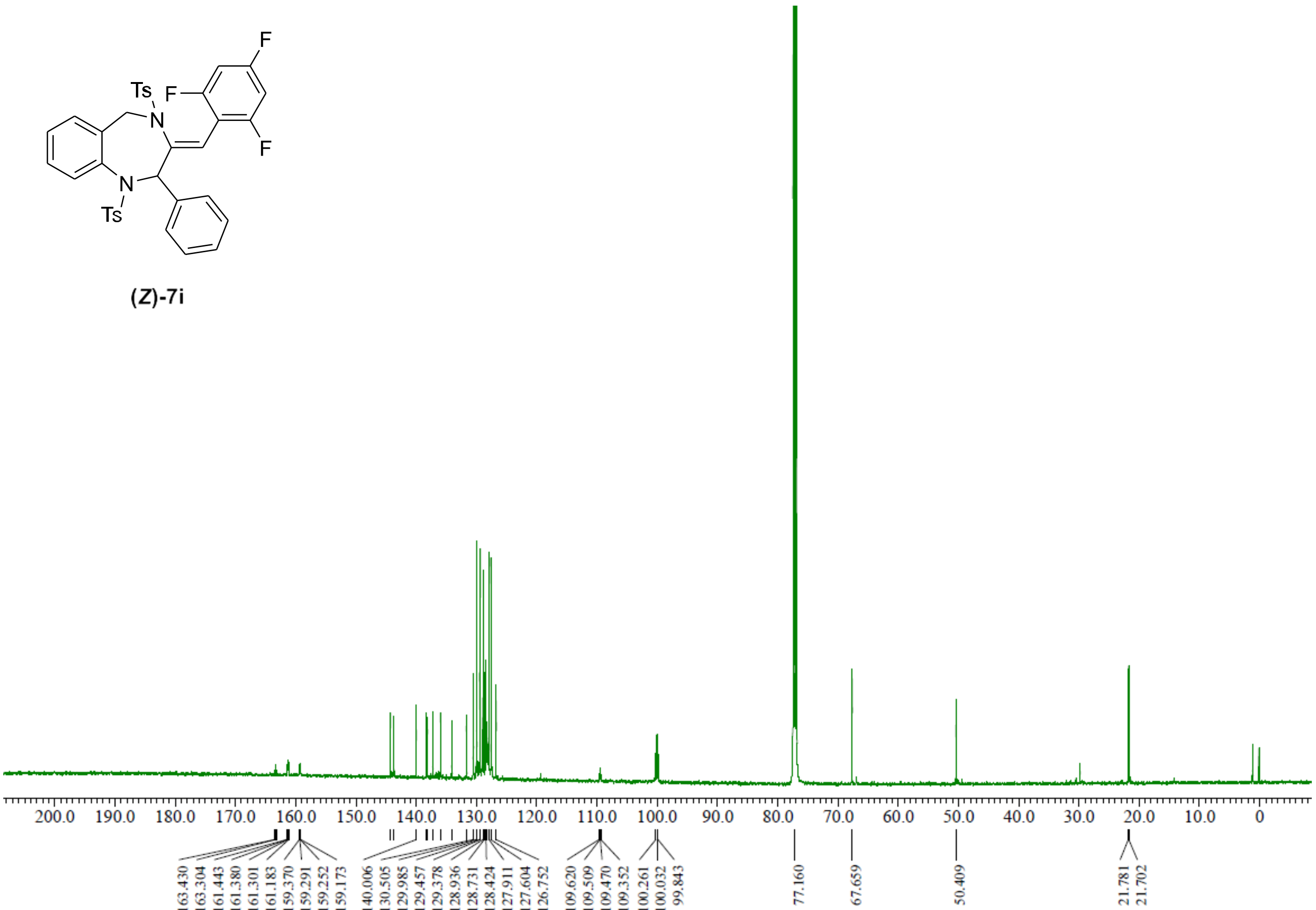

^{19}F-NMR (376 MHz, $CDCl_3$)

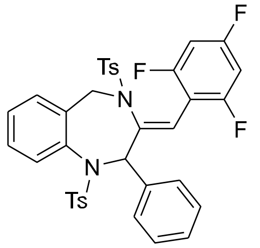

(*Z*)-7i

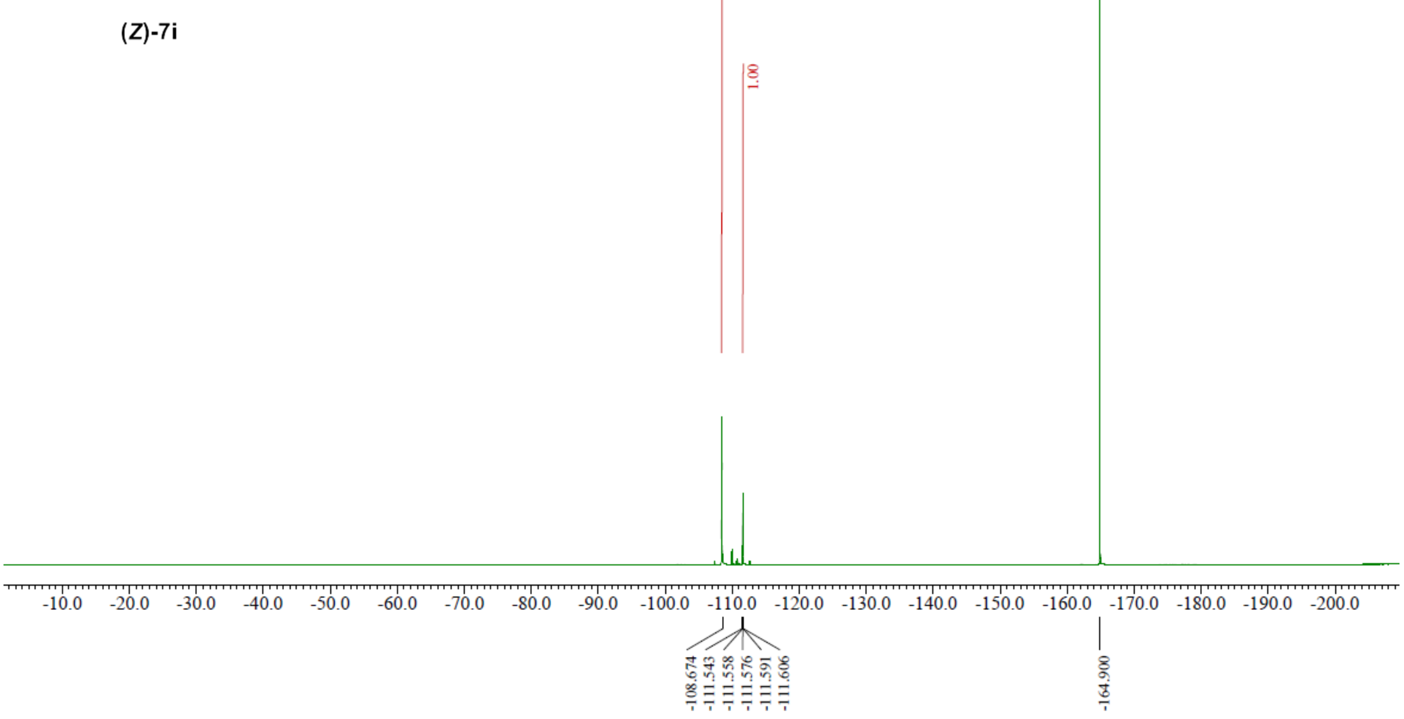

^{1}H-NMR (500 MHz, $CDCl_3$)

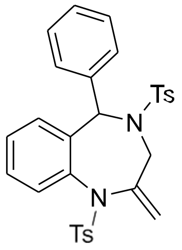

3j

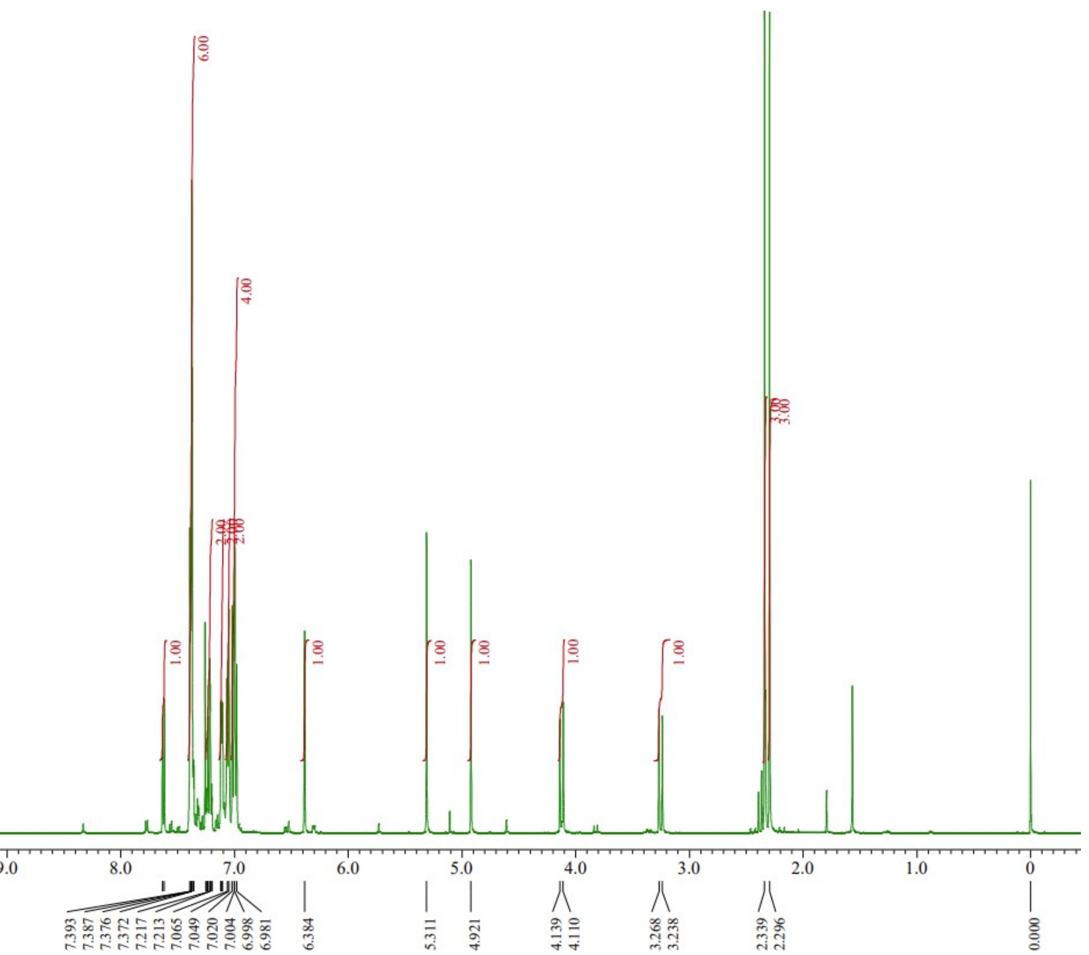

^{13}C-NMR (125 MHz, $CDCl_3$)

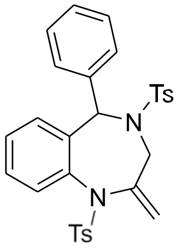

3j

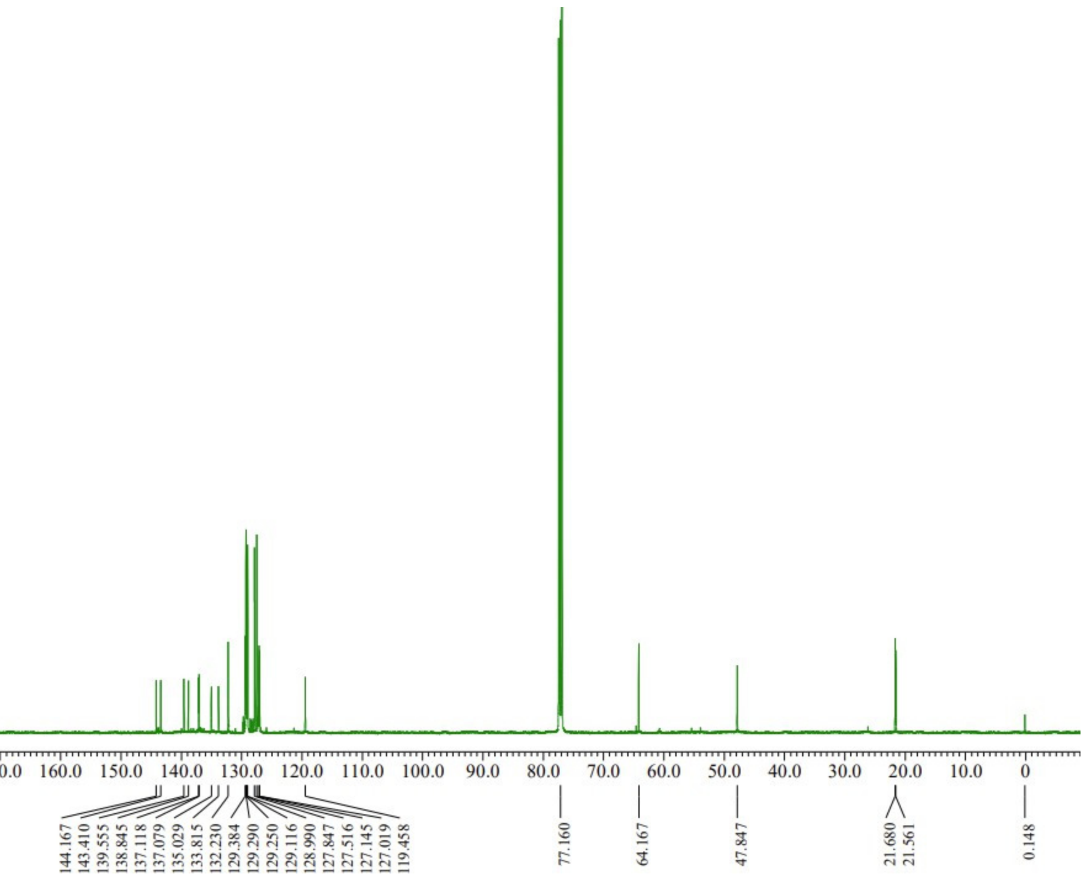

Supplement: Supplementary file 1 [file molecules-30-03004-s001.zip › molecules-3706663-supplementary.pdf]
